# 11*H*-Benzo[4,5]imidazo[1,2-*a*]indol-11-one as a New Precursor of Azomethine Ylides: 1,3-Dipolar Cycloaddition Reactions with Cyclopropenes and Maleimides

**DOI:** 10.3390/ijms232113202

**Published:** 2022-10-30

**Authors:** Alexander S. Filatov, Yulia A. Pronina, Stanislav I. Selivanov, Stanislav V. Shmakov, Anton A. Uspenski, Vitali M. Boitsov, Alexander V. Stepakov

**Affiliations:** 1 Department of Chemistry, Saint-Petersburg State University, Saint Petersburg 199034, Russia; 2 Department of Organic Chemistry, Saint-Petersburg State Institute of Technology, Saint Petersburg 190013, Russia; 3 Laboratory of Biomolecular NMR, Saint-Petersburg State University, Saint Petersburg 199034, Russia; 4 Laboratory of Nanobiotechnologies, Saint-Petersburg National Research Academic University of the Russian Academy of Sciences, Saint Petersburg 194021, Russia; 5 Department of Monitoring and Research of the Chemical Composition of the Atmosphere, Voeikov Main Geophysical Observatory, Saint Petersburg 194021, Russia

**Keywords:** 11*H*-benzo[4,5]imidazo[1,2-*a*]indol-11-one, 1,3-dipolar cycloaddition, azomethine ylides, cyclopropenes, maleimides, diastereoselectivity, DFT calculations, in vitro antiproliferative activity

## Abstract

The possibility of generating azomethine ylides from 11*H*-benzo[4,5]imidazo[1,2-a]indol-11-one and amino acids is shown for the first time. Based on the cycloaddition reactions of these azomethine ylides with cyclopropenes and maleimides, cyclopropa[a]pyrrolizines, 3-azabicyclo[3.1.0]hexanes, and pyrrolo[3,4-a]pyrrolizines spiro-fused with a benzo[4,5]imidazo[1,2-a]indole fragment were synthesized. Spirocyclic compounds were obtained in moderate to good yields, albeit with poor diastereoselectivity. Density functional theory calculations were performed to obtain an insight into the mechanism of the 1,3-dipolar cycloaddition of 11H-benzo[4,5]imidazo[1,2-*a*]indol-11-one-derived azomethine ylides to cyclopropenes. The cytotoxic activity of some of the obtained cycloadducts against the human erythroleukemia (K562) cell line was evaluated in vitro by MTS-assay.

## 1. Introduction

**Figure 1:**
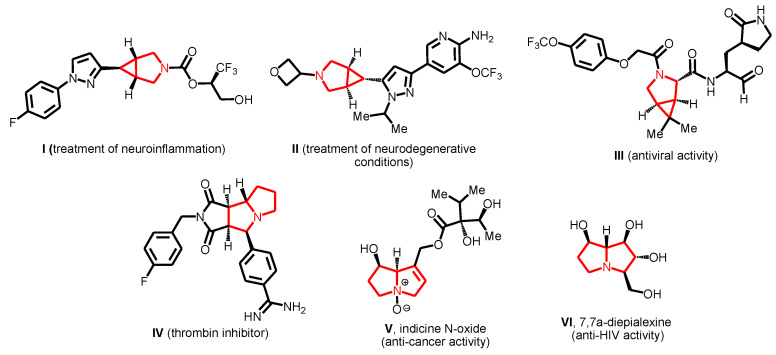
Selected examples of biologically active 3-azabicyclo[3.1.0]hexanes and pyrrolizines.

1,3-Dipolar cycloaddition (1,3-DC) is one of the most versatile methodologies for constructing five-membered heterocycles [1,2]. [3 + 2] Cycloaddition can take place with the participation of various 1,3-dipoles and dipolarophiles; however, these reactions have several features in common: (1) the dipole has a charge, (2) the resulting cycloadduct is uncharged, and (3) the HOMO of the 1,3-dipole reacts with the LUMO of the dipolarophile (a HOMO-controlled reaction). Among the 1,3-dipoles, azomethine ylides (belonging to allyl-type *N*-centered dipoles) are among the most popular and frequently used in 1,3-dipolar cycloaddition reactions [3,4]. One of the most well-known methods for the preparation of azomethine ylides is the reaction of amines with aldehydes, followed by deprotonation of the iminium cation or a prototropic shift of the imine [5,6]. Amino acids are capable of reacting with aldehydes or ketones to generate intermediate oxazolidinones, which undergo decarboxylation, resulting in the formation of azomethine ylides [7,8]. The other commonly used approaches to generate azomethine ylides rely on thermal ring-opening reactions involving aziridines or 4-oxazolines [9,10]. These and other methods continue to be widely used in synthetic organic chemistry. Reactions of azomethine ylides with alkenes make it possible to obtain highly substituted pyrrolidines containing up to four stereocenters [11,12]. The pyrrolidine ring, isolated or fused, can be attributed to a fundamental structural fragment that is part of a large number of natural and synthetic biologically-significant compounds [13]. The analysis of 164 U.S. FDA-approved small-molecule drugs from 2015 to June 2020 revealed that 22 (13%) of them contain a pyrrolidine moiety [14]. At the same time, 17 (8.5%) out of 200 small molecular drugs with the largest retail sales in 2020 contained the pyrrolidine cycle [15]. Nevertheless, despite many achievements in the study of 1,3-dipolar cycloaddition reactions, the search for new carbonyl substrates for the generation of azomethine ylides, which can lead to the construction of new heterocyclic systems containing valuable pharmacophore fragments, is highly desirable. Of the pharmacophore fragments that can be obtained through the [3 + 2] cycloaddition reactions of azomethine ylides, in our opinion, derivatives of 3-azabicyclo[3.1.0]hexane and pyrrolizine deserve special attention (Figure 1). 3-Azabicyclo[3.1.0]hexanes can be characterized as an important class of heterocyclic compounds with diverse pharmacological and biological activities. For example, 3-azabicyclo[3.1.0]hexanes exhibit anti-neuroinflammatory (monoacylglycerol lipase inhibitor, (**I**) [16], anti-neurodegenerative (dual leucine zipper kinase inhibitor, (**II**) [17] and antiviral (SARS-CoV-2 main protease inhibitor, (**III**) [18,19] effects. It is well known that pyrrolizine is the main structural moiety in many organic compounds exhibiting various biological activities, including anticoagulant (serine protease thrombin inhibitor, (**IV**) [20], anti-cancer (indicine N-oxide, (**V)**, is an antitumor agent for pediatric cancer and solid tumors research)^14^ and anti-HIV activities (7,7a-diepialexine, (**VI**) [21] (Figure 1).

**Scheme 1:**
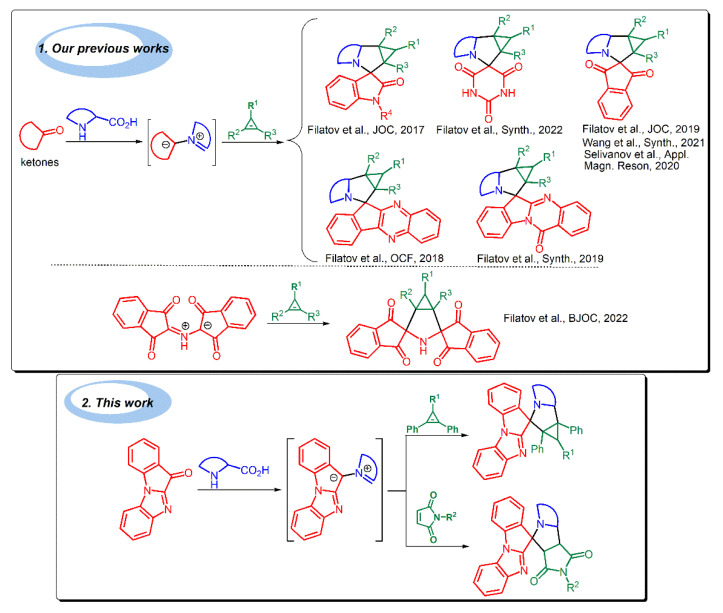
Our previous studies on 1,3-dipolar cycloaddition of azomethine ylides to cyclopropenes [22,23,24,25,26,27,28,29].

In previous studies bys our research group, cyclopropenes were widely used as dipolarophiles in 1,3-dipolar cycloaddition reactions with azomethine ylides generated from the corresponding ketones and *α*-amino acids (Scheme 1). We used derivatives of isatin [22], alloxan [23], ninhydrin [24,25,26,27], 11*H*-indeno[1,2-*b*]quinoxalin-11-one [28], and tryptanthrin [29] as the ketone components. The products of these reactions were pharmacologically interesting spiro-fused 3-azabicyclo[3.1.0]hexanes and pyrrolizines, some of which were identified as exhibiting in vitro antitumor activity [30,31].

As we noted above, the search for new substrates for the generation of azomethine ylides is an urgent task, since it allows introducing new unique poly/spirocyclic systems into the arsenal of heterocyclic chemistry, which may be of interest for pharmacology. With our continued research interest in 1,3-dipolar cycloaddition reactions, herein we present the first example of the generation of azomethine ylides from the tetracyclic ketone 11*H*-benzo[4,5]imidazo[1,2-*a*]indol-11-one and an α-amino acids, and also its [3 + 2] cycloaddition with cyclopropenes and maleimides (Scheme 1). It is known that indole-fused heterocycles, such as benzoimidazo[1,2-*a*]indole derivatives, are structural fragments of numerous natural products and pharmacologically active compounds [32,33,34,35,36]. Previously, Lee and coworkers [37,38] described the functionalization of 11*H*-benzo[4,5]imidazo[1,2-*a*]indol-11-one, which resulted in compounds of interest for the creation of phosphorescent organic light-emitting devices.

## 2. Results and Discussion

### 2.1. Synthesis

In initial studies, the possibility of 1,3-dipolar cycloaddition was investigated by performing a model three component reaction of cyclopropene **1a**, 11*H*-benzo[4,5]imidazo[1,2-*a*]indol-11-one (**2**), and *L*-proline (**3a**) in various solvents (Table 1). In methanol or ethanol, only trace amounts of cycloadduct **4a/5a** could be detected (Table 1, entries 1 and 2); when the reaction was carried out in THF or acetonitrile, the desired product **4a/5a** was obtained in 21% or 52%, respectively, after 18 h (Table 1, entries 3 and 4). At the same time, when carrying out the reaction in low-polarity dioxane, it was possible to increase the yield of the target product to 64%; however, in this case, as in all others, a low level of diastereoselectivity was observed (*dr* 2:1) (Table 1, entry 5). The use of non-polar solvents such as benzene or toluene did not increase the yield (Table 1, entries 6 and 7). Non-polar solvents such as benzene or toluene did not facilitate an increase of the yield (Table 1, entries 6 and 7). Through the experiments above, we finally confirmed the optimal reaction conditions: **1** (0.3 mmol), **2** (0.3 mmol), **3** (0.6 mmol), and 1,4-dioxane (4 mL) in a sealed tube at 100 °C for 18 h under a nitrogen atmosphere.

To explore the scope of the reaction, a range of cyclopropene dipolarophiles 1a-h was tested in the reaction with azomethine ylide generated from ketone 2 and *L*-proline (3a) under optimized conditions. The results are summarized in Table 2.

**Figure 2:**
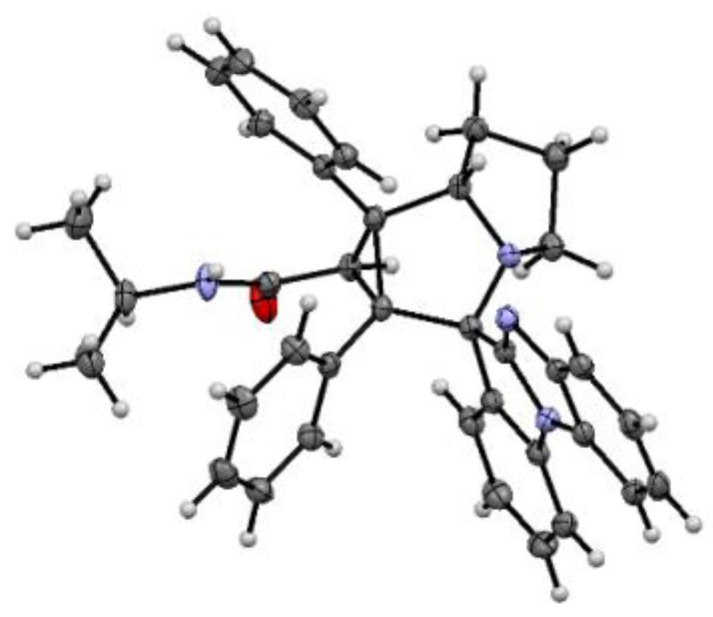
ORTEP representation of the molecular structure of **4f** (CCDC 2195042).

In addition to product 4a, various cycloadducts can be prepared as mixtures of diastereomers with good conversion and low *dr* (Table 2). In most cases, the diastereomers are chromatographically inseparable; however, the major *endo*-diastereomer 4 can be isolated by crystallization of the mixture from methanol (Table 2, 4a-c,e-h). Only in the case of cycloadduct 4d/5d derived from trimethylsilylcyclopropene 1d were both diastereomers 4d and 5d successfully separated by preparative TLC. It is important to note that cyclopropenes with both electron-rich and electron-deficient substituents R^1^ at the C3 position turned out to be reactive towards this azomethine ylide. The corresponding cycloadducts 4a-h were obtained as mixtures of two diastereomers in overall yields of 58–83% (Table 2). No significant effect of the R^1^ substituents on the course of the reactions was observed. The structures of major diastereomers 4 were unambiguously confirmed by NMR spectroscopy and single-crystal X-ray analysis of 4f (Figure 2). The stereochemistry of the minor diastereomer 5d was determined using 2D NMR spectroscopy (see the Supporting Information, Figures S53–S59). As can be seen, the diastereomers 4 and 5 differ from each other in the configuration of the spiro atom. Attempts to carry out this reaction with other secondary *α*-amino acids such as sarcosine, azetidine-2-carboxylic acid, pipecolic acid, and thiazolidine-4-carboxylic acid were unsuccessful. It is noteworthy that only cyclopropenes containing phenyl substituents at the double bond can be used in this reaction. Unsubstituted and methyl-substituted cyclopropenes proved to be ineffective as dipolarophiles in this reaction.

**Scheme 2:**
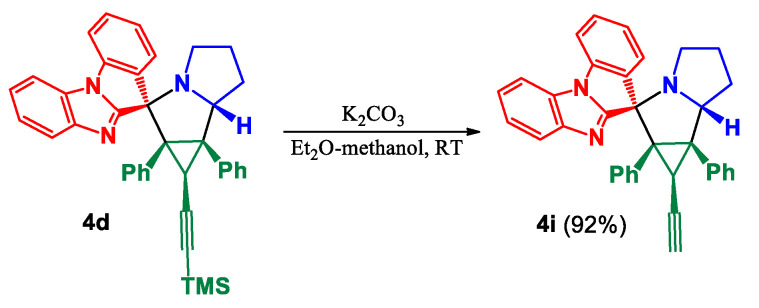
Removal of the trimethylsilyl group from 4d.

The trimethylsilyl group of 4d was readily removed by K_2_CO_3_ in methanol and ether to obtain cyclopropylacetylene 4i in a 92% yield (Scheme 2).

Upon further study of the substrate scope, we focused on the *α*-amino acid component (Table 3). First, we performed a reaction between cyclopropene 1a and azomethine ylide generated in situ from ketone 2 and 2-aminobutanoic acid (3b). As a result, spiro[3-azabicyclo[3.1.0]hexane] 6a was obtained as a chromatographically inseparable mixture of two diastereomers in 46% yield with low *dr* (2.6:1). The major *endo*-diastereomer can be obtained in pure form by crystallization from methanol. The reaction between cyclopropene 1a, ketone 2, and different alkyl-substituted amino acids (*DL*-norvaline (3c), *L*-methionine (3d), *DL*-norleucine (3e), and *L*-leucine (3f)) proceeded smoothly under the optimal reaction conditions and produced the expected spiro cycloadducts 6b–e in moderate isolated yields (42–53%) (Table 3). As in the previous case, these products are formed as a mixture of diastereomers, from which the main component can be isolated by crystallization from methanol. Subsequently, the cyclopropene scope of the 1,3-dipolar cycloaddition was investigated, employing ketone 2 and *L*-leucine (3f) as precursors of the azomethine ylide. It should be noted that cyclopropenes 1b–d,f,h give the corresponding products 6f–j in moderate yields, regardless of the electronic effect of the substituent at C*3* (Table 3). Summarizing the data given in Table 3, it should be noted that the nature of the substituent at the cyclopropene ring does not significantly affect the yield of the target spiro adducts nor the stereochemistry of cycloaddition. The stereochemistry of the major diastereomer is identical to the stereochemistry of the cycloadducts obtained from cyclopropenes and azomethine ylides based on isatin, 11*H*-indeno[1,2-*b*]quinoxalin-11-one, and tryptanthrin (previously described in a series studies) [22,28,29]. In the case of other primary *α*-amino acids such as glycine, alanine, valine, 2-aminocaprylic acid, phenylglycine, phenylalanine, and tryptophan, the corresponding cycloadducts could not be obtained.

**Figure 3:**
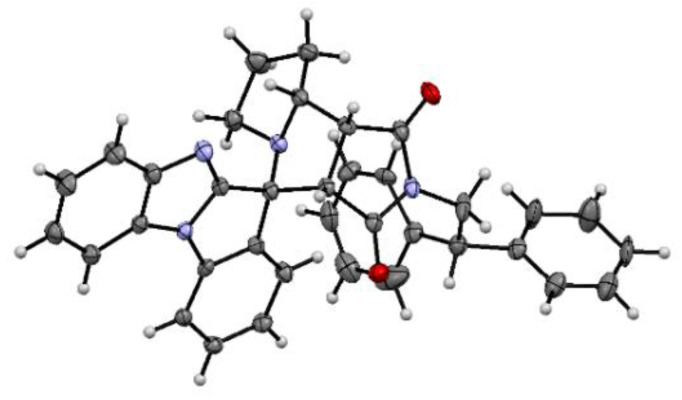
ORTEP representation of the molecular structure of **8f** (CCDC 2195043).

As shown above, cyclopropenes can successfully act as dipolarophiles in reactions with azomethine ylides derived from ketones and amino acids, which allowed us to expand the structural diversity of dipoles capable of reacting with cyclopropenes. However, other dipolarophiles are also of interest for studying their reactivity towards new azomethine ylides. We turned our attention to the maleimides, classical dipolarophiles well-studied in many 1,3-dipolar cycloaddition reactions. Various maleimides 7 were tested in the reaction with azomethine ylide generated from 2 and 3a, and the results are summarized in Table 4. Optimization of the reaction conditions showed that the best yield of cycloadducts was achieved when the reactions were carried out in an acetonitrile medium. The three-component reaction involving parent maleimide (7a) proceeded in acetonitrile at 100 °C for 18 h with the formation of *endo*-product 8a in 78% yield with 1.5:1 diastereoselectivity (Table 4). Reaction of ketone 2, *L*-proline (3a) and *N*-methyl maleimide (7b) afforded 8b in 69% yield with low diastereoselectivity (1.7:1 *dr*). Interestingly, *N*-phenyl maleimide (7c) resulted in an increase in diastereoselectivity (8c, 9:1 *dr*). The higher diastereoselectivity can be explained by the π-π interaction between the phenyl group of maleimide 7c and the aromatic moiety of azomethine ylide. *N*-Alkylmaleimides 7d–f also reacted smoothly with the azomethine ylide generated from 2 and 3a to form the desired *endo*-products 8d–f in high yields (68−89%), albeit with low diastereoselectivity. The structure and relative configuration of 8f were unambiguously determined by X-ray diffraction analysis (Figure 3). In line with the structure of 8f, this relative configuration was assigned to other major cycloadducts 8. The minor diastereomer is an epimer and differs in its spiro atom configuration.

### 2.2. Computational Study

**Figure 4:**
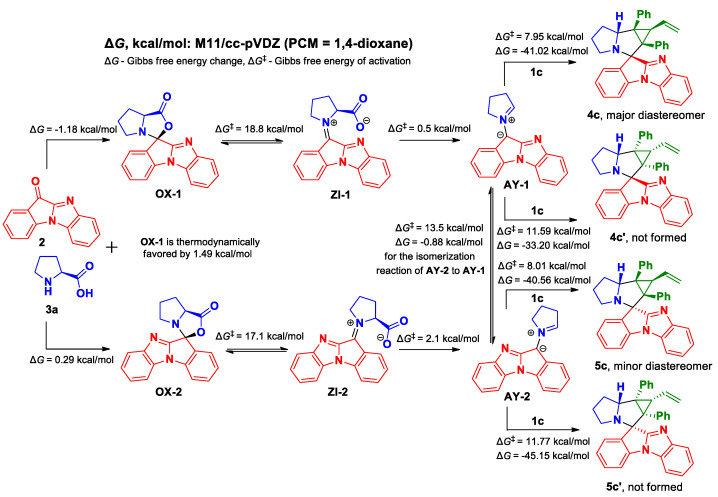
Plausible reaction mechanism for the reaction of 1,2-diphenyl-3-vinylcyclopropene (**1c**), 11H-benzo[4,5]imidazo[1,2-a]indol-11-one (**2**), and L-proline (**3a**).

Given that 11H-benzo[4,5]imidazo[1,2-a]indol-11-one (**2**) had not previously been studied as a precursor of azomethine ylides, we considered it necessary to carry out a density functional theory (DFT) computational study that would enable obtaining a comprehensive view of the reaction mechanisms of azomethine ylide formation and cycloaddition between 1,2-diphenyl-3-vinylcyclopropene (**1c**) and azomethine ylide generated from **2** (Figure 4). Moreover, this calculation data may provide further evidence of the relative configuration of both resulting diastereomers **4c** and **5c**.

Initially, we calculated the Gibbs free energy change for the reactions of azomethine ylide **AY-1** (S-shaped) and **AY-2** (W-shaped) formation from tetracyclic ketone (**2**) and L-proline (**3a**). These reactions are exergonic processes taking place with a decrease in the Gibbs free energy (ΔG = –7.3 kcal/mol for **AY-1**, ΔG = –6.4 kcal/mol for **AY-2**). As was established in studies [39,40] regarding the decarboxylative route to azomethine ylides, the first intermediate is an oxazolidin-5-one derivative. The latter is converted into a nitrogen ylide as a result of stereospecific 1,3-dipolar cycloreversion. In this case, the condensation reaction of **2** and **3a** brings about the formation of two diastereomeric oxazolidin-5-ones, **OΧ-1** and **OΧ-2,** which are precursors of **AY-1** (S-shaped) and **AY-2** (W-shaped) ylides, respectively. Based on the calculation data, the 1,3-dipolar cycloreversion of oxazolidin-5-ones **OX-1** and **OX-2,** leading to 1,3-dipoles **AY-1** and **AY-2**, respectively, was found to occur in a two-step manner. Moreover, the first step is rate-determining. Considering this rate-determining step, the small difference (0.2 kcal/mol) in activation free energy values reveals the fact that the azomethine ylide is formed as a mixture of two isomers **AY-1** and **AY-2** in an almost equimolar ratio. Due to a partial double bond character of a carbon–nitrogen bond, one conformer of an azomethine ylide may isomerize to another. However, in this case, this process does not take place, because the cycloaddition reactions involving cyclopropene **1c** and ylides **AY-1** (ΔG^‡^_1c + AY-1->4c_ = 7.95 kcal/mol), **AY-2** (ΔG^‡^_1c + AY-2->5c_ = 8.01 kcal/mol) are kinetically much more favorable than the isomerization reaction (ΔG^‡^_AY-2->AY-1_ = 13.5 kcal/mol). Thus, **AY-2** is not capable of fully converting into the more stable conformer **AY-1,** since both ylides are immediately trapped by cyclopropene **1c**. It is remarkable that each of these ylides produces only one cycloadduct. According to the calculation data, cycloaddition reactions of 1,2-diphenyl-3-vinylcyclopropene (**1c**) to both forms of the azomethine ylide, **AY-1** and **AY-2,** occur strictly via endo transition states (ΔG^‡^_1c + AY-1 ->4c_ = 7.95 kcal/mol and ΔG^‡^_1c + AY-2 ->5c_ = 8.01 kcal/mol), which significantly prevail over exo ones (ΔG^‡^_1c + AY-1->4c’_ = 11.59 kcal/mol and ΔG^‡^_1c + AY - 2- >5c’_ = 11.77 kcal/mol). Of the two endo pathways, the endo approach involving ylide **AY-1** is more favorable than the other endo approach (ca. 0.9 kcal/mol). The calculation data are completely consistent with experimental results. Actually, cycloadduct **4c**, the precursor of which is the more thermodynamically stable ylide **AY-1**, predominates in the mixture of diastereomers **4c** and **5c**. In the meantime, minor diastereomer **5c** results from the endo approach of ylide **AY-2** to cyclopropene **1c**. Probably, these findings hold true for other cycloaddition reactions between cyclopropenes **1** and azomethine ylide generated from **2** and **3a**.

### 2.3. Biological Assay

**Figure 5:**
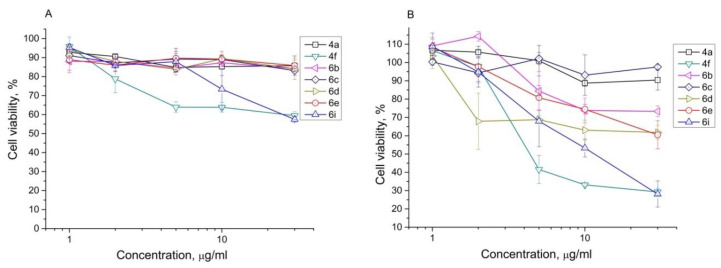
Cytotoxicity of Selected Racemic Spiro-Adducts **4** and **6** against the K562 cell line for 24 h (**A**) and 72 h (**B**).

The antiproliferative activity of some of the synthesized spiro-fused 11H-benzo[4,5]imidazo[1,2-a]indol-11-ones and cyclopropa[a]pyrrolysines **4** or 3-azabicyclo[3.1.0]hexanes **6** against human erythroleukemia (K562) cell line was evaluated in vitro using the standard MTS assay for 24 and 72 h. The results of these investigations are presented in Figure 5. It was found that spiroadducts with the N-isopropylcarbamoyl group at the cyclopropane moiety were more active, while replacement of this group with phenyl one led to a significant decrease in this activity. Thus, it was found that among the tested compounds, spiroadducts **4f** and **6i** demonstrated a significant activity, with IC_50_ 5 ± 1 and 12 ± 2 μg/mL, respectively.

## 3. Materials and Methods

### 3.1. Chemistry

NMR spectra were recorded with a Bruker Avance 400 spectrometer (400.13 MHz for ^1^H and 100.61 MHz for ^13^C). Chemical shifts are reported in ppm relative to residual CHCl_3_ (^1^H, *δ* = 7.26 ppm), CDCl_3_ (^13^C, *δ* = 77.17), residual DMSO-*d*_5_ (^1^H, 2.50 ppm), and DMSO-*d*_6_ (^13^C, 39.52 ppm) as internal standards. Mass spectra were recorded on a HRMS-ESI-QTOF mass-analyzer, electrospray ionization, positive mode. Single-crystal X-ray diffraction experiments for compounds **4f** and **8f** were carried out using an Agilent Technologies «Xcalibur» diffractometer with monochromated Mo Kα radiation. Melting points were determined on a melting point apparatus and are uncorrected. 11*H*-Benzo[4,5]imidazo[1,2-*a*]indol-11-one was obtained from 1*H*-benzo[*d*]imidazole and 2-fluorobenzaldehyde, according to a known method [37]. Cyclopropenes **1a** [41], **1b**,**c** [42], **1d** [43], **1e** [44], **1f**,**g** [45], and **1h** [46] were prepared according to the literature data, while α-amino acids **3** were obtained from commercial sources. Maleimides **7e** [47] and **7f** [48] were synthesized according to previously described procedures, **7a−d** were obtained from commercial sources. Crystallographic data for compounds **4f** (CCDC 2195042) and **8f** (CCDC 2195043) have been deposited at the Cambridge Crystallographic Data Centre and can be obtained free of charge via www.ccdc.cam.ac.uk/data_request/cif (accessed on 5 August 2022). Copies of ^1^H and ^13^C NMR spectra of compounds **4-6** and **8** are given at Supporting Information, (Figures S1–S52). X-ray crystallographic data for compounds **4f** and **8f** are given at Supporting Information, (Figures S60, S61 and Tables S1 and S2).

**General Procedure A for the One-Pot Three-Component Reaction of 11H-Benzo[4,5]imidazo[1,2-*a*]indol-11-one, *L*-Proline, and Cyclopropenes**

11*H*-Benzo[4,5]imidazo[1,2-*a*]indol-11-one (**2**, 0.3 mmol), *L*-proline (**3a**, 0.6 mmol), cyclopropene (**1**, 0.3 mmol), and anhydrous 1,4-dioxane (4 mL) were added to a screw-capped tube (Schuett-biotec type). The reaction vessel was closed and the reaction mixture was stirred in a preheated oil bath at 100 °C for 18 h (the reaction progress was monitored by TLC). The mixture was cooled to room temperature, and the solvent was removed under reduced pressure. The residue was subjected to silica gel PTLC using a mixture of hexane–ethyl acetate as an eluent to give the spiro-adducts **4a−h**.

**(±)-(1’*R*,1a’*R*,2’*R*,6a’*S*,6b’*S*)-1’,1a’,6b’-Triphenyl-1a’,4’,5’,6’,6a’,6b’-hexahydro-1’*H*-spiro[benzo[4,5]imidazo[1,2-*a*]indole-11,2’-cyclopropa[*a*]pyrrolizine] (4a) and (1’*R*,1a’*R*,2’*S*,6a’*S*,6b’*S*)-1’,1a’,6b’-triphenyl-1a’,4’,5’,6’,6a’,6b’-hexahydro-1’*H*-spiro[benzo[4,5]imidazo[1,2-*a*]indole-11,2’-cyclopropa[*a*]pyrrolizine] (5a)**

Prepared according to the general procedure **A** using cyclopropene **1a** (85.3 mg, 0.32 mmol), ketone **2** (70.0 mg, 0.32 mmol) and *L*-proline (**3a**) (73.2 mg, 0.64 mmol). Purification of the crude by PTLC (hexane–ethyl acetate, 3:1) furnished **4a** and **5a** (112 mg, 64%) as an inseparable mixture in ratio 2:1, respectively. Crystallization of the mixture from methanol gave the major diastereomer **4a** as a pure compound (67 mg);

Data for **4a**: white solid; mp 170–172 °C; R*_f_* 0.64 (SiO_2_, hexane–ethyl acetate, 3:1).

IR (KBr): 3056, 2962, 2817, 1608, 1525, 1491, 1471, 1444, 1345, 1302, 1222, 1167, 772, 737, 706, 556, 534 cm^−1^.

^1^H NMR (400 MHz, CDCl_3_): δ = 7.99 (t, *J* = 7.7 Hz, 2 H), 7.61 (d, *J* = 7.7 Hz, 2 H), 7.49 (d, *J* = 7.7 Hz, 1 H), 7.40 (t, *J* = 7.7 Hz, 1 H), 7.33–7.22 (m, 7 H), 7.02 (t, *J* = 7.7 Hz, 1 H), 6.94 (t, *J* = 7.7 Hz, 2 H), 6.75 (br.s, 2 H), 6.73 (t, *J* = 7.7 Hz, 1 H), 6.60 (t, *J* = 7.7 Hz, 2 H), 6.48 (d, *J* = 7.7 Hz, 2 H), 4.81 (t, *J* = 7.0 Hz, 1 H), 3.70 (s, 1 H), 3.21 (q, *J* = 7.0 Hz, 1 H), 2.57(m, 1 H), 2.20 (m, 1 H), 2.03 (m, 1 H), 1.90–1.80 (m, 2 H).

^13^C NMR (101 MHz, CDCl_3_): δ = 162.3, 148.2, 138.9, 137.1, 134.7, 134.4, 132.3 (4 C), 131,3, 130.9 (2 C), 129.4, 129,1, 127.7 (2 C), 126.5 (2 C), 126.5, 126.4 (2 C), 126.4, 126.2, 125.0, 123.8, 122.7, 122.1, 120.7, 110.5, 110.2, 75.7, 71.4, 57.6, 46.5, 45.1, 31.3, 26.6, 25.0.

HRMS (ESI): *m*/*z* [M+H]^+^ calcd for C_39_H_32_N_3_: 542.2591; found: 542.2592.

**(±)-(1’*R*,1a’*R*,2’*R*,6a’*S*,6b’*S*)-1’-Ethyl-1a’,6b’-diphenyl-1a’,4’,5’,6’,6a’,6b’-hexahydro-1’*H*-spiro[benzo[4,5]imidazo[1,2-*a*]indole-11,2’-cyclopropa[*a*]pyrrolizine] (4b) and (±)-(1’*R*,1a’*R*,2’*S*,6a’*S*,6b’*S*)-1’-ethyl-1a’,6b’-diphenyl-1a’,4’,5’,6’,6a’,6b’-hexahydro-1’*H*-spiro[benzo[4,5]imidazo[1,2-*a*]indole-11,2’-cyclopropa[*a*]pyrrolizine] (5b)**

Prepared according to the general procedure **A** using cyclopropene **1b** (70.5 mg, 0.32 mmol), ketone **2** (70.0 mg, 0.32 mmol), and *L*-proline (**3a**) (73.2 mg, 0.64 mmol). Purification of the crude by PTLC (hexane–ethyl acetate, 5:1) furnished **4b** and **5b** (131 mg, 83%) as an inseparable mixture in a ratio of 2.2:1, respectively. Crystallization of the mixture from methanol gave the major diastereomer **4b** as a pure compound (81 mg).

Data for **4b**: white solid; mp 220–222 °C; R*_f_* 0.59 (SiO_2_, hexane–ethyl acetate, 3:1).

IR (KBr): 3056, 2963, 2905, 2836, 1609, 1523, 1492, 1472, 1445, 1347, 1306, 1222, 1166, 922, 748, 737, 699 cm^−1^.

^1^H NMR (400 MHz, CDCl_3_): δ = 7.89 (d, *J* = 7.5 Hz, 1 H), 7.87–7.80 (m, 1 H), 7.52–7.42 (m, 4 H), 7.36–7.27 (m, 4 H), 7.26–7.18 (m, 3 H), 6.88–6.80 (m, 3 H), 6.76 (t, *J* = 7.5 Hz, 2 H), 4.79 (br.m, 1 H), 3.05–2.98 (m, 1 H), 2.50–2.38 (m, 2 H), 2.33–2.11 (m, 2 H), 2.11–1.99 (m, 1 H), 1.89–1.75 (m, 1 H), 1.63–1.49 (m, 1 H), 1.47–1.34 (m, 1 H), 1.02 (t, *J* = 7.0 Hz, 3 H).

^13^C NMR (101 MHz, CDCl_3_): δ = 162.4, 148.0, 138.9, 137.7, 135.3, 132.7, 131.6 (2 C), 129.9 (2 C), 129.3, 129.1, 127.5 (2 C), 126,7 (2 C), 126.4, 126.0, 125.6, 123.7, 122.6, 122.0, 120.6, 110.5, 110.1, 71.7, 70.5, 54.4, 44.4, 41.7, 29.6, 26.8, 26.6, 19.2, 14.2.

HRMS (ESI): *m*/*z* [M+H]^+^ calcd for C_35_H_32_N_3_: 494.2591; found: 494.2597.

**(±)-(1’*R*,1a’*R*,2’*R*,6a’*R*,6b’*S*)-1a’,6b’-Diphenyl-1’-vinyl-1a’,4’,5’,6’,6a’,6b’-hexahydro-1’*H*-spiro[benzo[4,5]imidazo[1,2-a]indole-11,2’-cyclopropa[a]pyrrolizine] (4c) and (±)-(1’R,1a’R,2’S,6a’R,6b’S)-1a’,6b’-diphenyl-1’-vinyl-1a’,4’,5’,6’,6a’,6b’-hexahydro-1’H-spiro[benzo[4,5]imidazo[1,2-a]indole-11,2’-cyclopropa[a]pyrrolizine] (5c)**

Prepared according to general procedure **A** using cyclopropene **1c** (69.9 mg, 0.32 mmol), ketone **2** (70.0 mg, 0.32 mmol), and *L*-proline (**3a**) (73.2 mg, 0.64 mmol). Purification of the crude by PTLC (hexane–ethyl acetate, 5:1) furnished **4c** and **5c** (101 mg, 64%) as an inseparable mixture in a ratio of 2.3:1, respectively.

Data for mixture **4c** + **5c**: white solid; mp 218–220 °C.

IR (KBr): 3055, 2967, 2910, 2869, 2809, 1610, 1526, 1493, 1473, 1446, 1348, 1223, 1168, 998, 902, 740, 699, 443 cm^−1^.

^1^H NMR (400 MHz, CDCl_3_): δ = 8.00 (d, *J* = 7.5 Hz, 1 H(**5c**)), 7.97 (d, *J* = 7.5 Hz, 1 H(**4c**)), 7.90 (d, *J* = 7.5 Hz, 1 H(**4c**)),7.86 (d, *J* = 7.5 Hz, 2 H(**4c**)), 7.85 (d, *J* = 7.5 Hz, 1 H(**5c**)), 7.65 (d, *J* = 7.5 Hz, 2 H(**5c**)), 7.59 (d, *J* = 7.5 Hz, 1 H(**5c**)), 7.49 (d, *J* = 7.5 Hz, 1 H(**4c**)), 7.45–7.21 (m, 8 H(**4c** +**5c**)), 7.17 (d, *J* = 7.5 Hz, 1 H(**5c**)), 6.97 (d, *J* = 7.5 Hz, 2 H(**4c**)), 6.82–6.76 (m, 1 H(**4c**) + 1 H(**5c**)), 6.75–6.65 (m, 2 H(**4c**) + 4 H(**5c**)), 5.62 (dd, *J* = 17.0, 2.5 Hz, 1 H(**5c**)), 5.49 (dd, *J* = 17.0, 2.5 Hz, 1 H(**4c**)), 5.16 (dt, *J* = 17.0, 10.3 Hz, 1 H(**4c**)), 5.05 (dd, *J* = 10.3, 2.5 Hz, 1 H(**4c**)), 5.01 (dd, *J* = 10.3, 2.5 Hz, 1 H(**5c**)), 4.95 (dt, *J* = 17.0, 10.3 Hz, 1 H(**5c**)), 4.76 (dd, *J* = 8.5, 6.0 Hz, 1 H(**4c** + **5c**)), 4.12 (br.q, *J* = 7.2 Hz, 1 H(**5c**)), 3.30 (d, *J* = 9.6 Hz, 1 H(**5c**)), 3.25 (d, *J* = 10.6 Hz, 1 H(**4c**)), 3.10 (q, *J* = 7.2 Hz, 1 H(**4c**)), 2.80 (br.t, *J* = 8.0 Hz, 1 H(**5c**)), 2.50 (td, *J* = 8.0, 4.0 Hz, 1 H(**4c**)), 2.27–1.91 (m, 3 H(**4c** + **5c**)), 1.91–1.73 (m, 2 H(**4c** + **5c**)).

^13^C NMR (101 MHz, CDCl_3_): δ = 162.2 (**4c**), 148.2 (**4c**), 138.9 (**4c**), 138.0 (**4c**), 136.6 (**4c**), 134.6 (**4c**), 132.6, 132.1 (2 C (**4c**)), 131.4 (2 C(**4c**)), 131.1 (**4c**), 129.4 (**4c**), 129.2 (**4c**), 128.1 (**4c**), 127.9 (2 C (**4c**)), 126.8 (**4c**), 126.7 (2 C), 126.4, 126.3 (**4c**), 124.3 (**5c**), 123.8 (**4c**), 123.3 (**5c**), 122.7 (**4c**), 122.4 (**5c**), 122.1 (**4c**), 120.8 (**5c**), 120.7 (**4c**), 114.1 (**5c**), 113.3 (**4c**), 110.5 (**4c**), 110.4 (**5c**), 110.2 (**4c**), 110.1 (**5c**), 74.3 (**4c**), 70.9 (**4c**), 55.2 (**4c**), 49.4 (**4c**), 45.0 (**4c**), 44.9 (**4c**), 32.2 (**5c**), 30.7 (**4c**), 27.8 (**5c**), 27.2 (**5c**), 26.6 (**4c**), 25.1 (**4c**).

HRMS (ESI): *m*/*z* [M+H]^+^ calcd for C_35_H_30_N_3_: 492.2434; found: 492.2439.

**(±)-(1’*R*,1a’*R*,2’*R*,6a’*R*,6b’*S*)-1a’,6b’-Diphenyl-1’-((trimethylsilyl)ethynyl)-1a’,4’,5’,6’,6a’,6b’-hexahydro-1’*H*-spiro[benzo[4,5]imidazo[1,2-a]indole-11,2’-cyclopropa[a]pyrrolizine] (4d) and (±)-(1’R,1a’R,2’S,6a’R,6b’S)-1a’,6b’-diphenyl-1’-((trimethylsilyl)ethynyl)-1a’,4’,5’,6’,6a’,6b’-hexahydro-1’H-spiro[benzo[4,5]imidazo[1,2-a]indole-11,2’-cyclopropa[a]pyrrolizine] (5d)**

Prepared according to general procedure **A** using cyclopropene **1d** (92.3 mg, 0.32 mmol), ketone **2** (70.0 mg, 0.32 mmol), and *L*-proline (**3a**) (73.2 mg, 0.64 mmol). Purification of the crude by PTLC (hexane–ethyl acetate, 3:1) furnished **4d** and **5d** as separated diastereomers.

Data for major diastereomer **4d**: yield 101 mg (56%); white solid; mp 189–191 °C; R*_f_*0.60 (SiO2, hexane–ethyl acetate, 3:1).

IR (KBr): 3057, 2969, 2835, 2152, 1609, 1526, 1492, 1471, 1446, 1344, 1250, 1223, 849, 840, 735, 697 cm^−1^.

^1^H NMR (400 MHz, CDCl_3_): δ = 7.91–7.84 (m, 2 H), 7.65 (d, *J* = 7.5 Hz, 2 H), 7.52–7.47 (m, 1 H), 7.44 (t, *J* = 7.5 Hz, 1 H), 7.37–7.20 (m, 7 H), 6.95 (d, *J* = 7.5 Hz, 2 H), 6.86 (t, *J* = 7.5 Hz, 1 H), 6.76 (t, *J* = 7.5 Hz, 2 H), 4.85 (dd, *J* = 5.0, 9.0 Hz, 1 H), 3.42 (s, 1 H), 2.92–2.82 (m, 1 H), 2.44–2.33 (m, 1 H), 2.22–1.98 (m, 3 H), 1.85–1.70 (m, 1 H), −0.05 (s, 9 H).

^13^C NMR (101 MHz, CDCl_3_): δ = 160.9, 147.9, 138.7, 135.2, 134.0, 132.3 (2 C), 130.6, 130.1 (2 C), 129.5, 129.2, 127.1 (2 C), 126.7, 126,5 (2 C), 126.2, 125.9, 124.1, 122.8, 122.2, 120.6, 110.5, 110.2, 104.2, 92.0, 70.7, 69.6, 56.5, 43.6, 43.0, 26.5, 25.3, 19.0, −0.5 (3 C).

HRMS (ESI): *m*/*z* [M+H]^+^ calcd for C_38_H_36_N_3_Si: 562.2673; found: 562.2679.

Data for minor diastereomer **5d**: yield 11 mg (6%); white solid; mp 242–245 °C; R*_f_* 0.48 (SiO_2_, hexane–ethyl acetate, 3:1).

IR (KBr): 3057, 2969, 2954, 2835, 2154, 1609, 1492, 1471, 1344, 1223, 850, 735, 697 cm^−1^.

^1^H NMR (400 MHz, CDCl_3_): δ = 8.06–7.99 (m, 1 H), 7.66–7.56 (m, 2 H), 7.50 (d, *J* = 7.5 Hz, 2 H), 7.43–7.35 (m, 2 H), 7.35–7.25 (m, 3 H), 7.24–7.13 (m, 3 H), 6.87 (t, *J* = 7.5 Hz, 1 H), 6.75 (t, *J* = 7.5 Hz, 2 H), 6.68 (d, *J* = 7.5 Hz, 2 H), 4.97 (br.s, 1 H), 3.89 (br.s, 1 H), 3.29 (s, 1 H), 2.73 (br.s, 1 H), 2.31 (br.s, 1 H), 2.22 (br.s, 1 H), 2.07 (br.s, 2 H), −0.13 (s, 9 H).

^13^C NMR (101 MHz, CDCl_3_): δ = 158.0, 147.8, 141.0, 136.6, 135.1, 132.1 (2 C), 131.0, 129.9 (2 C), 129.3, 128.5, 127.3 (2 C), 126.5 (4 C), 125.6, 124.1, 123.4, 122.5, 120.9, 110.5, 110.2, 103.4, 92.1, 73.0, 72.9, 57.6, 48.6, 48.3, 28.5, 27.2, 21.8, −0.6 (3 C).

HRMS (ESI): *m*/*z* [M+H]^+^ calcd for C_38_H_36_N_3_Si: 562.2673; found: 562.2672.

**(±)-(1’*R*,1a’*R*,2’*R*,6a’*R*,6b’*S*)-Methyl 1a’,6b’-diphenyl-1a’,4’,5’,6’,6a’,6b’-hexahydro-1’*H*-spiro[benzo[4,5]imidazo[1,2-a]indole-11,2’-cyclopropa[a]pyrrolizine]-1’-carboxylate (4e) and (±)-(1’R,1a’R,2’S,6a’R,6b’S)-methyl 1a’,6b’-diphenyl-1a’,4’,5’,6’,6a’,6b’-hexahydro-1’H-spiro[benzo [4,5]imidazo[1,2-a]indole-11,2’-cyclopropa[a]pyrrolizine]-1’-carboxylate (5e)**

Prepared according to the general procedure **A** using cyclopropene **1e** (80.1 mg, 0.32 mmol), ketone **2** (70.0 mg, 0.32 mmol), and *L*-proline (**3a**) (73.2 mg, 0.64 mmol). Purification of the crude by PTLC (hexane–ethyl acetate, 3:1) furnished **4e** and **5e** (112 mg, 67%) as an inseparable mixture in a ratio of 3:1, respectively. Crystallization of the mixture from methanol gave the major diastereomer **4e** as a pure compound (71 mg).

Data for **4e**: white solid; mp 242–245 °C; R*_f_* 0.36 (SiO_2_, hexane–ethyl acetate, 3:1).

IR (KBr): 3056, 2949, 2843, 1740, 1609, 1539, 1492, 1472, 1447, 1357, 1223, 1169, 922, 741, 695, 618 cm^−1^.

^1^H NMR (400 MHz, CDCl_3_): δ = 8.00–7.90 (m, 2 H), 7.71 (d, *J* = 7.7 Hz, 2 H), 7.55–7.44 (m, 2 H), 7.42–7.25 (m, 7 H), 6.88 (d, *J* = 7.7 Hz, 2 H), 6.79 (t, *J* = 7.7 Hz, 1 H), 6.70 (t, *J* = 7.7 Hz, 2 H), 4.75 (br.s, 1 H), 3.38 (s, 3 H), 3.37 (s, 1 H), 3.08 (m, 1 H), 2.51 (br.s, 1 H), 2.14 (m, 1 H), 1.98 (m, 1 H), 1.87–1.75 (m, 2 H).

^13^C NMR (101 MHz, CDCl_3_): δ = 170.0, 161.3, 148.0, 138.9, 134.4, 133.5, 131.6, 130.7 (2 C), 130.5 (2 C), 129.8, 128.9, 127.8 (2 C), 126.8, 126.7, 126.6 (2 C), 126.3, 124.1, 123.1, 122.4, 120.7, 110.8, 110,3, 74.7, 71.1, 58.2, 51.1, 48.3, 45.0, 28.8, 26.5, 25.2.

HRMS (ESI): *m*/*z* [M+H]^+^ calcd for C_35_H_30_N_3_O_2_: 524.2333; found: 524.2333.

**(±)-(1’*R*,1a’*R*,2’*R*,6a’*R*,6b’*S*)-*N*-Isopropyl-1a’,6b’-diphenyl-1a’,4’,5’,6’,6a’,6b’-hexahydro-1’H-spiro[benzo[4,5]imidazo[1,2-a]indole-11,2’-cyclopropa[a]pyrrolizine]-1’-carboxamide (4f) and (±)-(1’R,1a’R,2’S,6a’R,6b’S)-N-isopropyl-1a’,6b’-diphenyl-1a’,4’,5’,6’,6a’,6b’-hexahydro-1’H-spiro[benzo[4,5]imidazo[1,2-*a*]indole-11,2’-cyclopropa[*a*]pyrrolizine]-1’-carboxamide (5f)**

Prepared according to the general procedure **A** using cyclopropene **1f** (88.8 mg, 0.32 mmol), ketone **2** (70.0 mg, 0.32 mmol), and *L*-proline (**3a**) (73.2 mg, 0.64 mmol). Purification of the crude by PTLC (hexane–ethyl acetate, 1:2) furnished **4f** and **5f** (141 mg, 80%) as an inseparable mixture in a ratio of 5:1, respectively. Crystallization of the mixture from methanol gave the major diastereomer **4f** as a pure compound (105 mg).

Data for **4f**: white solid; mp 242–244 °C; R*_f_* 0.69 (SiO_2_, hexane–ethyl acetate, 1:2).

IR (KBr): 3423, 3048, 2965, 2844, 1644, 1609, 1532, 1493, 1472, 1446, 1352, 1222, 1168, 930, 764, 740, 716, 699, 668, 496 cm^−1^.

^1^H NMR (400 MHz, CDCl_3_): δ = 8.05 (d, *J* = 7.7 Hz, 1 H), 7.94 (d, *J* = 7.7 Hz, 1 H), 7.88 (d, *J* = 7.7 Hz, 2 H), 7.50–7.40 (m, 4 H), 7.38–7.22 (m, 6 H), 7.03 (d, *J* = 7.7 Hz, 2 H), 6.84 (t, *J* = 7.7 Hz, 1 H), 6.75 (t, *J* = 7.7 Hz, 2 H), 4.72 (t, *J* = 6.0 Hz, 1 H), 3.69 (m, 1 H), 3.49 (d, *J* = 6.0 Hz, 1 H), 3.26 (s, 1 H), 3.19 (m, 1 H), 2.52 (m, 1 H), 2.11(m, 1 H), 2.00–1.80 (m, 3 H), 0.55 (d, *J* = 6.5 Hz, 3 H), 0.27 (d, *J* = 6.5 Hz, 3 H).

^13^C NMR (101 MHz, CDCl_3_): δ = 168.0, 161.5, 138.9, 133.7, 133.3, 131.9 (2 C), 131.5 (2 C), 131.0, 129.8 (2 C), 128.9 (2 C), 128.2 (2 C), 127.5, 127.4, 127.3 (2 C), 126.9, 124.2, 122.9, 122.3, 120.7, 110.6, 110.2, 75.2, 56.0, 47.2, 45.9, 40.7, 31.6, 26.5, 25.4, 21.6, 21.3.

HRMS (ESI): *m*/*z* [M+H]^+^ calcd for C_37_H_35_N_4_O: 551.2805; found: 551.2805.

**(±)-(1’*R*,1a’*R*,2’*R*,6a’*R*,6b’*S*)-1a’,6b’-Diphenyl-1a’,4’,5’,6’,6a’,6b’-hexahydro-1’*H*-spiro[benzo[4,5]imidazo[1,2-a]indole-11,2’-cyclopropa[a]pyrrolizine]-1’-carbonitrile (4g) and (±)-(1’R,1a’R,2’S,6a’R,6b’S)-1a’,6b’-diphenyl-1a’,4’,5’,6’,6a’,6b’-hexahydro-1’H-spiro[benzo[4,5]imidazo[1,2-a]indole-11,2’-cyclopropa[a]pyrrolizine]-1’-carbonitrile (5g)**

Prepared according to the general procedure **A** using cyclopropene **1g** (69.5 mg, 0.32 mmol), ketone **2** (70.0 mg, 0.32 mmol), and *L*-proline (**3a**) (73.2 mg, 0.64 mmol). Purification of the crude by PTLC (hexane–ethyl acetate, 2:1) furnished **4g** and **5g** (91 mg, 58%) as an inseparable mixture in a ratio of 4:1, respectively. Crystallization of the mixture from methanol gave the major diastereomer **4g** as a pure compound (35 mg).

Data for **4g**: beige solid; mp 247–250 °C; R*_f_* 0.39 (SiO_2_, hexane–ethyl acetate, 3:1).

IR (KBr): 3067, 2960, 2813, 2234, 1611, 1521, 1496, 1474, 1446, 1353, 1308, 1223, 1169, 924, 843, 735, 702, 478 cm^−1^.

^1^H NMR (400 MHz, CDCl_3_): δ = 7.73 (br.d, *J* = 7.7 Hz, 1 H), 7.39 (d, *J* = 7.7 Hz, 1 H), 7.33 (d, *J* = 7.7 Hz, 2 H), 7.55–7.45 (m, 2 H), 7.40(t, *J* = 7.7 Hz, 2 H), 7.35–7.25 (m, 5 H), 6.98 (d, *J* = 7.7 Hz, 2 H), 6.92 (t, *J* = 7.7 Hz, 1 H), 6.82 (t, *J* = 7.7 Hz, 2 H), 4.81 (br.s, 1 H), 3.52 (s, 1 H), 2.77 (m, 1 H), 2.35 (m, 1 H), 2.19 (m, 1 H), 2.10–1.90(m, 2 H), 1.67 (m, 1 H).

^13^C NMR (101 MHz, CDCl_3_): δ = 159.2, 147.4, 138.6, 133.0, 132.9, 131.3 (3 C), 130.2, 129.7 (2 C), 129.1, 128.2 (2 C), 127.9, 127.5, 127.4 (2 C), 125.3, 124.4, 123.4, 122.6, 120.6, 118.4, 110.9, 110.4, 70.9, 68.8, 56.4, 43.6, 41.8, 26.2, 24.5, 14.4.

HRMS (ESI): *m*/*z* [M+H]^+^ calcd for C_34_H_27_N_4_: 491.2230; found: 491.2230.

**(±)-(1a’*R*,2’*R*,6a’*R*,6b’*S*)-1a’,6b’-Diphenyl-1a’,4’,5’,6’,6a’,6b’-hexahydro-1’*H*-spiro[benzo[4,5]imidazo[1,2-a]indole-11,2’-cyclopropa[a]pyrrolizine] (4h) and (±)-(1a’R,2’S,6a’R,6b’S)-1a’,6b’-diphenyl-1a’,4’,5’,6’,6a’,6b’-hexahydro-1’H-spiro[benzo [4,5]imidazo[1,2-a]indole-11,2’-cyclopropa[a]pyrrolizine] (5h)**

Prepared according to the general procedure **A** using cyclopropene **1h** (61.5 mg, 0.32 mmol), ketone **2** (70.0 mg, 0.32 mmol), and *L*-proline (**3a**) (73.2 mg, 0.64 mmol). Purification of the crude by PTLC (hexane–ethyl acetate, 3:1) furnished **4h** and **5h** (91 mg, 61%) as an inseparable mixture in a ratio of 3:1, respectively. Crystallization of the mixture from methanol gave the major diastereomer **4h** as a pure compound (32 mg).

Data for **4h**: white solid; mp 230–231 °C; R*_f_* 0.54 (SiO_2_, hexane–ethyl acetate, 3:1).

IR (KBr): 3055, 2968, 2869, 2819, 1609, 1519, 1495, 1471, 1446, 1341, 1223, 1169, 1144, 1057, 1023, 926,775, 740, 701,619 cm^−1^.

^1^H NMR (400 MHz, CDCl_3_): δ = 7.93 (d, *J* = 7.5 Hz, 1 H), 7.88 (d, *J* = 7.5 Hz, 1 H), 7.51 (d, *J* = 7.5 Hz, 1 H), 7.46 (d, *J* = 7.5 Hz, 2 H), 7.40 (t, *J* = 7.5 Hz, 1 H), 7.33–7.22 (m, 6 H), 7.18 (t, *J* = 7.5 Hz, 1 H), 6.90 (d, *J* = 7.5 Hz, 2 H), 6.80 (t, *J* = 7.5 Hz, 1 H), 6.73 (t, *J* = 7.5 Hz, 2 H), 4.98 (dd, *J* = 5.0, 9.0 Hz, 1 H), 2.97–2.90 (m, 1 H), 2.48 (d, *J* = 5.0 Hz, 1 H), 2.46–2.39 (m, 1 H), 2.22–1.97 (m, 3 H), 1.76–1.63 (m, 1 H), 1.59 (d, *J* = 5.0 Hz, 1 H).

^13^C NMR (101 MHz, CDCl_3_): δ = 161.8, 148.1, 138.7, 138.4, 135.0, 131.5 (2 C), 129.3, 129.2, 128.6 (3 C), 127.8 (2 C), 126.9 (2 C), 126.4, 126.0, 125.9, 123.7, 122.7, 122.1, 120.7, 110.5, 110.2, 69.8, 69.7, 53.1, 43.9, 39.1, 26.8, 25.2, 18.0.

HRMS (ESI): *m*/*z* [M+H]^+^ calcd for C_33_H_28_N_3_: 466.2278; found: 466.2285.

**(±)-(1’*R*,1a’*R*,2’*R*,6a’*R*,6b’*S*)-1’-Ethynyl-1a’,6b’-diphenyl-1a’,4’,5’,6’,6a’,6b’-hexahydro-1’*H*-spiro[benzo[4,5]imidazo[1,2-*a*]indole-11,2’-cyclopropa[*a*]pyrrolizine] (4i)**

Cycloadduct **4d** (80 mg, 0.142 mmol) was dissolved in ether (1 mL) and methanol (3 mL). Anhydrous K_2_CO_3_ (20.9 mg; 0.151 mmol) was added and the resulting suspension was stirred at ambient temperature for 42 h in a sealed tube. The precipitate that formed was filtered off and washed with methanol and hexane to afford pure product **4i**.

Yield: 64.9 мг (92%); white solid; mp 259–262 °C; R*_f_* 0.43 (SiO_2_, hexane–ethyl acetate, 3:1).

IR (KBr): 3296, 3056, 2971, 2911, 2814, 1611, 1528, 1494, 1473, 1447, 1349, 1224, 1169, 926, 742, 704, 644, 624, 550, 442 cm^−1^.

^1^H NMR (400 MHz, CDCl_3_): δ = 7.92–7.86 (m, 2 H), 7.74 (d, *J* = 7.8 Hz, 2 H), 7.52–7.42 (m, 2 H), 7.40–7.20 (m, 7 H), 6.96 (d, *J* = 7.8 Hz, 2 H), 6.87 (t, *J* = 7.8 Hz, 1 H), 6.78 (t, *J* = 7.8 Hz, 2 H), 4.82 (m, 1 H), 3.42 (s, 1 H), 2.88 (m, 1 H), 2.40 (m, 1 H), 2.22–2.00 (m, 4 H), 1.78 (m, 1 H).

^13^C NMR (101 MHz, CDCl_3_): δ = 160.8, 147.9, 138.7, 134.0, 132.0 (2 C), 130.7, 130.2 (2 C), 129.6, 129.1, 127.5 (3 C), 126.9, 126.6 (2 C), 126.5, 125.8, 124.0, 122.9, 122.2, 120.6, 110.6, 110.2, 81.5, 70.0, 71.2, 69.6, 56.1, 43.4, 42.9, 26.5, 25.1, 17.5.

HRMS (ESI): *m*/*z* [M+H]^+^ calcd for C_35_H_28_N_3_: 489.2205; found: 489.2207.

**General Procedure B for the One-Pot Three-Component Reaction of 11*H*-Benzo[4,5]imidazo[1,2-*a*]indol-11-one, Primary *α*-Amino Acids and Cyclopropenes**

11*H*-Benzo[4,5]imidazo[1,2-a]indol-11-one (**2**, 0.3 mmol), primary *α*-amino acid (**3**, 0.6 mmol), cyclopropene (**1**, 0.3 mmol), and anhydrous 1,4-dioxane (4 mL) were added to a screw-capped tube (Schuett-biotec type). The reaction vessel was closed, and the reaction mixture was stirred in a preheated oil bath at 100 °C for 18 h (the reaction progress was monitored by TLC). The mixture was cooled to room temperature, and the solvent was removed under reduced pressure. The residue was subjected to silica gel PTLC using a mixture of hexane̶̶–ethyl acetate as an eluent to give the spiro-adducts **6a**−**j**.

**(±)-(1’*R*,2’*R*,4’*R*,5’*S*,6’*R*)-4’-Ethyl-1’,5’,6’-triphenyl-3’-azaspiro[benzo[4,5]imidazo[1,2-*a*]indole-11,2’-bicyclo[3.1.0]hexane] (6a)**

Prepared according to the general procedure **B** using cyclopropene **1a** (91.5 mg, 0.34 mmol), ketone **2** (75.0 mg, 0.34 mmol), and *DL*-2-aminobutanoic acid (**3b**) (70.1 mg, 0.68 mmol). Purification of the crude by PTLC (hexane–ethyl acetate, 3:1) furnished corresponding cycloadduct (82 mg, 46%) as an inseparable mixture of diastereomers in a ratio of 2.6:1, respectively. Crystallization of the mixture from methanol gave the major diastereomer **6a** as a pure compound (35 mg).

Data for **6a**: beige solid; mp 236–238 °C; R*_f_* 0.51 (SiO_2_, hexane–ethyl acetate, 3:1).

IR (KBr): 3057, 2962, 2874, 1606, 1527, 1494, 1470, 1445, 1343, 1220, 1166, 908, 732, 699, 550, 438 cm^−1^.

^1^H NMR (400 MHz, CDCl_3_): δ = 8.00 (d, *J* = 7.8 Hz, 1 H), 7.98 (br.d, *J* = 7.8 Hz, 1 H), 7.63 (d, *J* = 7.5 Hz, 2 H), 7.47 (d, *J* = 7.8 Hz, 1 H), 7.40–7.20 (m, 8 H), 7.02 (t, *J* = 7.5 Hz, 1 H), 6.93 (t, *J* = 7.5 Hz, 2 H), 6.90–6.50 (br.s, 2 H), 6.73 (t, *J* = 7.5 Hz, 1 H), 6.59 (br.t, *J* = 7.5 Hz, 2 H), 6.47 (d, *J* = 7.5 Hz, 2 H), 4.67 (dd, 4.0, 9.0 Hz, 1 H), 3.85 (s, 1 H), 2.70–2.00 (br.s, 1 H), 1.76–1.60 (m, 2 H), 0.94 (br.t, *J* = 7.0 Hz, 3 H).

^13^C NMR (101 MHz, CDCl_3_): δ = 163.8, 148.1, 138.4, 137.0, 136.8, 134.3, 133.1 (2 C), 132.3 (2 C), 131.0, 130.9 (2 C), 129.4, 129.2, 127.6 (2 C), 126.6 (2 C), 126.4 (4 C), 125.3, 125.0, 124.2, 123.0, 122.2, 120.7, 110.3, 110.2, 71.2, 68.5, 52.0, 47.4, 29.3, 23.7, 11.1.

HRMS (ESI): *m*/*z* [M+H]^+^ calcd for C_38_H_32_N_3_: 530.2591; found: 530.2595.

**(±)-(1’*R*,2’*R*,4’*R*,5’*S*,6’*R*)-1’,5’,6’-Triphenyl-4’-propyl-3’-azaspiro[benzo[4,5]imidazo [1,2-*a*]indole-11,2’-bicyclo[3.1.0]hexane] (6b)**

Prepared according to the general procedure **B** using cyclopropene **1a** (91.5 mg, 0.34 mmol), ketone **2** (75.0 mg, 0.34 mmol), and *DL*-norvaline (**3c**) (79.7 mg, 0.68 mmol). Purification of the crude by PTLC (hexane–ethyl acetate, 3:1) furnished the corresponding cycloadduct (92 mg, 50%) as an inseparable mixture in a ratio of 2.1:1, respectively. Crystallization of the mixture from methanol gave the major diastereomer **6b** as a pure compound (41 mg).

Data for **6b**: beige solid; mp 262–264 °C; R*_f_* 0.49 (SiO_2_, hexane–ethyl acetate, 3:1).

IR (KBr): 3372, 3049, 3029, 2955, 2928, 2870, 1608, 1496, 1472, 1446, 1344, 1224, 914, 762, 730, 699, 559, 546 cm^−1^.

^1^H NMR (400 MHz, CDCl_3_): δ = 8.00 (d, *J* = 7.8 Hz, 1 H), 7.97 (d, *J* = 7.8 Hz, 1 H), 7.64 (d, *J* = 7.5 Hz, 2 H), 7.47 (d, *J* = 7.7 Hz, 1 H), 7.42–7.18 (m, 8 H), 7.02 (t, *J* = 7.5 Hz, 1 H), 6.93 (t, *J* = 7.5 Hz, 2 H), 6.80–6.40 (br.s, 2 H), 6.73 (t, *J* = 7.5 Hz, 1 H), 6.59 (br.t, *J* = 7.5 Hz, 2 H), 6.47 (d, *J* = 7.5 Hz, 2 H), 4.73 (dd, 3.4, 9.5 Hz, 1 H), 3.84 (s, 1 H), 2.80–1.80 (br.s, 1 H), 1.72–1.50 (m, 2 H), 1.50–1.36 (m, 1 H), 1.36–1.21 (m, 1 H), 0.89 (t, *J* = 7.3 Hz, 3 H).

^13^C NMR (101 MHz, CDCl_3_): δ = 163.6, 148.1, 138.5, 137.2, 134.4, 133.3 (2 C), 132.4 (2 C), 131.2, 131.0 (3 C), 129.5, 129.3, 127.7 (2 C), 126.7 (2 C), 126.5 (3 C), 125.44, 125.39, 125.2, 124.3, 123.1, 122.3, 120.8, 110.4, 110.3, 71.3, 67.1, 51.9, 47.4, 33.1, 29.3, 20.0, 14.4.

HRMS (ESI): *m*/*z* [M+H]^+^ calcd for C_39_H_34_N_3_: 544.2747; found: 544.2752.

**(±)-(1’*R*,2’*R*,4’*R*,5’*S*,6’*R*)-4’-(2-(Methylthio)ethyl)-1’,5’,6’-triphenyl-3’-azaspiro[benzo[4,5]imidazo[1,2-*a*]indole-11,2’-bicyclo[3.1.0]hexane] (6c)**

Prepared according to the general procedure **B** using cyclopropene **1a** (91.5 mg, 0.34 mmol), ketone **2** (75.0 mg, 0.34 mmol), and *L*-methionine (**3d**) (101.5 mg, 0.68 mmol). Purification of the crude by PTLC (hexane–ethyl acetate, 3:1) furnished the corresponding cycloadduct (96 mg, 49%) as an inseparable mixture in a ratio of 2.3:1, respectively. Crystallization of the mixture from methanol gave the major diastereomer **6c** as a pure compound (57 mg).

Data for **6c**: white solid; mp 245–247 °C; R*_f_* 0.47 (SiO_2_, hexane–ethyl acetate, 3:1).

IR (KBr): 3447, 3309, 3059, 3026, 2915, 2848, 1608, 1521, 1493, 1472, 1445, 1341, 1218, 1169, 1079, 739, 698 cm^−1^.

^1^H NMR (400 MHz, CDCl_3_): δ = 7.99 (d, *J* = 7.8 Hz, 1 H), 7.97 (d, *J* = 7.8 Hz, 1 H), 7.64 (d, *J* = 7.5 Hz, 2 H), 7.47 (d, *J* = 7.7 Hz, 1 H), 7.41–7.21 (m, 8 H), 7.03 (t, *J* = 7.5 Hz, 1 H), 6.94 (t, *J* = 7.5 Hz, 2 H), 6.80–6.40 (br.s, 2 H), 6.74 (t, *J* = 7.5 Hz, 1 H), 6.59 (br.t, *J* = 7.5 Hz, 2 H), 6.47 (d, *J* = 7.5 Hz, 2 H), 4.81 (t, 6.0 Hz, 1 H), 3.87 (s, 1 H), 2.95–2.40 (br.s, 1 H), 2.63–2.43 (m, 2 H), 2.03 (s, 3 H), 2.01–1.90 (m, 2 H).

^13^C NMR (101 MHz, CDCl_3_): δ = 163.3, 148.0, 138.4, 136.7 (2 C), 133.8, 133.1 (2 C), 132.3 (2 C), 130.9 (3 C), 129.5, 129.2, 127.7 (2 C), 126.8, 126.6 (2 C), 126.4 (3 C), 125.3, 125.2, 124.3, 123.0, 122.3, 120.3, 110.4, 110.2, 71.2, 66.7, 51.7, 47.1, 31.5, 30.5, 29.5, 15.5.

HRMS (ESI): *m*/*z* [M+H]^+^ calcd for C_39_H_34_N_3_S: 576.2468; found: 576.2470.

**(±)-(1’*R*,2’*R*,4’*R*,5’*S*,6’*R*)-4’-Butyl-1’,5’,6’-triphenyl-3’-azaspiro[benzo[4,5]imidazo[1,2-*a*]indole-11,2’-bicyclo[3.1.0]hexane] (6d)**

Prepared according to the general procedure **B** using cyclopropene **1a** (91.5 mg, 0.34 mmol), ketone **2** (75.0 mg, 0.34 mmol), and *DL*-norleucine (**3e**) (89.2 mg, 0.68 mmol). Purification of the crude by PTLC (hexane–ethyl acetate, 3:1) furnished the corresponding cycloadduct (79 mg, 42%) as an inseparable mixture in a ratio of 2.4:1, respectively. Crystallization of the mixture from methanol gave the major diastereomer **6d** as a pure compound (25 mg).

Data for **6d**: white solid; mp 267–270 °C; R*_f_* 0.46 (SiO_2_, hexane–ethyl acetate, 3:1).

IR (KBr): 3306, 3031, 2955, 2929, 1608, 1538, 1493, 1473, 1447, 1364, 1226, 1168, 1032, 944, 807, 749, 738, 698, 551 cm^−1^.

^1^H NMR (400 MHz, CDCl_3_): δ = 7.98 (d, *J* = 7.8 Hz, 1 H), 7.92 (d, *J* = 7.8 Hz, 1 H), 7.55 (d, *J* = 7.5 Hz, 1 H), 7.41–7.26 (m, 9 H), 7.20 (d, *J* = 7.5 Hz, 1 H), 6.96 (t, *J* = 7.5 Hz, 1 H), 6.89 (t, *J* = 7.5 Hz, 2 H), 6.85–6.40 (br.s, 2 H), 6.76 (t, *J* = 7.5 Hz, 1 H), 6.62 (br.s, 2 H), 6.53 (d, *J* = 7.5 Hz, 2 H), 4.28 (dd, 3.4, 9.5 Hz, 1 H), 3.83 (s, 1 H), 3.20–2.60 (br.s, 1 H), 1.76–1.60 (m, 2 H), 1.55–1.43 (m, 1 H), 1.33–1.21 (m, 3 H), 0.83 (t, *J* = 7.3 Hz, 3 H).

^13^C NMR (101 MHz, CDCl_3_): δ = 160.8, 147.4, 140.8, 137.2, 136.8, 134.2, 132.6 (2 C), 132.0, 131.7 (2 C), 130.9. (2 C), 129.5, 128.7, 127.9 (2 C), 126.9 (2 C), 126.8, 126.3 (2 C), 126.2, 124.9 (2C), 124.4, 123.4, 122.7, 120.6, 110.6, 110.5, 72.8, 71.0, 53.4, 50.2, 30.7, 30.2, 29.3, 22.8, 13.9.

HRMS (ESI): *m*/*z* [M+H]^+^ calcd for C_40_H_36_N_3_: 558.2904; found: 558.2905.

**(±)-(1’*R*,2’*R*,4’*R*,5’*S*,6’*R*)-4’-Isobutyl-1’,5’,6’-triphenyl-3’-azaspiro[benzo[4,5]imidazo[1,2-*a*]indole-11,2’-bicyclo[3.1.0]hexane] (6e)**

Prepared according to the general procedure **B** using cyclopropene **1a** (91.5 mg, 0.34 mmol), ketone **2** (75.0 mg, 0.34 mmol), and *L*-leucine (**3f**) (89.2 mg, 0.68 mmol). Purification of the crude by PTLC (hexane–ethyl acetate, 3:1) furnished the corresponding cycloadduct (101 mg, 53%) as an inseparable mixture in a ratio of 2:1, respectively. Crystallization of the mixture from methanol gave the major diastereomer **6e** as a pure compound (52 mg).

Data for **6e**: white solid; mp 255–257 °C; R*_f_* 0.66 (SiO_2_, hexane–ethyl acetate, 3:1).

IR (KBr): 3421, 3056, 3028, 2960, 2914, 2871, 2840, 1609, 1496, 1472, 1446, 1341, 1226, 1170, 763, 728, 699, 563, 546 cm^−1^.

^1^H NMR (400 MHz, CDCl_3_): δ = 8.00 (d, *J* = 7.8 Hz, 1 H), 7.97 (d, *J* = 7.8 Hz, 1 H), 7.79 (d, *J* = 7.5 Hz, 2 H), 7.47 (d, *J* = 7.7 Hz, 1 H), 7.41–7.20 (m, 8 H), 7.02 (t, *J* = 7.5 Hz, 1 H), 6.94 (t, *J* = 7.5 Hz, 2 H), 6.85–6.45 (br.s, 2 H), 6.73 (t, *J* = 7.5 Hz, 1 H), 6.59 (t, *J* = 7.5 Hz, 2 H), 6.48 (d, *J* = 7.5 Hz, 2 H), 4.80 (dd, 3.4, 9.5 Hz, 1 H), 3.80 (s, 1 H), 2.80–1.80 (br.s, 1 H), 1.75–1.56 (m, 2 H), 1.42–1.32 (m, 1 H), 0.91 (d, *J* = 6.3 Hz, 3 H), 0.83 (d, *J* = 6.3 Hz, 3 H).

^13^C NMR (101 MHz, CDCl_3_): δ = 163.5, 148.2, 138.5, 137.1, 134.2, 133.1 (2 C), 132.3 (2 C), 131.1, 130.9 (2 C), 129.4 (2 C), 129.2, 127.8, 127.6 (2 C), 126.6 (2 C), 126.4 (3 C), 125.2, 125.0, 124.2, 122.9, 122.1, 120.8, 110.3, 110.2, 71.4, 65.2, 51.8, 47.5, 39.7, 29.2, 25.4, 23.9, 21.7.

HRMS (ESI): *m*/*z* [M+H]^+^ calcd for C_40_H_36_N_3_: 558.2904; found: 558.2909.

**(±)-(1’*R*,2’*R*,4’*R*,5’*S*,6’*R*)-6’-Ethyl-4’-isobutyl-1’,5’-diphenyl-3’-azaspiro[benzo[4,5]imidazo[1,2-*a*]indole-11,2’-bicyclo[3.1.0]hexane] (6f)**

Prepared according to the general procedure **B** using cyclopropene **1b** (70.0 mg, 0.32 mmol), ketone **2** (70.0 mg, 0.32 mmol), and *L*-leucine (**3f**) (83.4 mg, 0.64 mmol). Purification of the crude by PTLC (hexane–ethyl acetate, 3:1) furnished the corresponding cycloadduct (79 mg, 48%) as an inseparable mixture in a ratio of 2.7:1, respectively. Crystallization of the mixture from methanol gave the major diastereomer **6f** as a pure compound (31 mg).

Data for **6f**: white solid; mp 215–216 °C; R*_f_* 0.60 (SiO_2_, hexane–ethyl acetate, 3:1).

IR (KBr): 3357, 3054, 2951, 2869, 1609, 1495, 1472, 1445, 1338, 1224, 1165, 770, 738, 700 cm^−1^.

^1^H NMR (400 MHz, CDCl_3_): δ = 7.98 (d, *J* = 7.5 Hz, 2 H), 7.94–7.83 (m, 2 H), 7.55–7.40 (m, 4 H), 7.39–7.23 (m, 5 H), 6.84–6.73 (m, 3 H), 6.68 (t, *J* = 7.5 Hz, 2 H), 4.63 (dd, *J* = 3.0, 9.0 Hz, 1 H), 2.37 (t, *J* = 6.5 Hz 1 H), 2.32–1.87 (br.s, 1 H), 1.66–1.46 (m, 3 H), 1.46–1.28 (m, 2 H), 1.20 (t, *J* = 7.0 Hz, 3 H), 0.88 (d, *J* = 6.3 Hz, 3 H), 0.83 (d, *J* = 6.3 Hz, 3 H).

^13^C NMR (101 MHz, CDCl_3_): δ = 163.9, 148.3, 138.7, 138.0, 137.6, 133.8, 131.8 (2 C), 130.7 (2 C), 129.5, 129.4, 128.1 (2 C), 126.8 (2 C), 126.5, 126.1, 125.1, 124.3, 122.8, 122.1, 120.9, 110.6, 110.2, 70.3, 64.1, 47.2, 44.7, 40.2, 26.4, 25.4, 23.8, 21.9, 20.2, 14.8.

HRMS (ESI): *m*/*z* [M+H]^+^ calcd for C_36_H_36_N_3_: 510.2904; found: 510.2910.

**(±)-(1’*R*,2’*R*,4’*R*,5’*S*,6’*R*)-4’-Isobutyl-1’,5’-diphenyl-6’-vinyl-3’-azaspiro[benzo[4,5]imidazo[1,2-*a*]indole-11,2’-bicyclo[3.1.0]hexane] (6g)**

Prepared according to the general procedure **B** using cyclopropene **1c** (69.4 mg, 0.32 mmol), ketone **2** (70.0 mg, 0.32 mmol), and *L*-leucine (**3f**) (83.4 mg, 0.64 mmol). Purification of the crude by PTLC (hexane–ethyl acetate, 3:1) furnished the corresponding cycloadduct (81 mg, 50%) as an inseparable mixture in a ratio of 3.1:1, respectively. Crystallization of the mixture from methanol gave the major diastereomer **6g** as a pure compound (29 mg).

Data for **6g**: white solid; mp 250–251 °C; R*_f_* 0.59 (SiO_2_, hexane–ethyl acetate, 3:1).

IR (KBr): 3330, 3057, 2954, 2923, 2866, 1607, 1525, 1491, 1470, 1447, 1341, 1223, 1167, 996, 896, 771, 742, 700, 634 cm^−1^.

^1^H NMR (400 MHz, CDCl_3_): δ = 7.97 (d, *J* = 7.5 Hz, 1 H), 7.94 (d, *J* = 7.5 Hz, 2 H), 7.88 (d, *J* = 7.5 Hz, 1 H), 7.48 (d, *J* = 7.5 Hz, 1 H), 7.44–7.36 (m, 3 H), 7.35–7.23 (m, 5 H), 6.93 (d, *J* = 7.5 Hz, 2 H), 6.78 (t, *J* = 7.5 Hz, 1 H), 6.70 (t, *J* = 7.5 Hz, 2 H), 5.49 (dd, *J* = 17.0, 2.5 Hz, 1 H), 5.13 (dt, *J* = 17.0, 10.0 Hz, 1 H), 5.04 (dd, *J* = 10.0, 2.5 Hz, 1 H), 4.74 (dd, *J* = 3.0, 10.0 Hz, 1 H), 3.33 (d, *J* = 10.0 Hz 1 H), 2.50–1.90 (br.s, 1 H), 1.66–1.53 (m, 2 H), 1.42–1.32 (m, 1 H), 0.90 (d, *J* = 6.3 Hz, 3 H), 0.84 (d, *J* = 6.3 Hz, 3 H).

^13^C NMR (101 MHz, CDCl_3_): δ = 163.5, 148.3, 138.4, 138.2, 136.81, 136.82, 132.5, 132.1 (2 C), 132.0 (2 C), 129.4, 129.3, 127.9 (2 C), 126.7 (2 C), 126.6, 126.4, 125.3, 124.2, 122.9, 122.1, 120.7, 113.1, 110.3, 110.2, 70.8, 64.2, 49.5, 46.1, 39.9, 28.5, 25.4, 23.8, 21.8.

HRMS (ESI): *m*/*z* [M+H]^+^ calcd for C_36_H_34_N_3_: 508.2747; found: 508.2755.

**(±)-(1’*R*,2’*R*,4’*R*,5’*S*,6’*R*)-4’-Isobutyl-1’,5’-diphenyl-6’-((trimethylsilyl)ethynyl)-3’-azaspiro[benzo[4,5]imidazo[1,2-*a*]indole-11,2’-bicyclo[3.1.0]hexane] (6h)**

Prepared according to the general procedure **B** using cyclopropene **1d** (91.7 mg, 0.32 mmol), ketone **2** (70.0 mg, 0.32 mmol), and *L*-leucine (**3f**) (83.4 mg, 0.64 mmol). Purification of the crude by PTLC (hexane–ethyl acetate, 3:1) furnished the corresponding cycloadduct (93 mg, 50%) as an inseparable mixture in a ratio of 2.2:1, respectively. Crystallization of the mixture from methanol gave the major diastereomer **6h** as a pure compound (42 mg).

Data for **6h**: white solid; mp 182–185 °C; R*_f_* 0.62 (SiO_2_, hexane–ethyl acetate, 3:1).

IR (KBr): 3362, 3050, 2956, 2919, 2161, 1608, 1526, 1492, 1471, 1467, 1343, 1251, 1224, 1168, 844, 730, 702, 696, 577, 474 cm^−1^.

^1^H NMR (400 MHz, CDCl_3_): δ = 8.13 (d, *J* = 7.5 Hz, 2 H), 7.95 (d, *J* = 7.5 Hz, 1 H), 7.87 (d, *J* = 7.5 Hz, 1 H), 7.50 (d, *J* = 7.5 Hz, 1 H), 7.45–7.36 (m, 3 H), 7.34–7.24 (m, 5 H), 6.95 (d, *J* = 7.5 Hz, 2 H), 6.78 (t, *J* = 7.5 Hz, 1 H), 6.70 (t, *J* = 7.5 Hz, 2 H), 4.80 (dd, *J* = 3.0, 9.0 Hz, 1 H), 3.34 (s, 1 H), 2.45–1.87 (br.s, 1 H), 1.62–1.71 (m, 2 H), 1.49–1.39 (m, 1 H), 0.89 (d, *J* = 6.3 Hz, 3 H), 0.85 (d, *J* = 6.3 Hz, 3 H), -0.1 (s, 9 H).

^13^C NMR (101 MHz, CDCl_3_): δ = 163.1, 148.1, 138.4, 136.2, 135.1, 131.9 (2 C), 131.6, 131.2 (2 C), 129.5, 129.3, 127.5 (2 C), 126.7, 126.4 (3 C), 125.4, 124.3, 123.0, 122.2, 120.8, 110.4, 110.3, 105.0, 92.0, 69.9, 62.7, 50.9, 46.5, 40.2, 25.4, 23.7, 21.8, 15.7, −0.6 (3 C).

HRMS (ESI): *m*/*z* [M+H]^+^ calcd for C_39_H_40_N_3_Si: 578.2986; found: 578.2994.

**(±)-(1’*R*,2’*R*,4’*R*,5’*S*,6’*R*)-4’-Isobutyl-*N*-isopropyl-1’,5’-diphenyl-3’-azaspiro[benzo[4,5]imidazo[1,2-*a*]indole-11,2’-bicyclo[3.1.0]hexane]-6’-carboxamide (6i)**

Prepared according to the general procedure **B** using cyclopropene **1f** (100.8 mg, 0.36 mmol), ketone **2** (80.0 mg, 0.36 mmol), and *L*-leucine (**3f**) (95.3 mg, 0.73 mmol). Purification of the crude by PTLC (hexane–ethyl acetate, 2:1) furnished the corresponding cycloadduct (112 mg, 55%) as an inseparable mixture in a ratio of 1.9:1, respectively. Crystallization of the mixture from methanol gave the major diastereomer **6i** with an admixture of the minor **7i** (overall 51 mg).

Data for **6i**: white solid; mp 214–216 °C; R*_f_* 0.77 (SiO_2_, hexane–ethyl acetate, 2:1).

IR (KBr): 3422, 3297, 3061, 3025, 2955, 2924, 2868, 1635, 1607, 1535, 1494, 1445, 1348, 1219, 1167, 763, 742, 698 cm^−1^.

^1^H NMR (400 MHz, CDCl_3_): δ = 8.00 (d, *J* = 7.7 Hz, 1 H), 7.94 (m, 3 H), 7.72 (d, *J* = 7.7 Hz, 1 H), 7.55–7.40 (m, 5 H), 7.38–7.25 (m, 4 H), 6.98 (d, *J* = 7.7 Hz, 2 H), 6.84 (t, *J* = 7.7 Hz, 1 H), 6.74 (t, *J* = 7.7 Hz, 2 H), 4.63 (d, *J* = 7.0 Hz, 1 H), 3.71 (m, 1 H), 3.54 (br.s, 1 H), 3.37 (s, 1 H), 2.32 (br.s, 1 H), 1.60 (m, 1 H), 0.88 (d, *J* = 6.5 Hz, 3 H), 0.80 (d, *J* = 6.5 Hz, 3 H), 0.59 (d, *J* = 6.5 Hz, 3 H), 0.29 (d, *J* = 6.5 Hz, 3 H).

^13^C NMR (101 MHz, CDCl_3_): δ = 168.2, 148.1, 138.5, 133.3, 132.6 (2 C), 131.4 (2 C), 131.0, 129.9, 129.8, 129.4, 129.1, 128.9, 128.0 (2 C), 127.6, 127.4, 127.3 (2 C), 125.6, 124.6, 123.0, 122.2, 120.8, 110.3, 110.2, 71.2, 65.2, 50.5, 48.0, 40.8, 39.3, 29.2, 25.3, 23.7, 21.6 (2 C).

HRMS (ESI): m/z [M+Na]^+^ calcd for C_38_H_38_N_4_ONa: 589.2938; found: 589.2938.

**(±)-(1’*R*,2’*R*,4’*R*,5’*S*)-4’-Isobutyl-1’,5’-diphenyl-3’-azaspiro[benzo[4,5]imidazo[1,2-*a*]indole-11,2’-bicyclo[3.1.0]hexane] (6j)**

Prepared according to the general procedure **B** using cyclopropene **1h** (61.1 mg, 0.32 mmol), ketone **2** (70.0 mg, 0.32 mmol), and *L*-leucine (**3f**) (83.4 mg, 0.64 mmol). Purification of the crude by PTLC (hexane–ethyl acetate, 3:1) furnished the corresponding cycloadduct (81 mg, 53%) as an inseparable mixture in a ratio of 2.1:1, respectively. Crystallization of the mixture from methanol gave the major diastereomer **6j** as a pure compound (36 mg).

Data for **6j**: white solid; mp 180–183 °C; R*_f_* 0.54 (SiO_2_, hexane–ethyl acetate, 3:1).

IR (KBr): 3364, 3055, 3023, 2952, 2929, 2862, 2836, 1610, 1529, 1492, 1471, 1447, 1337, 1220, 1169, 1048, 1012, 917, 781, 736, 703, 593, 495 cm^−1^.

^1^H NMR (400 MHz, CDCl_3_): δ = 7.96 (d, *J* = 7.5 Hz, 1 H), 7.85 (d, *J* = 7.5 Hz, 1 H), 7.67 (d, *J* = 7.5 Hz, 2 H), 7.51 (d, *J* = 7.5 Hz, 1 H), 7.42–7.23 (m, 7 H), 7.20 (t, *J* = 7.5 Hz, 1 H), 6.87 (d, *J* = 7.5 Hz, 2 H), 6.76 (t, *J* = 7.5 Hz, 1 H), 6.68 (t, *J* = 7.5 Hz, 2 H), 5.03–4.98 (m, 1 H), 3.51 (s, 2 H), 2.41 (d, *J* = 4.0 Hz 1 H), 1.68–1.40 (m, 3 H), 0.92 (d, *J* = 6.3 Hz, 3 H), 0.90 (d, *J* = 6.3 Hz, 3 H).

^13^C NMR (101 MHz, CDCl_3_): δ = 163.6, 148.2, 138.3, 137.8, 137.1, 135.2, 131.3 (2 C), 129.7 (2 C), 129.4, 129.2, 128.0 (2 C), 126.9 (2 C), 126.3, 125.0, 124.1, 122.9, 122.1, 120.8, 110.4, 110.2, 69.9, 60.9, 50.8, 46.9, 41.6, 40.9, 25.5, 23.7, 22.1, 14.4.

HRMS (ESI): *m*/*z* [M+H]^+^ calcd for C_34_H_32_N_3_: 482.2591; found: 482.2592.

**General Procedure C for the One-Pot Three-Component Reaction of 11*H*-Benzo[4,5]imidazo[1,2-*a*]indol-11-one, *L*-Proline and Maleimides**

11*H*-Benzo[4,5]imidazo[1,2-*a*]indol-11-one (**2**, 0.25 mmol), *L*-proline (**3a**, 0.5 mmol), maleimide (**7**, 0.25 mmol), and anhydrous acetonitrile (4 mL) were added to a screw-capped tube (Schuett-biotec type). The reaction vessel was closed and the reaction mixture was stirred in a preheated oil bath at 100 °C for 18 h (the reaction progress was monitored using TLC). The mixture was cooled to room temperature, and the solvent was removed under reduced pressure. The residue was subjected to silica gel PTLC using a mixture of hexane−ethyl acetate as an eluent to give the mixtures of spiro-adducts **8a**−**f** and their minor diastereomers.

**(±)-(3a’*S*,4’*R*,8a’*R*,8b’*R*)-3a’,6’,7’,8’,8a’,8b’-Hexahydro-1’*H*-spiro[benzo[4,5]imidazo[1,2-a]indole-11,4’-pyrrolo[3,4-a]pyrrolizine]-1’,3’(2’H)-dione (8a) and (3a’S,4’S,8a’R,8b’R)-3a’,6’,7’,8’,8a’,8b’-hexahydro-1’H-spiro[benzo[4,5]imidazo[1,2-a]indole-11,4’-pyrrolo[3,4-a]pyrrolizine]-1’,3’(2’H)-dione (8a-minor).**

Prepared according to the general procedure **C** using maleimide **7a** (52.5 mg, 0.54 mmol), ketone **2** (85.0 mg, 0.39 mmol), and *L*-proline (**3a**) (88.9 mg, 0.77 mmol). Purification of the crude by PTLC (hexane-EtOAc, 1:2) furnished **8a** as an inseparable mixture with its minor diastereomer in a ratio of 1.5:1, respectively (113 mg, 78%).

Data for mixture **8a**+**minor**: white solid; mp 244–245 °C.

IR (KBr): 3470, 3152, 3058, 2966, 2765, 1770, 1717, 1609, 1530, 1495, 1474, 1450, 1337, 1194, 743 cm^−1^.

1H NMR (400 MHz, DMSO-*d*_6_): δ = 11.52(**8a**) (br.s, 1 H), 11.03(**minor**) (br.s, 1 H), 8.12(**8a**) (d, *J* = 7.9 Hz, 1 H), 8.09(**minor**) (d, *J* = 7.9 Hz, 1 H), 7.94(**minor**) (d, *J* = 7.9 Hz, 1 H), 7.91(**8a**) (d, *J* = 7.9 Hz, 1 H), 7.84(**minor**) (d, *J* = 7.9 Hz, 1 H), 7.77(**8a**) (d, *J* = 7.9 Hz, 1 H), 7.73(**minor**) (d, *J* = 7.9 Hz, 1 H), 7.59(**minor**) (t, *J* = 7.9 Hz, 1 H), 7.54(**8a**) (t, *J* = 7.9 Hz, 1 H), 7.44–7.19(**8a**+**minor**) (m, 4 H(**8a**) + 3 H(**minor**)), 4.49(**minor**) (td, *J* = 7.0, 2.5 Hz, 1 H), 4.39(**minor**) (d, *J* = 9.8 Hz, 1 H), 4.20(**8a**) (q, *J* = 7.5 Hz, 1 H), 4.08(**minor**) (q, *J* = 5.5 Hz, 1 H), 3.78(**8a**) (d, *J* = 7.5 Hz, 1 H), 3.59(**8a**) (t, *J* = 7.5 Hz, 1 H), 3.56(**minor**) (dd, *J* = 10.6, 7.5 Hz, 1 H), 3.17(8**a**+**minor**) (d, *J* = 5.5 Hz, 1 H), 2.98(**minor**) (m, 1 H), 2.57(**minor**) (t, *J* = 7.5 Hz, 1 H), 2.27(**8a**) (t, *J* = 7.5 Hz, 1 H), 2.17(**minor**) (m, 1 H), 2.05–1.60(**8a**+**minor**) (m, 4 H(**8a**) +1 H(**epimer**)).

^13^C NMR (101 MHz, DMSO-*d*_6_) for **8a**: δ = 177.7, 176.1, 160.6, 147.2, 137.7, 132.4, 130.1, 128.8, 127.3, 124.1, 123.7, 122.8, 120.2, 111.1, 65.1, 64.7, 59.8, 53.9, 44.9, 41.5, 26.0, 22.4.

^13^C NMR (101 MHz, DMSO-*d*_6_) for **8a**-**minor**: δ = 179.2, 176.6, 161.0, 147.0, 138.0, 134.3, 130.4, 128.5, 126.9, 124.6, 123.6, 122.6, 120.2, 111.4, 111.3, 69.0, 65.8, 55.1, 48.6, 48.1, 28.9, 24.6.

HRMS (ESI): *m*/*z* [M+H]^+^ calcd for C_22_H_19_N_4_O_2_: 371.1503; found: 371.1505.

**(±)-(3a’*S*,4’*R*,8a’*R*,8b’*R*)-2’-Methyl-3a’,6’,7’,8’,8a’,8b’-hexahydro-1’*H*-spiro[benzo[4,5]imidazo[1,2-a]indole-11,4’-pyrrolo[3,4-a]pyrrolizine]-1’,3’(2’H)-dione (8b) and (±)-(3a’S,4’S,8a’R,8b’R)-2’-methyl-3a’,6’,7’,8’,8a’,8b’-hexahydro-1’H-spiro[benzo[4,5]imidazo[1,2-a]indole-11,4’-pyrrolo[3,4-a]pyrrolizine]-1’,3’(2’H)-dione (8b-minor)**

Prepared according to the general procedure **C** using maleimide **7b** (57.2 mg, 0.51 mmol), ketone **2** (85.0 mg, 0.39 mmol), and *L*-proline (**3a**) (88.9 mg, 0.77 mmol). Purification of the crude by PTLC (hexane–ethyl acetate, 3:1) furnished **8b** as an inseparable mixture with its minor diastereomer in a ratio of 1.7:1, respectively (104 mg, 69%). Crystallization of the mixture from methanol gave the major diastereomer **8b** as a pure compound (50 mg).

Data for **8b**: white solid; mp 238–239 °C; R*_f_* 0.52 (SiO_2_, hexane–ethyl acetate, 1:2).

IR (KBr): 3064, 2988, 2961, 2949, 2917, 2831, 1774, 1700, 1607, 1471, 1433, 1289, 1222, 1131, 764, 739, 450 cm^−1^.

^1^H NMR (400 MHz, CDCl_3_): δ = 7.86 (d, *J* = 7.9 Hz, 1 H), 7.78 (d, *J* = 7.9 Hz, 1 H), 7.57 (d, *J* = 7.9 Hz, 1 H), 7.51 (t, *J* = 7.9 Hz, 1 H), 7.43 (t, *J* = 7.9 Hz, 1 H), 7.37 (t, *J* = 7.9 Hz, 1 H), 7.25 (t, *J* = 7.9 Hz, 1 H), 7.14 (d, *J* = 7.9 Hz, 1 H), 4.38 (q, *J* = 6.8 Hz, 1 H), 3.92 (d, *J* = 7.5 Hz, 1 H), 3.63 (t, *J* = 7.5 Hz, 1 H), 3.14 (s, 3 H), 2.37 (m, 1 H), 2.14–1.90 (m, 5 H).

^13^C NMR (101 MHz, CDCl_3_): δ = 176.0, 175.0, 160.4, 147.4, 138.3, 132.3, 130.1, 129.2, 127.3, 124.2, 123.9, 123.0, 120.6, 110.8, 110.7, 65.9, 65.3, 58.8, 43.9, 41.5, 26.3, 25.0, 22.6.

HRMS (ESI): m/z [M+H]^+^ calcd for C_23_H_21_N_4_O_2_: 385.1659; found: 385.1662.

**(±)-(3a’*S*,4’*R*,8a’*R*,8b’*R*)-2’-Phenyl-3a’,6’,7’,8’,8a’,8b’-hexahydro-1’*H*-spiro[benzo[4,5]imidazo[1,2-a]indole-11,4’-pyrrolo[3,4-a]pyrrolizine]-1’,3’(2’H)-dione (8c) and (±)-(3a’S,4’S,8a’R,8b’R)-2’-phenyl-3a’,6’,7’,8’,8a’,8b’-hexahydro-1’H-spiro[benzo[4,5]imidazo[1,2-a]indole-11,4’-pyrrolo[3,4-a]pyrrolizine]-1’,3’(2’H)-dione (8c-minor)**

Prepared according to the general procedure **C** using maleimide **7c** (42.5 mg, 0.23 mmol), ketone **2** (54.0 mg, 0.25 mmol), and *L*-proline (**3a**) (56.5 mg, 0.49 mmol). Purification of the crude by PTLC (hexane-EtOAc, 1:2) furnished **8c** as an inseparable mixture with its minor diastereomer in a ratio of 9:1, respectively (68.2 mg, 61%).

Data for mixture **8c**+**minor**: light pink color; mp 198–200 °C.

IR (KBr): 3054, 2961, 2924, 2829, 1777, 1709, 1609, 1525, 1494, 1472, 1390, 1371, 1221, 1185, 744, 692, 616 cm^−1^.

^1^H NMR (400 MHz, CDCl_3_): δ = 7.89(**8c**) (d, *J* = 7.9 Hz, 1 H), 7.79(**8c**) (d, *J* = 7.9 Hz, 1 H), 7.60–7.35(**8c**+**minor**) (m, 9 H), 7.28(**8c**) (d, *J* = 7.9 Hz, 1 H), 7.22(**8c**) (t, *J* = 7.9 Hz, 1 H), 4.52(**8c**) (q, *J* = 6.5 Hz, 1 H), 4.36(**minor**) (br.s, 1 H), 4.16(**minor**) (d, *J* = 8.5 Hz, 1 H), 4.08 (**8c**) (d, *J* = 7.5 Hz, 1 H), 4.00(**minor**) (br.s, 1 H), 3.81(**8c**) (t, *J* = 7.5 Hz, 1 H), 2.77(**minor**) (br.m, 1 H), 2.48(**8c**+**minor**) (m, 1 H), 2.18–1.98(**8c**+**minor**) (m, 5 H).

^13^C NMR (101 MHz, CDCl_3_) for **8c**: δ = 175.1, 173.7, 160.5, 138.4, 132.2, 131.8, 130.1, 129.1 (2 C), 128.6, 127.5, 126.3 (2 C), 124.3, 124.0, 123.0, 120.6, 110.9, 110.7, 66.3, 65.9, 58.7, 43.9, 42.0, 26.4, 22.8.

HRMS (ESI): *m*/*z* [M+H]^+^ calcd for C_28_H_23_N_4_O_2_: 447.1816; found: 447.1820.

**(±)-(3a’*S*,4’*R*,8a’*R*,8b’*R*)-2’-Benzyl-3a’,6’,7’,8’,8a’,8b’-hexahydro-1’*H*-spiro[benzo[4,5]imidazo[1,2-a]indole-11,4’-pyrrolo[3,4-a]pyrrolizine]-1’,3’(2’H)-dione (8d) and (±)-(3a’S,4’S,8a’R,8b’R)-2’-benzyl-3a’,6’,7’,8’,8a’,8b’-hexahydro-1’H-spiro[benzo[4,5]imidazo[1,2-a]indole-11,4’-pyrrolo[3,4-a]pyrrolizine]-1’,3’(2’H)-dione (8d-minor)**

Prepared according to the general procedure **C** using maleimide **7d** (45.9 mg, 0.25 mmol), ketone **2** (54.0 mg, 0.25 mmol), and *L*-proline (**3a**) (56.5 mg, 0.49 mmol). Purification of the crude by PTLC (hexane-EtOAc, 1:2) furnished **8d** as an inseparable mixture with its minor diastereomer in a ratio of 2.3:1, respectively (103 mg, 89%).

Data for mixture **8d**+**minor**: white solid; mp 164–166 °C.

IR (KBr): 3062, 2961, 2829, 1770, 1702, 1698, 1608, 1494, 1474, 1398, 1343, 1222, 1173, 757, 737, 709, 618 cm^−1^.

^1^H NMR (400 MHz, CDCl_3_): δ = 7.85(**8d**) (d, *J* = 7.9 Hz, 1 H), 7.75(**8d**) (d, *J* = 7.9 Hz, 1 H), 7.70(**minor**) (d, *J* = 7.9 Hz, 1 H), 7.67(**minor**) (d, *J* = 7.9 Hz, 1 H), 7.62(**minor**) (br.d, *J* = 7.9 Hz, 1 H), 7.56(**minor**) (t, *J* = 7.9 Hz, 1 H), 7.52(**8d**+**minor**) (t, *J* = 7.5 Hz, 2 H), 7.43(**8d**+**minor**) (t, *J* = 7.5 Hz, 1 H), 7.52(**8d**+**minor**) (d, *J* = 7.5 Hz, 2 H), 7.38–7.22(**8d**+**minor**) (m, 4 H), 6.97(**8d**) (t, *J* = 7.9 Hz, 1 H), 6.55(**8d**) (d, *J* = 7.9 Hz, 1 H), 4.87(**8d**) (d, *J* = 13.6 Hz, 1 H), 4.75(**minor**) (br.m, 1 H), 4.68(**8d**) (d, *J* = 13.6 Hz, 1 H), 4.66(**minor**) (d, *J* = 14.3 Hz, 1 H), 4.43(**8d**) (q, *J* = 6.8 Hz, 1 H), 4.38(**minor**) (d, *J* = 14.3 Hz, 1 H), 4.23(**minor**) (br.d, *J* = 9.6 Hz, 1 H), 3.87 (**8d**) (d, *J* = 7.5 Hz, 1 H), 3.66(**8d**) (t, *J* = 7.5 Hz, 1 H), 3.48(**minor**) (m, 1 H), 2.87(**minor**) (q, *J* = 7.5 Hz, 1 H), 2.76(**minor**) (br.t, *J* = 7.5 Hz, 1 H), 2.39(**minor**) (m, 1 H), 2.27(**8d**) (m, 1 H), 2.15–1.90(**8d**+ **minor**) (m, 5 H(**8d**) +3 H(**minor**)).

^13^C NMR (101 MHz, CDCl_3_) for **8d**: δ = 175.7, 174.4, 160.5, 147.4, 138.2, 135.6, 132.1, 129.1 (2 C), 128.8, 128.5 (2 C), 128.3, 128.0, 127.8, 124.0, 123.9, 122.9, 120.6, 110.7, 110.6, 66.0, 65.7, 58.4, 44.1, 42.6, 42.0, 26.2, 22.7.

HRMS (ESI): m/z [M+H]^+^ calcd for C_29_H_25_N_4_O_2_: 461.1972; found: 461.1978.

**(±)-(3a’*S*,4’*R*,8a’*R*,8b’*R*)-2’-Phenethyl-3a’,6’,7’,8’,8a’,8b’-hexahydro-1’*H*-spiro[benzo[4,5]imidazo[1,2-a]indole-11,4’-pyrrolo[3,4-a]pyrrolizine]-1’,3’(2’H)-dione (8e) and (±)-(3a’S,4’S,8a’R,8b’R)-2’-phenethyl-3a’,6’,7’,8’,8a’,8b’-hexahydro-1’H-spiro[benzo[4,5]imidazo[1,2-a]indole-11,4’-pyrrolo[3,4-a]pyrrolizine]-1’,3’(2’H)-dione (8e-minor)**

Prepared according to the general procedure **C** using maleimide **7e** (49.3 mg, 0.25 mmol), ketone **2** (54.0 mg, 0.25 mmol), and *L*-proline (**3a**) (56.5 mg, 0.49 mmol). Purification of the crude by PTLC (hexane-EtOAc, 1:2) furnished **8e** as an inseparable mixture with its minor diastereomer in a ratio of 1.5:1, respectively (95.4 mg, 80%).

Data for mixture **8e**+**minor**: white solid; mp 162–164 °C.

IR (KBr): 3060, 3027, 2956, 2930, 2817, 1772, 1702, 1609, 1496, 1473, 1449, 1400, 1363, 1168, 767, 747, 738, 698 cm^−1^.

^1^H NMR (400 MHz, CDCl_3_): δ = 7.86(**8e**) (d, *J* = 7.9 Hz, 1 H), 7.77(**8e**) (d, *J* = 7.9 Hz, 1 H), 7.72(**minor**) (d, *J* = 7.9 Hz, 1 H), 7.70(**minor**) (d, *J* = 7.9 Hz, 1 H), 7.64(**minor**) (br.d, *J* = 7.9 Hz, 1 H), 7.60–7.52(**8e**+**minor**) (m, 1 H(**8e**) + 2 H(**minor**)), 7.48(**8e**) (t, *J* = 7.5 Hz, 1 H), 7.43(**8e**) (t, *J* = 7.5 Hz, 1 H), 7.39–7.17(**8e**+**minor**) (m, 6 H(**8e**) + 8 H(**minor**)), 7.14(**8e**) (t, *J* = 7.5 Hz, 1 H), 6.73(**8e**) (d, *J* = 7.5 Hz, 1 H), 4.79(**minor**) (br.m, 1 H), 4.40(**8e**) (m, 1 H), 4.20(**8e**) (d, *J* = 9.5 Hz, 1 H), 3.98(**8e**) (dt, *J* = 13.4, 8.0 Hz, 1 H), 3.86(**8e**) (ddd, *J* = 13.4, 6.0, 8.0 Hz, 1 H), 3.8(**8e**) (d, *J* = 7.5 Hz, 1 H), 3.59(**8e**) (m, 2 H), 3.47(**minor**) (m, 1 H), 3.13(**8e**) (dt, *J* = 13.4, 8.0 Hz, 1 H), 3.02(**8e**) (ddd, *J* = 13.4, 6.0, 8.0 Hz, 1 H), 3.0–2.78(**minor**) (m, 3 H), 2.69(**minor**) (m, 1 H), 2.43(**minor**) (m, 1 H), 2.24(**8e**) (m, 1 H), 2.18–1.88(**8e**+**minor**) (m, 4 H(**8e**) + 5 H(**minor**)).

^13^C NMR (101 MHz, CDCl_3_) for **8e**: δ = 176.0, 174.5, 160.4, 147.6, 138.3, 137.6, 131.9, 130.4, 129.9, 129.1 (2 C), 128.4 (2 C), 128.3, 127.7, 126.7, 124.0, 122.9, 120.6, 110.7, 110.6, 65.7, 65.3, 58.3, 43.6, 41.6, 40.3, 33.3, 26.3, 22.8.

HRMS (ESI): *m*/*z* [M+H]^+^ calcd for C_30_H_27_N_4_O_2_: 475.2129; found: 475.2134.

**(±)-(3a’*S*,4’*R*,8a’*R*,8b’*R*)-2’-(2,2-Diphenylethyl)-3a’,6’,7’,8’,8a’,8b’-hexahydro-1’H-spiro[benzo [4,5]imidazo[1,2-a]indole-11,4’-pyrrolo[3,4-a]pyrrolizine]-1’,3’(2’H)-dione (8f) and (±)-(3a’S,4’S,8a’R,8b’R)-2’-(2,2-diphenylethyl)-3a’,6’,7’,8’,8a’,8b’-hexahydro-1’H-spiro[benzo[4,5]imidazo[1,2-a]indole-11,4’-pyrrolo[3,4-*a*]pyrrolizine]-1’,3’(2’*H*)-dione (8f-minor)**

Prepared according to the general procedure **C** using maleimide **7f** (69.3 mg, 0.25 mmol), ketone **2** (54.0 mg, 0.25 mmol), and *L*-proline (**3a**) (56.5 mg, 0.49 mmol). Purification of the crude by PTLC (hexane-EtOAc, 1:2) furnished **8f** as an inseparable mixture with its minor diastereomer in a ratio of 1.2:1, respectively (93.8 mg, 68%).

Data for mixture **8f**+**minor**: white solid; mp 219–221 °C.

IR (KBr): 3053, 3026, 2960, 2945, 2878, 2828, 1773, 1700, 1609, 1492, 1472, 1446, 1398, 1345, 1169, 756, 744, 701 cm^−1^.

^1^H NMR (400 MHz, CDCl_3_) for **8f**+**minor**: δ = 7.84(**minor**) (d, *J* = 7.9 Hz, 1 H), 7.76(**8f**) (d, *J* = 7.9 Hz, 1 H), 7.74(**8f**) (d, *J* = 7.9 Hz, 1 H), 7.70(**minor**) (d, *J* = 7.9 Hz, 1 H), 7.60–7.46(**8f**+**minor**) (m, 3 H), 7.44–7.32(**8f**+**minor**) (m, 11 H), 7.31–7.10(**8f**+**minor**) (m, 14 H), 7.52(**minor**) (d, *J* = 7.9 Hz, 1 H), 4.83(**8f**) (t, *J* = 8.3 Hz, 1 H), 4.69(**minor**) (br.s, 1 H), 4.59(**minor**) (t, *J* = 8.3 Hz, 1 H), 4.38(**8f**) (br.q, *J* = 7.5 Hz, 1 H), 4.32(**8f**) (dd, *J* = 8.9, 13.5 Hz, 1 H), 4.24(**8f**) (dd, *J* = 7.7, 13.5 Hz, 1 H), 4.15(**minor**) (dd, *J* = 8.2, 13.2 Hz, 1 H), 4.03(**minor**) (d, *J* = 9.1 Hz, 1 H), 3.85(**minor**) (dd, *J* = 9.1, 13.2 Hz, 1 H), 3.67(**8f**) (d, *J* = 7.5 Hz, 1 H), 3.53(**minor**) (t, *J* = 7.5 Hz, 1 H), 3.29(**8f**) (br.t, *J* = 8.0 Hz, 1 H), 2.88–2.73(**8f**+**minor**) (m, 2 H), 2.28(**8f**) (br.q, *J* = 8.5 Hz, 1 H), 2.18 (br.s, 1 H), 2.11–1.80(**8f**+**minor**) (m, 8 H).

^13^C NMR (101 MHz, CDCl_3_) for **8f**+**minor**: δ = 177.1(**8f**), 176.4(**minor**), 174.6(**8f**), 174.5(**minor**), 160.6(**8f**), 150.1(**minor**), 147.8(**8f**), 147.5(**minor**), 141.13(**minor**), 141.07(**8f**), 141.05(**minor**), 140.8(**8f**), 139.0(**minor**), 138.3(**8f**), 131.6(**8f**+**minor**), 130.3(**minor**), 129.9(**8f**), 129.2(**8f**), 129.1(**minor**), 128.48(**8f**) (2 C), 128.47(**minor**) (2 C), 128.43(**8f**) (2 C), 128.35(**minor**) (2 C), 128.3(**8f**) (2 C), 128.1(**8f**), 128.04(**minor**) (3 C), 128.02(**8f**) (2 C), 127.96(**minor**) (2 C), 127.0(**minor**), 126.8(**8f**), 126.6(**8f**+**minor**), 126.0(**minor**), 124.2(**8f**), 123.9(**minor**), 123.8(**8f**), 123.6(**8f**), 122.9(**8f**), 122.6(**minor**), 120.9(**minor**), 120.6(**8f**), 111.2(**minor**), 110.6(**8f**), 110.53(**minor**), 110.49(**8f**), 68.9(**minor**), 66.5(**8f**), 65.5(**8f**), 57.7(**8f**+**minor**), 54.4(**8f**), 51.9(**minor**), 48.1(**minor**), 47.77(**minor**), 47.75(**8f**), 43.79(**minor**), 43.75(**8f**), 43.1(**minor**), 42.0(**8f**+ **minor**), 29.4(**minor**), 26.1(**8f**), 25.3(**minor**), 22.9(**8f**).

HRMS (ESI): m/z [M+H]^+^ calcd for C_36_H_31_N_4_O_2_: 551.2442; found: 551.2450.

### 3.2. Computational Methodology

Full geometry optimization of reactants, products, and transition state structures (TSs) was performed at the DFT/HF level of theory using M11 hybrid exchange-correlation functional [49] and cc-pVDZ basis set [50]. The polarizable continuum model (PCM) was used to calculate solvent effects of 1,4-dioxane [51]. The optimizations were carried out using the Berny analytical gradient optimization method [52]. All stationary points were described by harmonic vibrational frequency calculations to prove the location of correct minima (only real frequencies) and transition states (only one imaginary frequency). For the transition states, the normal modes corresponding to the imaginary frequencies were related to the vibrations of new developing bonds. IRC calculations were performed to check the energy profiles connecting each TS to the two associated minima of the proposed mechanism [53]. Thermal corrections to enthalpy and entropy values were evaluated at 298.15 K and 1.0 atm. All calculations were performed using the Gaussian 09 computational program package [54]. Data on energies (a.u.) and cartesian coordinates of stationary points for reactants, intermediates, products and the transition states (M062x/cc-pVDZ, PCM = 1,4-dioxane) are given at Supporting Information, (Table S5).

### 3.3. Cell Culture and Culturing Conditions

The human erythroleukemia (K-562) cell line was obtained from the cell repository “Vertebrate cell culture collection” (supported by the Ministry of Science and Higher Education of the Russian Federation, agreement № 075-15-2021-683, Institute of Cytology, Russian Academy of Sciences, Saint Petersburg, Russia). Cells were grown on RPMI medium (Hyclone, GE Healthcare Life Sciences, Logan, UT, USA) supplemented with 10% (*v*/*v*) fetal bovine serum (Hyclone, GE Healthcare Life Sciences, Logan, UT, USA) and gentamicin (Sigma-Aldrich, St. Louis, MO, USA) at 37 °C in a humidified atmosphere with 5% CO_2_.

### 3.4. Cell Proliferation Assay

To evaluate the in vitro toxicity of the compounds synthesized, cells were seeded into 96-well plates at a density of 5 × 10^3^ cells per well. On the next day, the tested compounds were added to the wells at concentrations ranging from 1 to 30 mg/mL, followed by incubation for 1 and 3 days. Cell proliferation was determined by adding 20 μL of MTS reagent (BioVision, Milpitas, CA, USA) stock solution per well. The plate was incubated for 2 h at 37 °C in a humidified, 5% CO_2_ atmosphere. The plates were then read at 495 nm using a plate spectrophotometer (Multiskan GO, Thermo Fisher Scientific, Waltham, MA, USA). All samples were measured in triplicate. Data on K562 cells viability after the treatment for 24 h and 72h are given at Supporting Information, (Tables S3 and S4).

### 3.5. Statistical Analysis

Statistical processing of results was performed using Statistica 6.0. All data from the three independent experiments were used for measuring the means ± standard deviation (mean ± SD), which were compared using Student’s *t*-test or a nonparametric Wilcoxon Mann–Whitney U test.

## 4. Conclusions

In summary, we have shown for the first time the possibility of generating azomethine ylides from 11*H*-benzo[4,5]imidazo[1,2-*a*]indol-11-one and *α*−amino acids, and we also studied their 1,3-dipolar cycloaddition with cyclopropenes and maleimides. As a result of these reactions, novel spiro[benzo[4,5]imidazo[1,2-*a*]indole-11,2’-pyrrolidine] frameworks were obtained in moderate to good yields, albeit with low stereoselectivity. With the use of a DFT computational study, the observed poor diastereoselectivity was found to result from the formation of two isomeric azomethine ylides (S- and W-shaped dipoles), which subsequently react with cyclic dipolarophiles in a fully diastereoselective fashion. The results of antiproliferative activity study showed that the spiroadducts with a *N*-isopropylcarbamoyl group at the cyclopropane moiety were more active, while replacement of this group with a phenyl one led to significant decrease in the activity. It was found that, among the tested compounds, spiroadducts **4f** and **6i** demonstrated a significant activity, with IC_50_ 5 ± 1 and 12 ± 2 μg/mL, respectively. Despite its low stereoselectivity, the availability of numerous olefins and amino acids makes this method useful for accessing new hybrid spiro-heterocyclic systems containing simultaneously benzimidazole, indole, and pyrrolidine moieties. Current efforts are focused on increasing the stereoselectivity of cycloaddition reactions involving these new azomethine ylides and other dipolarophiles, as well as studying the biological properties of the products.

## Figures and Tables

**Table 1:** Optimization of the Reaction Conditions ^a^.

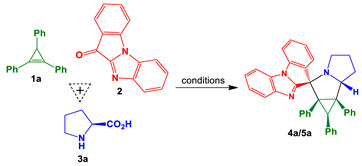				
Entry	Solvent	*T* (°C) ^c^	Time, h	Yield ^b^ of 4a/5a, % (dr)
1	EtOH	80	6	Trace
2	MeOH	60	6	Trace
3	THF	70	18	21 (2.6:1)
4	CH_3_CN	90	18	52 (1.8:1)
5	1,4-dioxane	100	18	64 (2:1)
6	benzene	80	18	27 (2.2:1)
7	toluene	110	18	39 (1.5:1)

^a^ Reaction conditions: 1a (1 equiv.), 2 (1 equiv.), 3a (2 equiv.), solvent. ^b^ Isolated yield. ^c^ The reaction was carried out in a sealed tube under a nitrogen atmosphere.

**Table 2:** Synthesis of Racemic Spiro-Adducts 4 via One-Pot Three-Component Reactions of Cyclopropenes 1a-h, Ketone 2 and *L*-Proline (3a) ^a,b,e.^

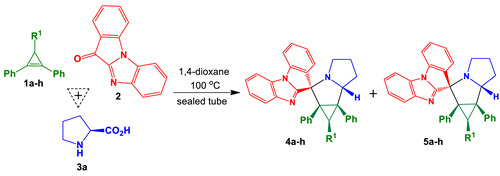
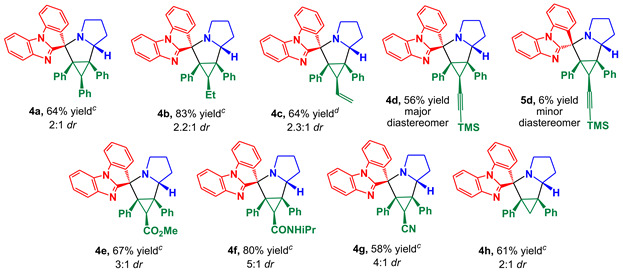

^a^ Reactions of 1 (0.3 mmol), 2 (0.3 mmol), and 3 (0.6 mmol) were carried out in 1,4-dioxane (4 mL) in a sealed tube at 100 °C for 18 h under a nitrogen atmosphere. ^b^ Isolated yield. ^c^ Crystallization of the mixture from methanol gave the major diastereomer 4 as a pure compound. ^d^ Inseparable mixture of diastereomers. ^e^ The *dr* values were determined by ^1^H NMR of the crude mixture.

**Table 3:** Synthesis of Racemic Spiro-Adducts 6 via One-Pot Three-Component Reactions of Cyclopropenes 1a-d,f,h, Ketone 2 and Primary *α*-Amino Acids 3b–f ^a,b,c,d.^

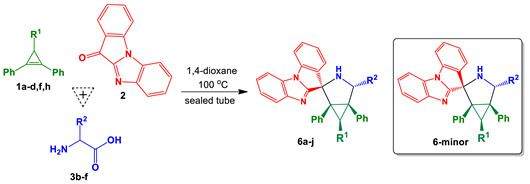
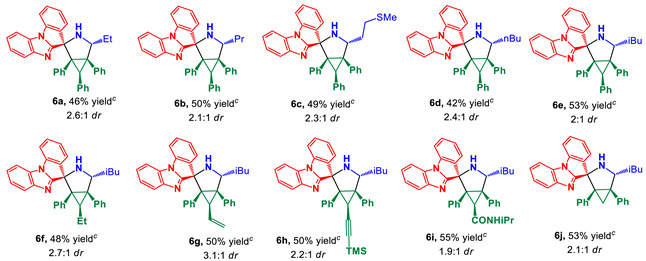

^a^ Reactions of 1 (0.3 mmol), 2 (0.3 mmol), and 3 (0.6 mmol) were carried out in 1,4-dioxane (4 mL) in a sealed tube at 100 °C for 18 h under a nitrogen atmosphere. ^b^ Isolated yield. ^c^ Crystallization of the diastereomeric mixture from methanol gave the major diastereomer 6 as a pure compound. ^d^ The *dr* values were determined by ^1^H NMR of the crude mixture.

**Table 4:** Synthesis of Racemic Spiro-Adducts 8 via One-Pot Three-Component Reactions of Maleimides 7a–f, Ketone 2 and *L*-Proline 3a ^a,b,c.^

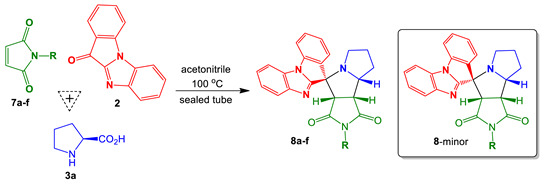
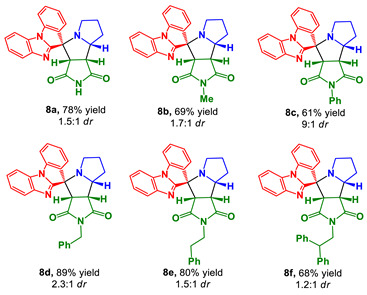

^a^ Reactions of 7 (0.25 mmol), 2 (0.25 mmol), and 3a (0.5 mmol) were carried out in acetonitrile (4 mL) in a sealed tube at 100 °C for 18 h under a nitrogen atmosphere. ^b^ Isolated yield. ^c^ The *dr* values were determined by ^1^H NMR of the crude mixture.

## Data Availability

The data are available in the Supplementary Section.

## Supplementary Materials

The supporting information can be downloaded at: https://www.mdpi.com/article/10.3390/ijms232113202/s1. References [55,56,57,58,59,60,61,62,63,64] are cited in the supplementary materials.
