# Supplementary material for: 11H-Benzo[4,5]imidazo[1,2-a]indol-11-one as a New Precursor of Azomethine Ylides: 1,3-Dipolar Cycloaddition Reactions with Cyclopropenes and Maleimides
Source: Int J Mol Sci. 2022 Oct 30;23(21):13202. doi: 10.3390/ijms232113202 (PMC9657675; doi:10.3390/ijms232113202)
Supplement: Supplementary file 1 [file ijms-23-13202-s001.zip › ijms-1943782-supplementary.pdf]

Supporting information

11*H*-Benzo[4,5]imidazo[1,2-*a*]indol-11-one as a New Precursor of Azomethine Ylides: 1,3-Dipolar Cycloaddition reactions with Cyclopropenes and Maleimides

Alexander S. Filatov[a], Yulia A. Pronina[b], Stanislav I. Selivanov[a], Stanislav V. Shmakov[c], Anton A. Uspenski[d], Vitali M. Boitsov*[c], Alexander V. Stepakov*[a,b]

[a] *Saint-Petersburg State University, Universitetskaya nab. 7/9, 199034, St. Petersburg, Russian Federation*

[b] *Saint-Petersburg State Institute of Technology, Moskovskii pr. 26, 190013, St. Petersburg, Russian Federation*

[c] *Saint-Petersburg Academic University – Nanotechnology Research and Education Centre RAS, ul. Khlopina 8/3, 194021, St. Petersburg, Russian Federation*

[d] *Voeikov Main Geophysical Observatory, ul. Karbysheva 7, 194021, St. Petersburg, Russian Federation.*

Table of contents

**1.**	Copies of ^{1}H and ^{13}C NMR spectra of compounds **4-6** and **8**	S2
**2.**	The 2D NMR analysis of the compounds **4d** and **5d**	S28
**3.**	X-ray crystallographic data for compounds **4f** and **8f**	S34
**4.**	Bioassay details	S36
**5**	Computational data	S38
**6.**	References	S54

1. Copies of ¹H and ¹³C NMR spectra of compounds 4 and 6

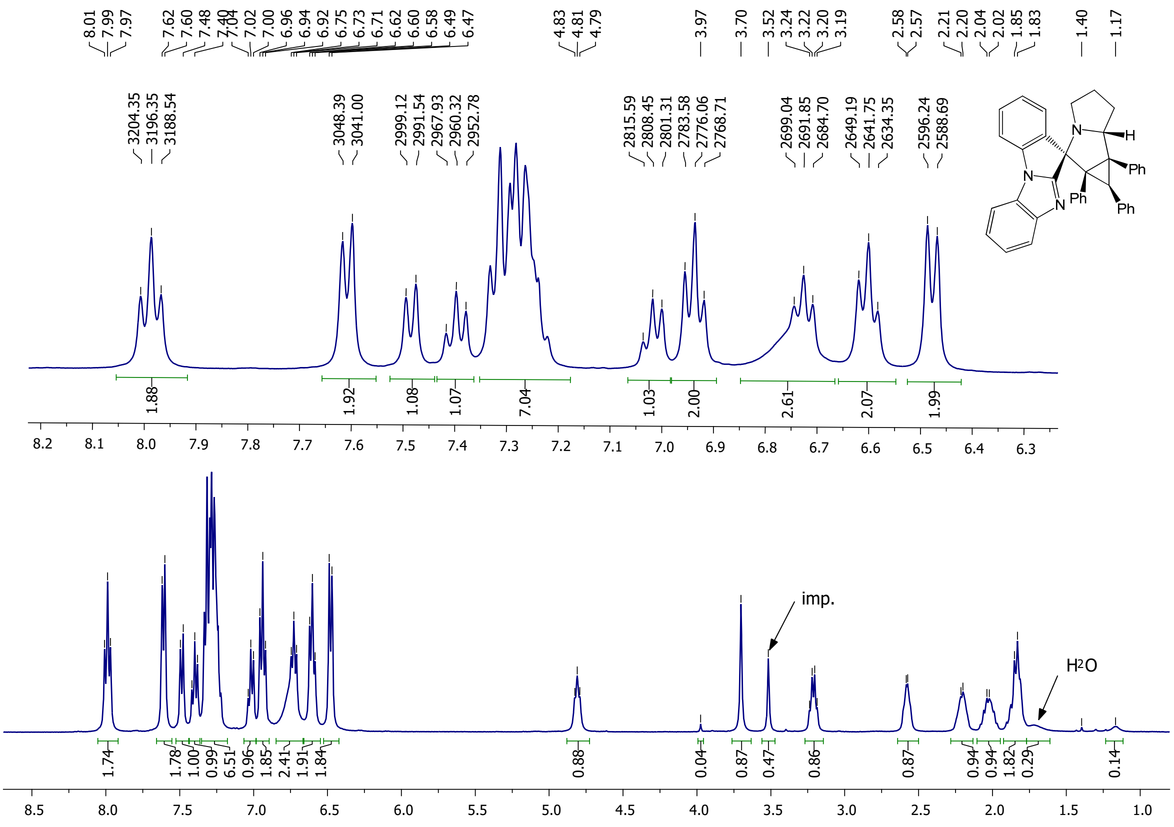

Figure S1. ¹H NMR spectrum of compound **4a** (CDCl₃, 400 MHz)

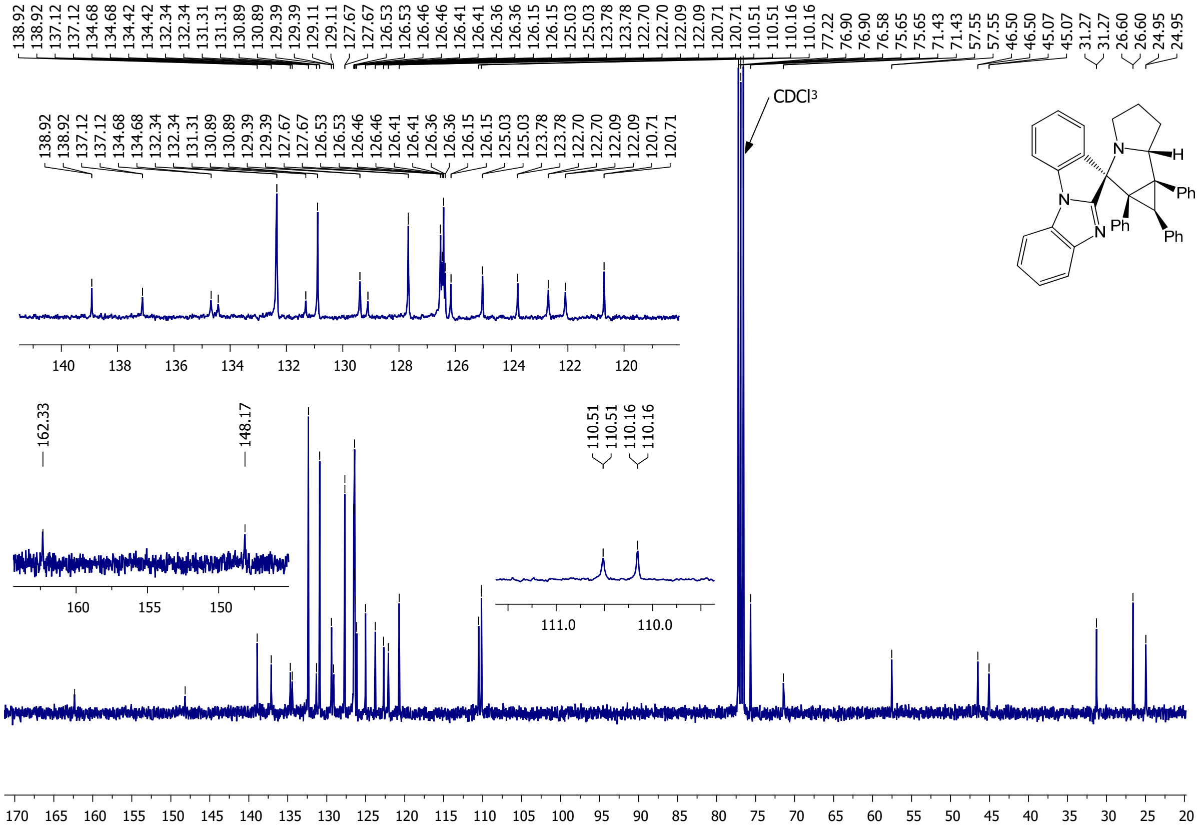

Figure S2. ¹³C NMR spectrum of compound **4a** (CDCl₃, 101 MHz)

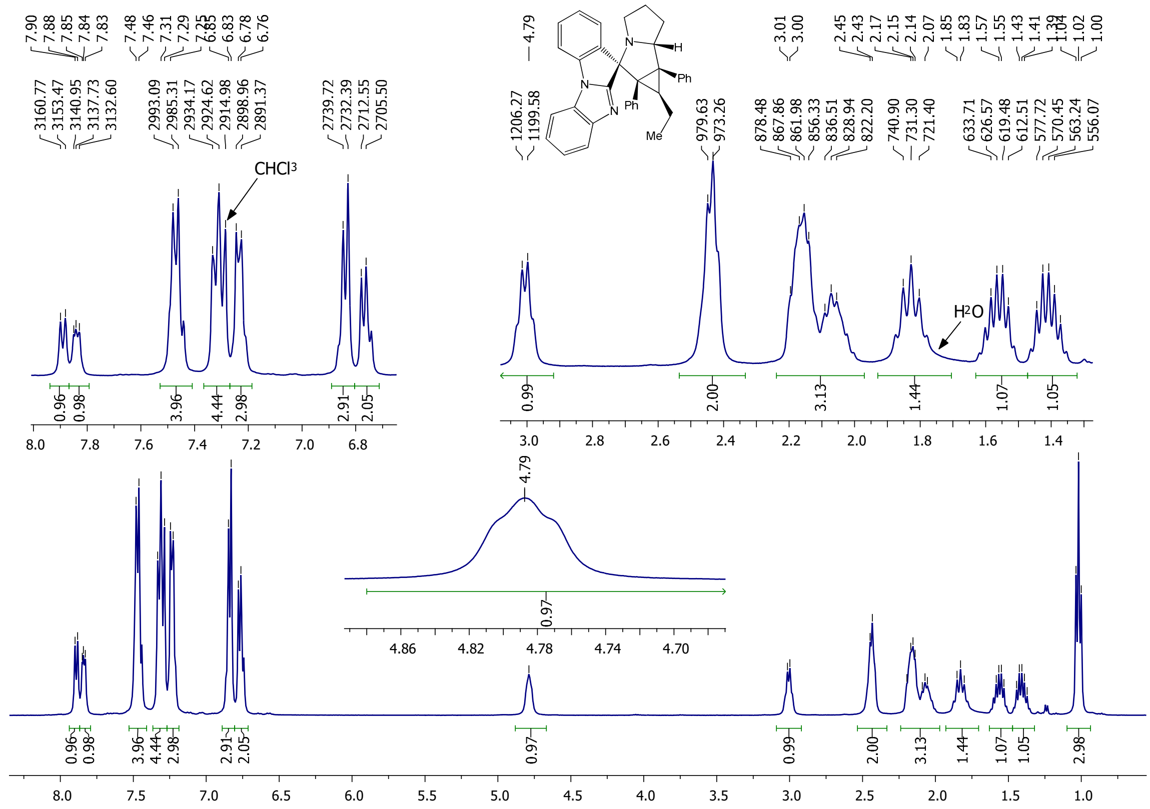

Figure S3. ^{1}H NMR spectrum of compound **4b** ($CDCl_3$, 400 MHz)

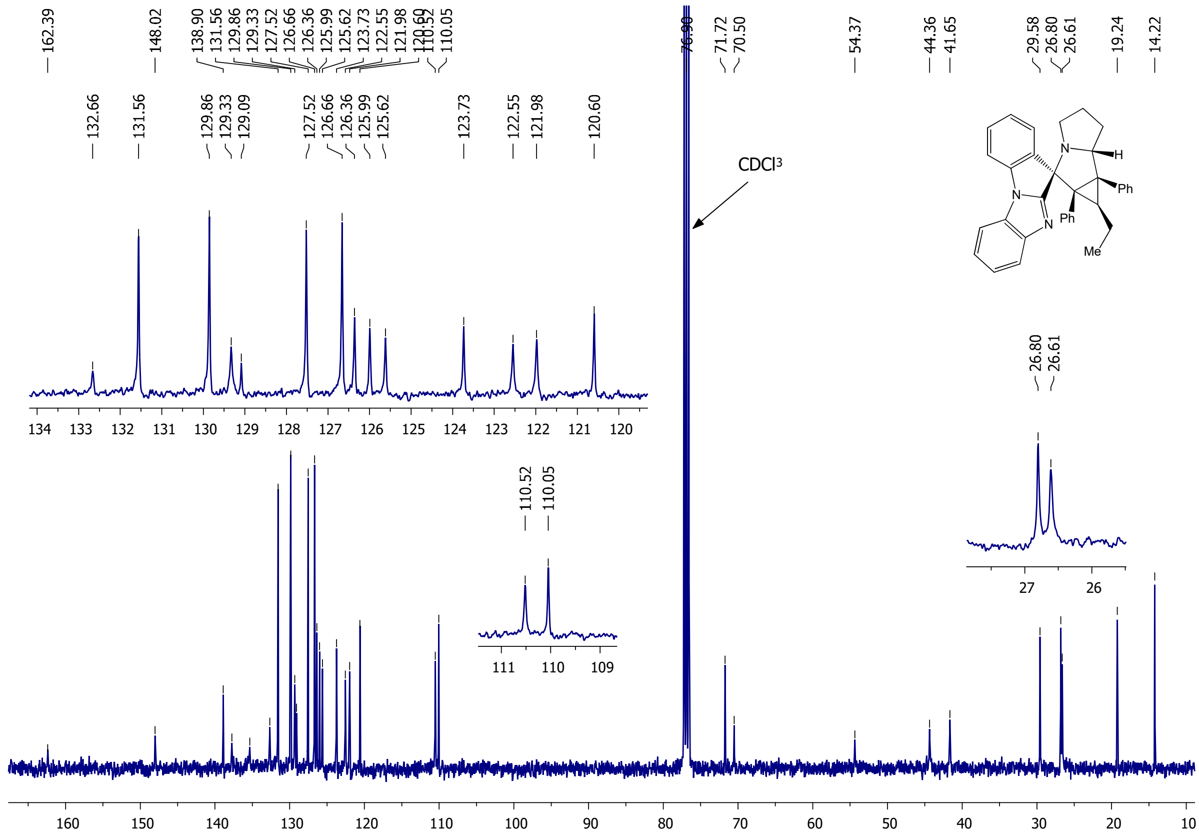

Figure S4. ^{13}C NMR spectrum of compound **4b** ($CDCl_3$, 400 MHz)

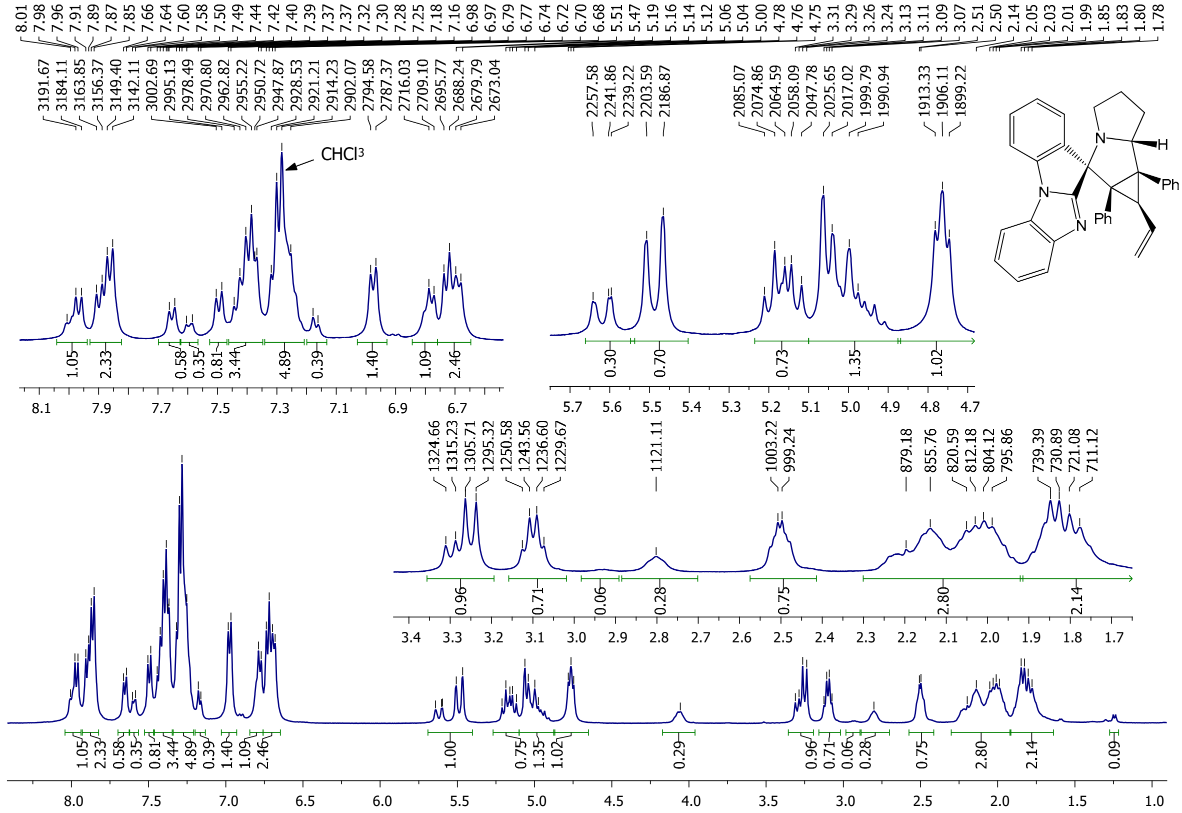

Figure S5. ^{1}H NMR spectrum of compound **4c** ($CDCl_3$, 400 MHz)

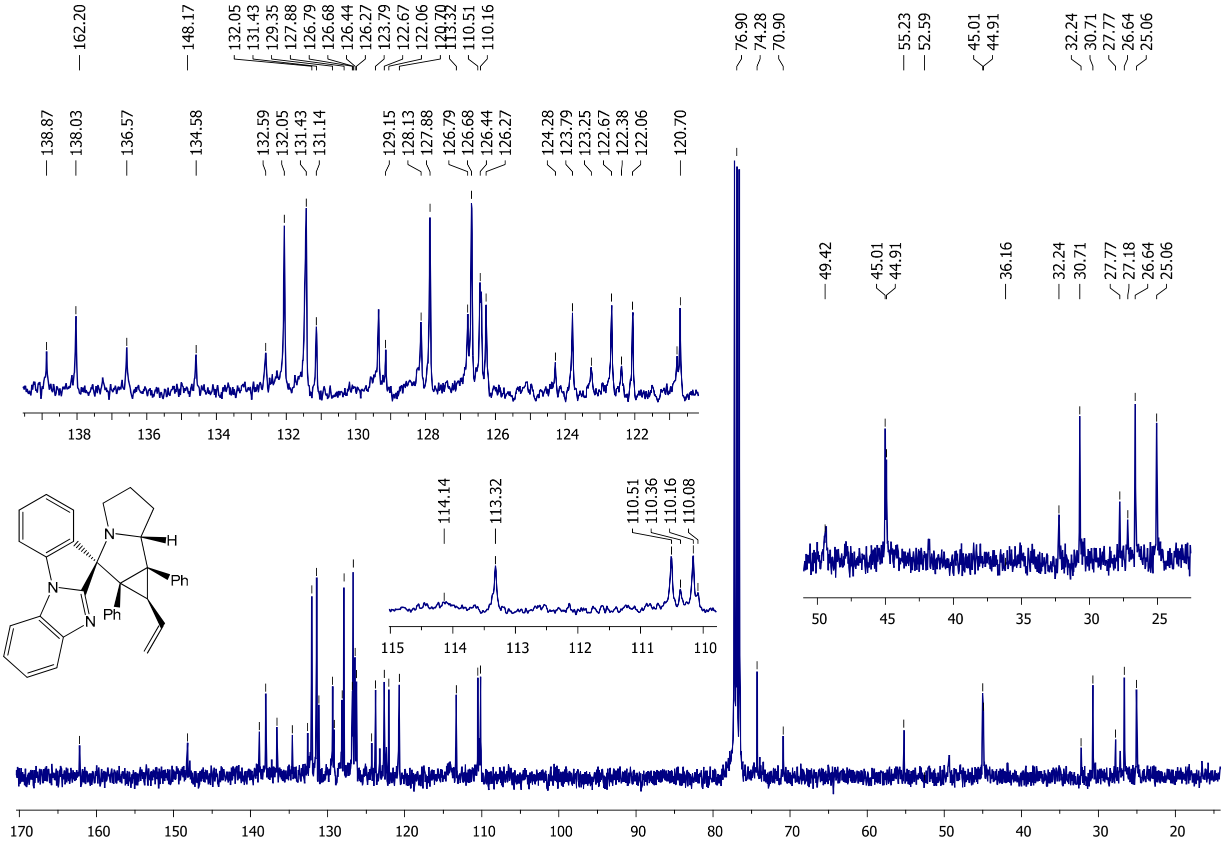

Figure S6. ^{13}C NMR spectrum of compound **4c** ($CDCl_3$, 400 MHz)

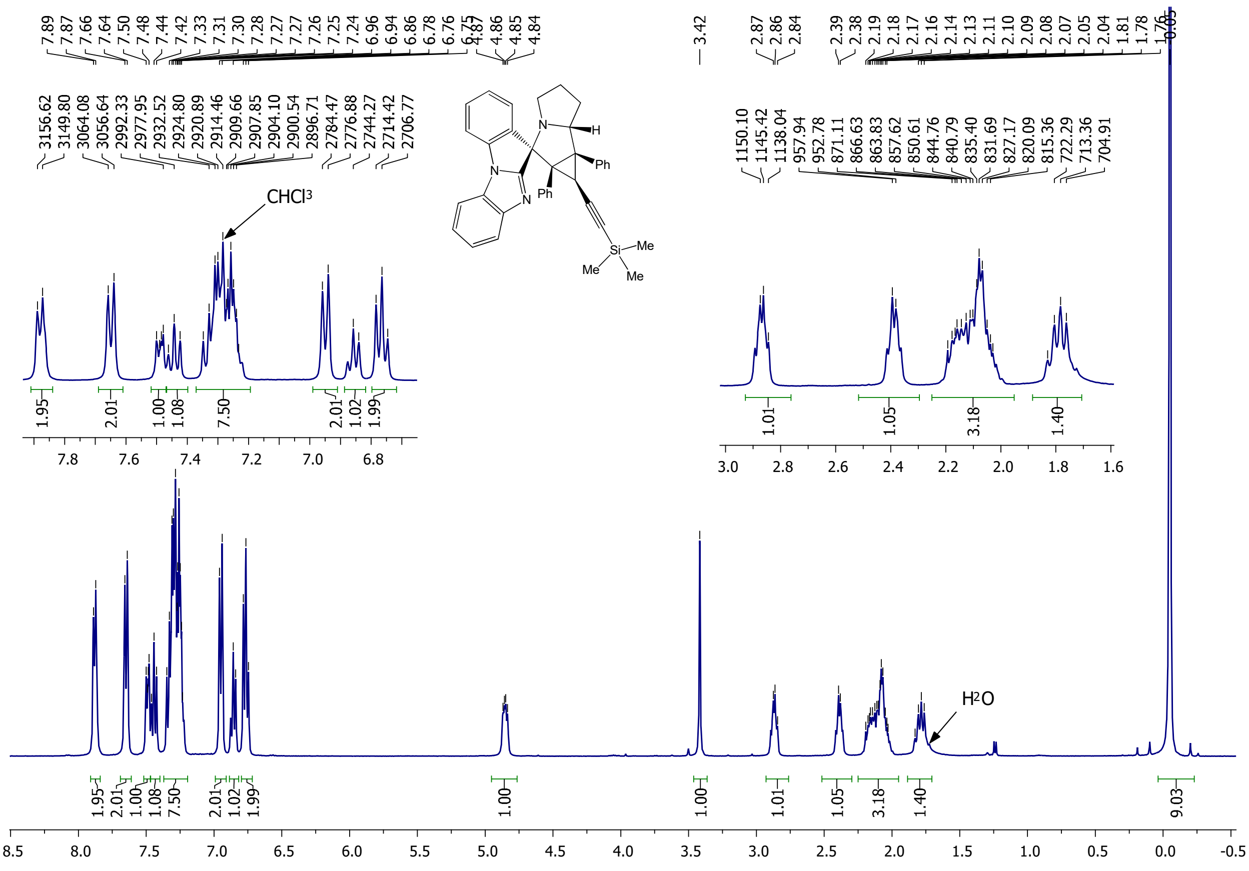

Figure S7. ^{1}H NMR spectrum of compound **4d** ($CDCl_3$, 400 MHz)

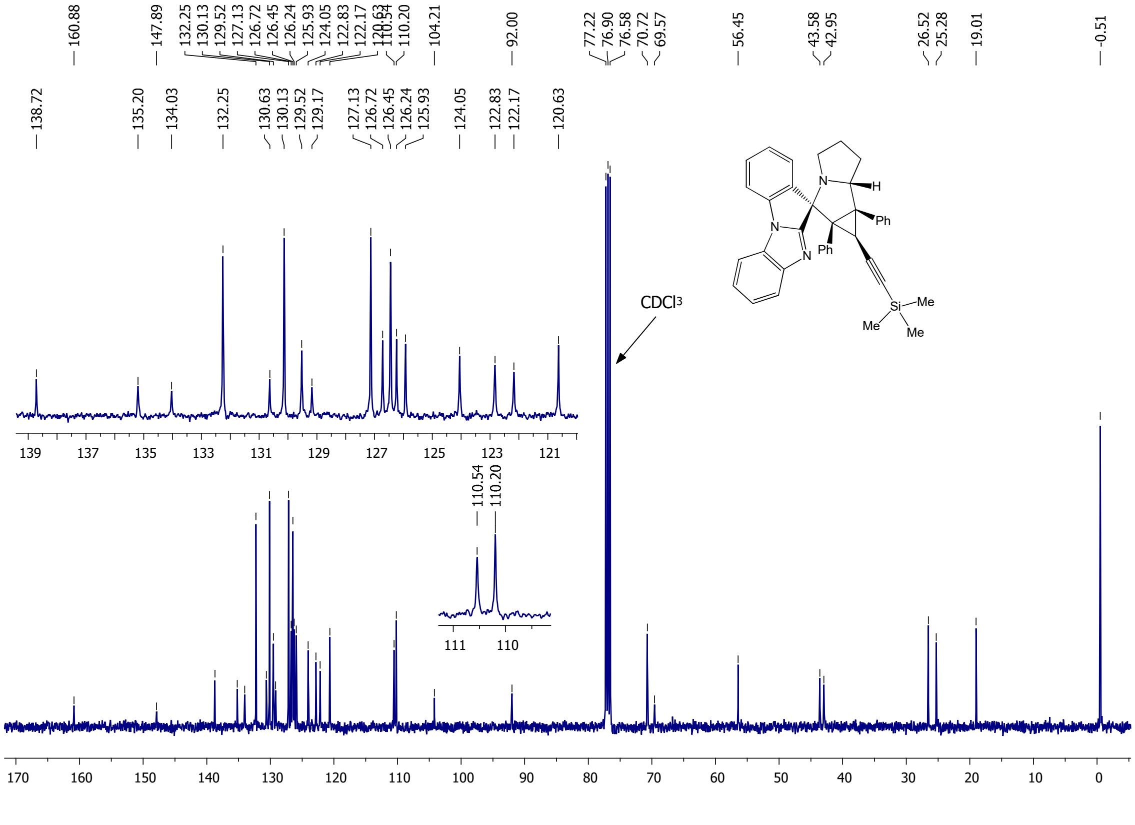

Figure S8. ^{13}C NMR spectrum of compound **4d** ($CDCl_3$, 400 MHz)

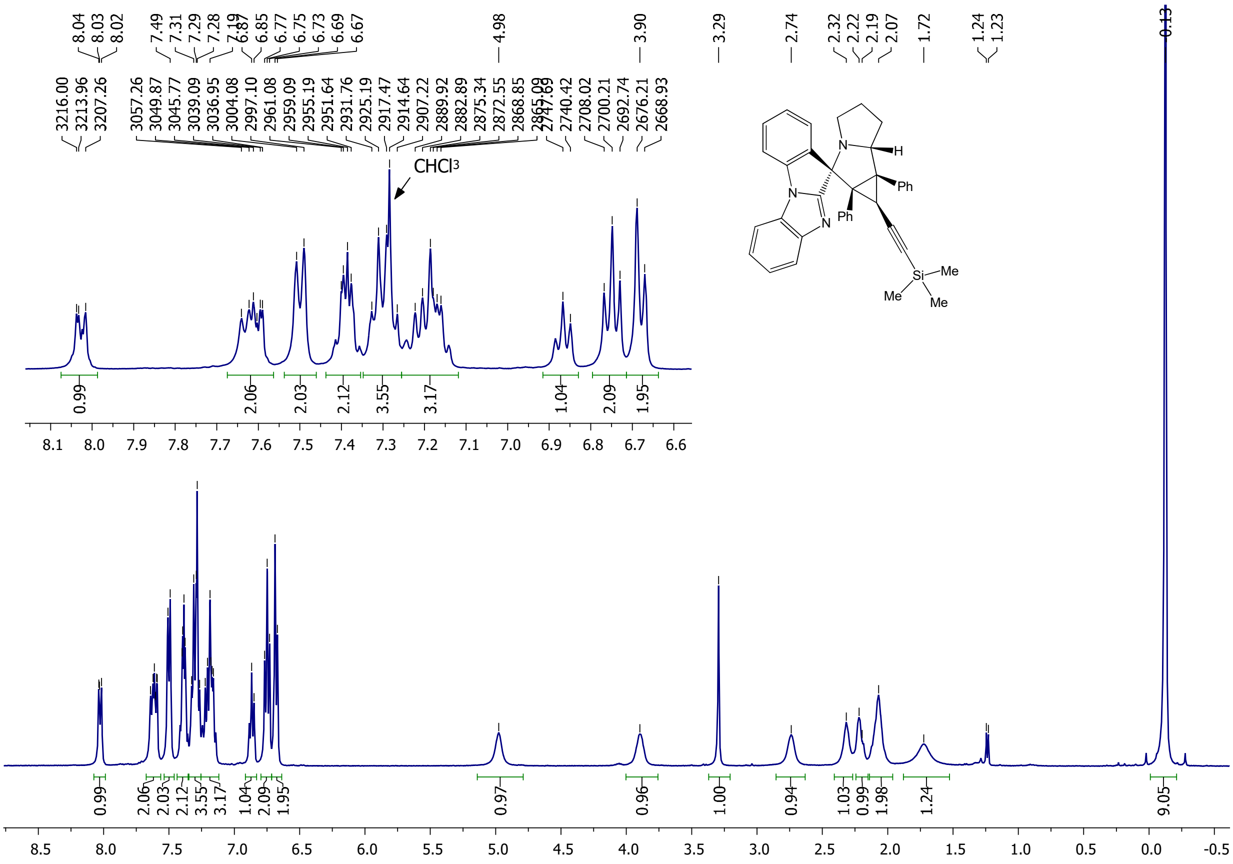

Figure S9. ^{1}H NMR spectrum of compound **5d** ($CDCl_3$, 400 MHz)

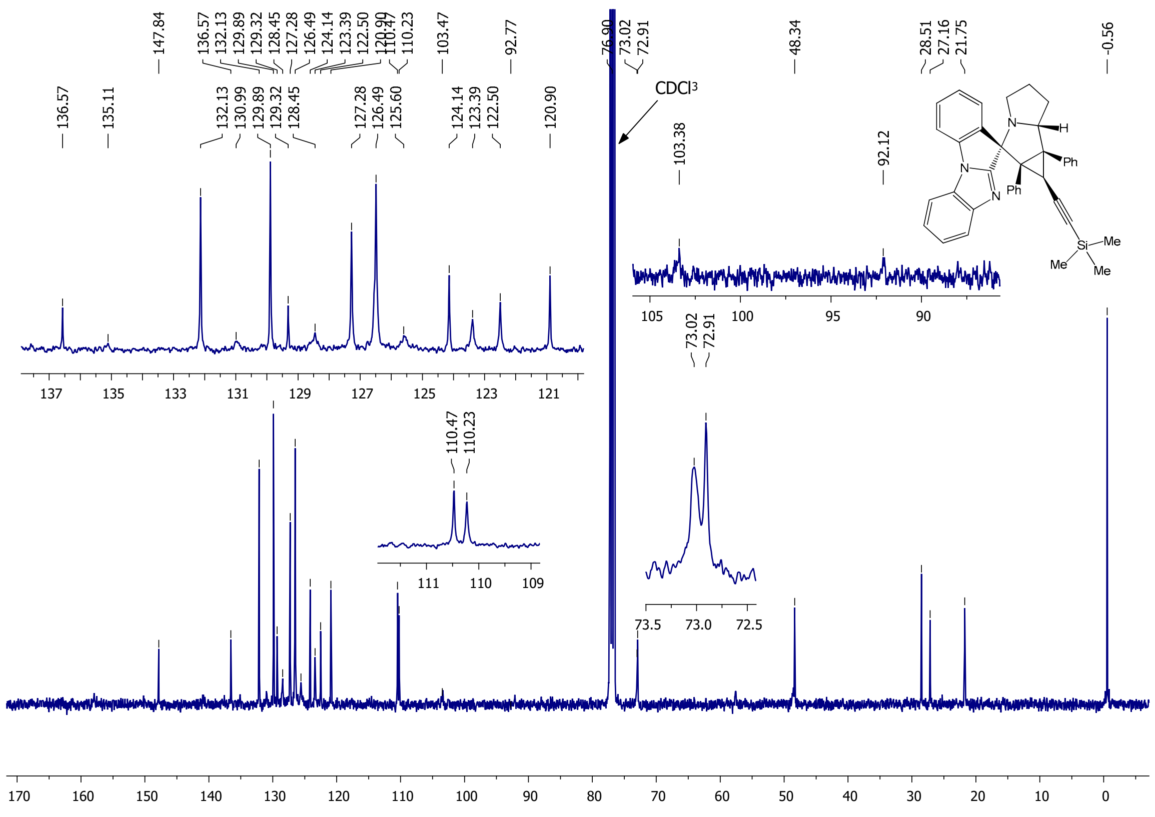

Figure S10. ^{13}C NMR spectrum of compound **5d** ($CDCl_3$, 400 MHz)

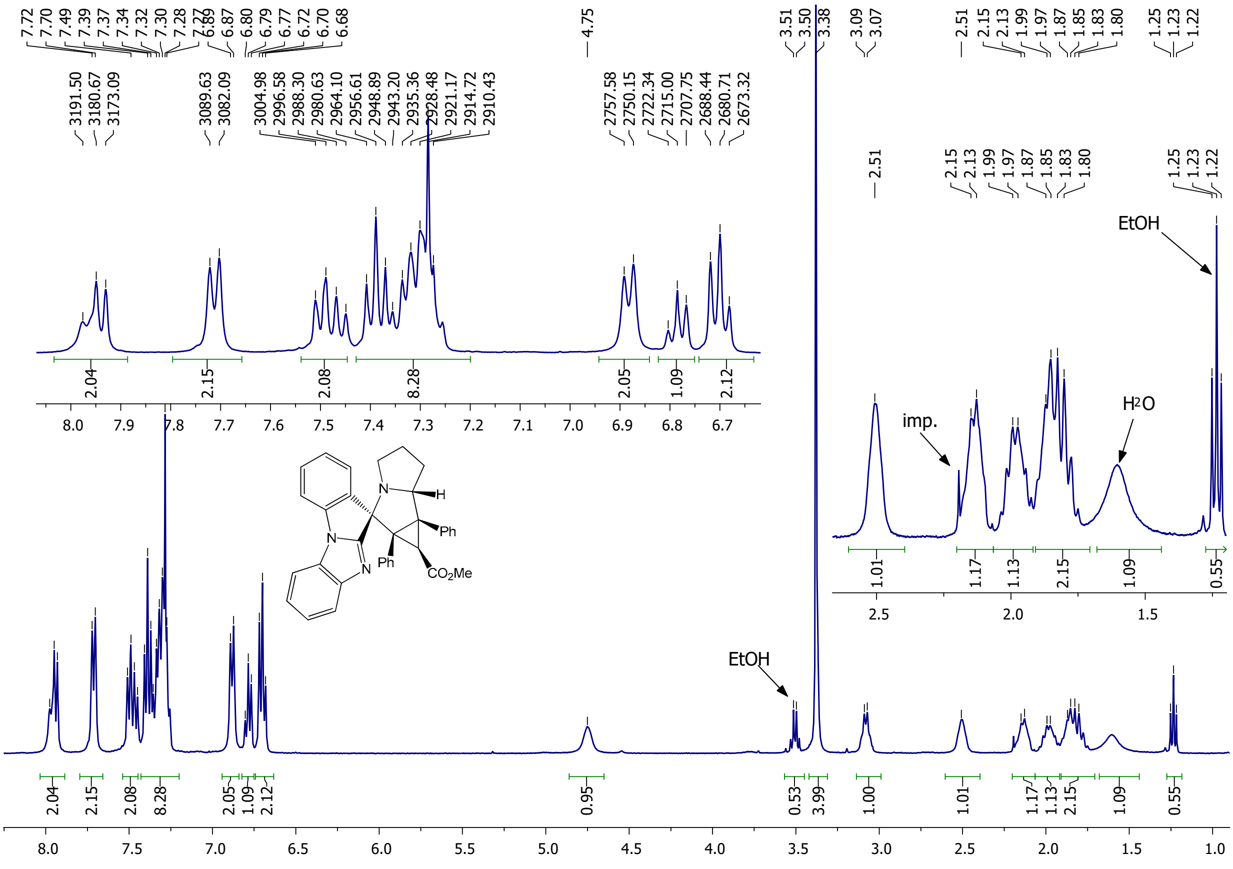

Figure S11. ^{1}H NMR spectrum of compound **4e**($CDCl_3$, 400 MHz)

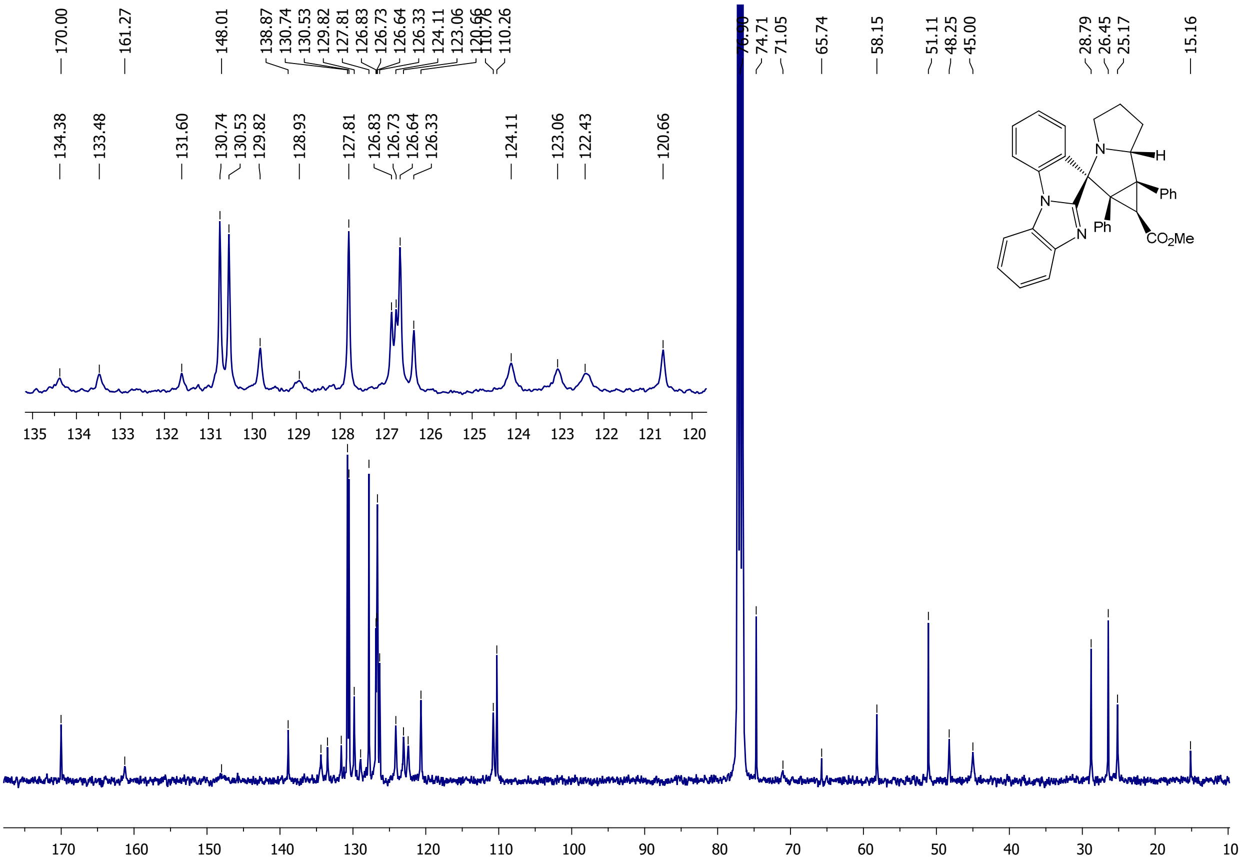

Figure S12. ^{13}C NMR spectrum of compound **4e** ($CDCl_3$, 101 MHz)

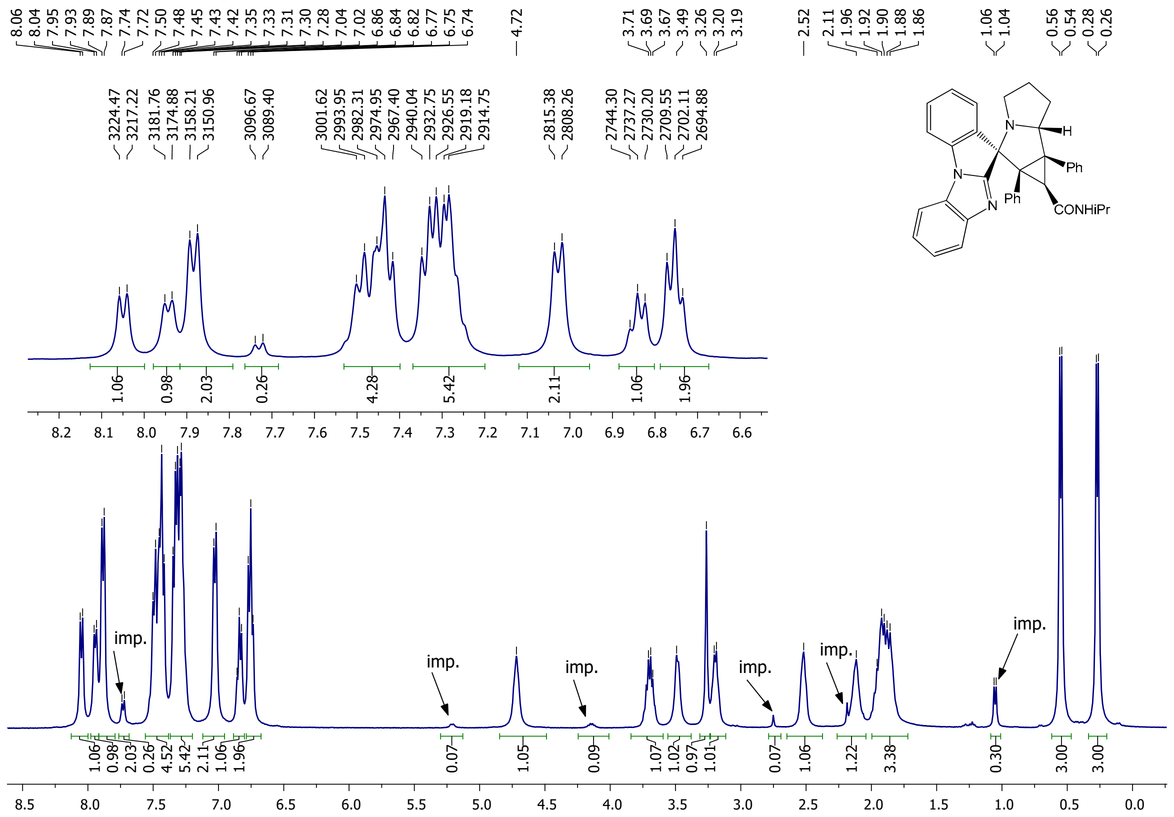

Figure S13. ^{1}H NMR spectrum of compound **4f** ($CDCl_3$, 400 MHz)

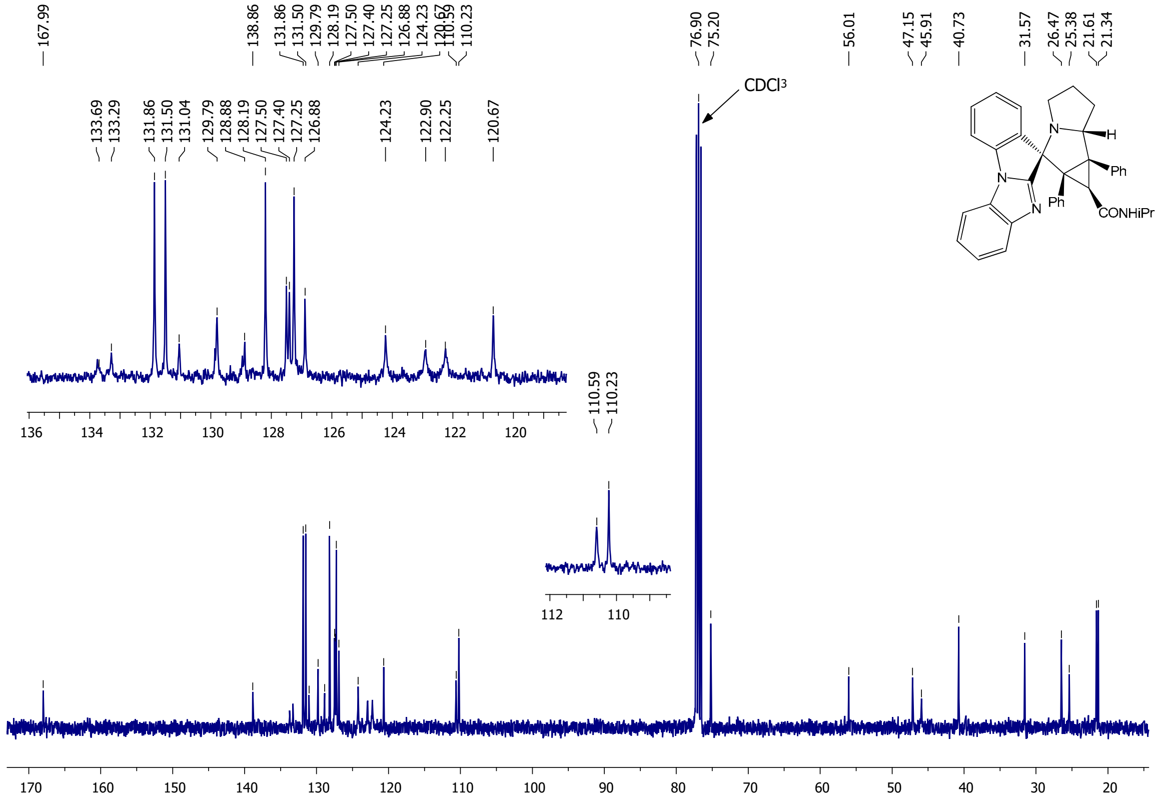

Figure S14. ^{13}C NMR spectrum of compound **4f** ($CDCl_3$, 101 MHz)

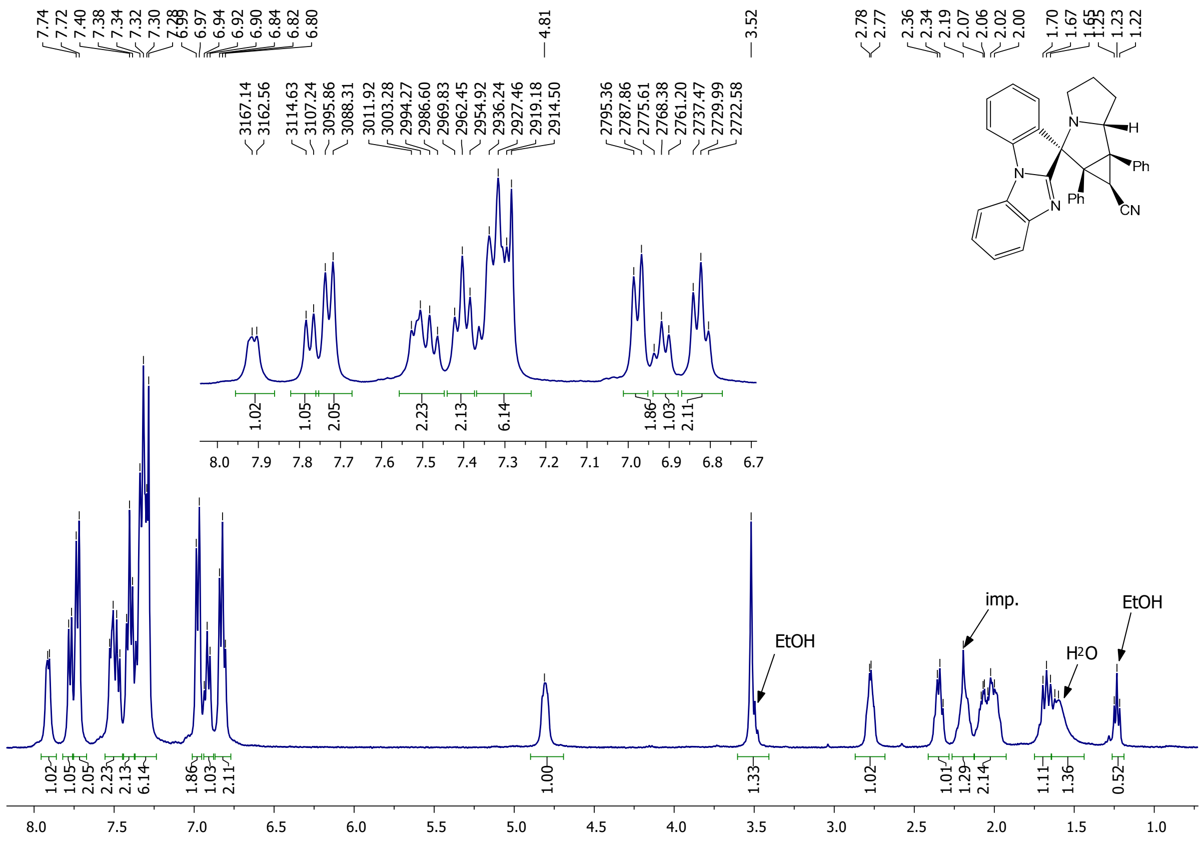

Figure S15. ^{1}H NMR spectrum of compound **4g** ($CDCl_3$, 400 MHz)

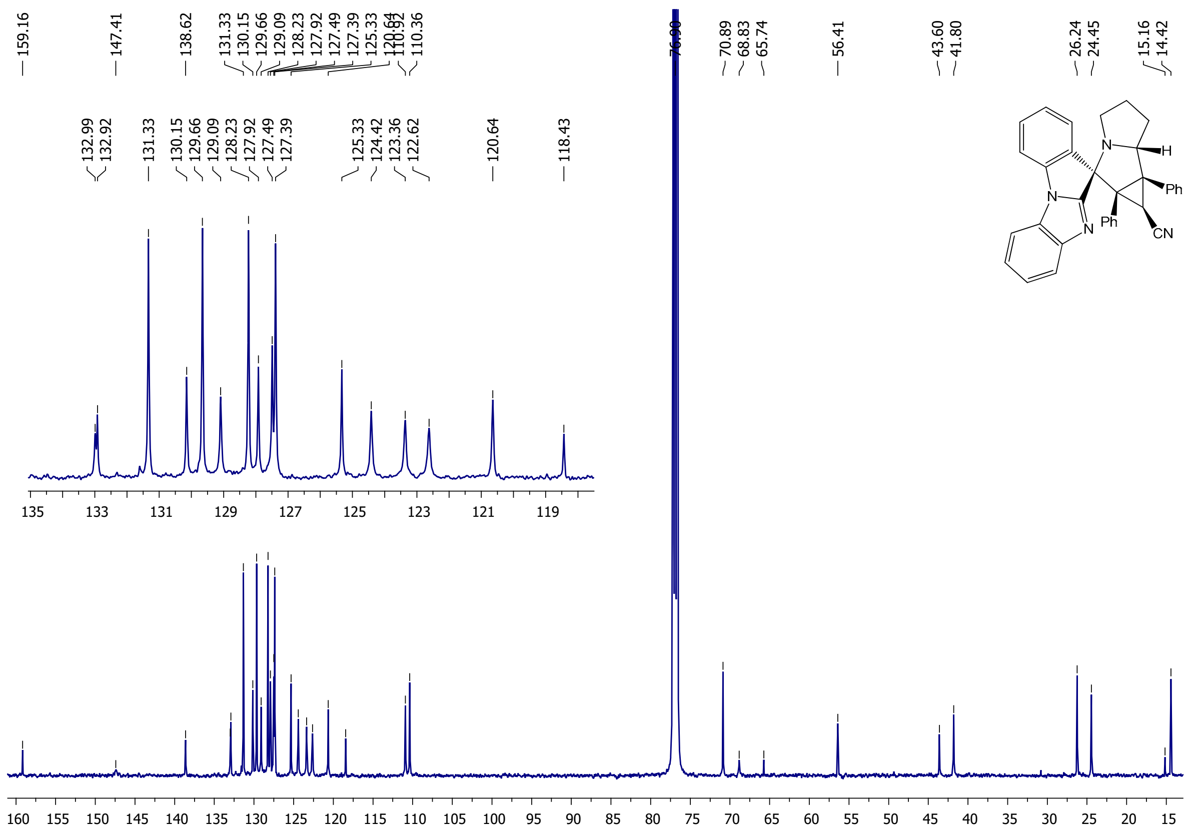

Figure 16. ^{13}C NMR spectrum of compound **4g** ($CDCl_3$, 101 MHz)

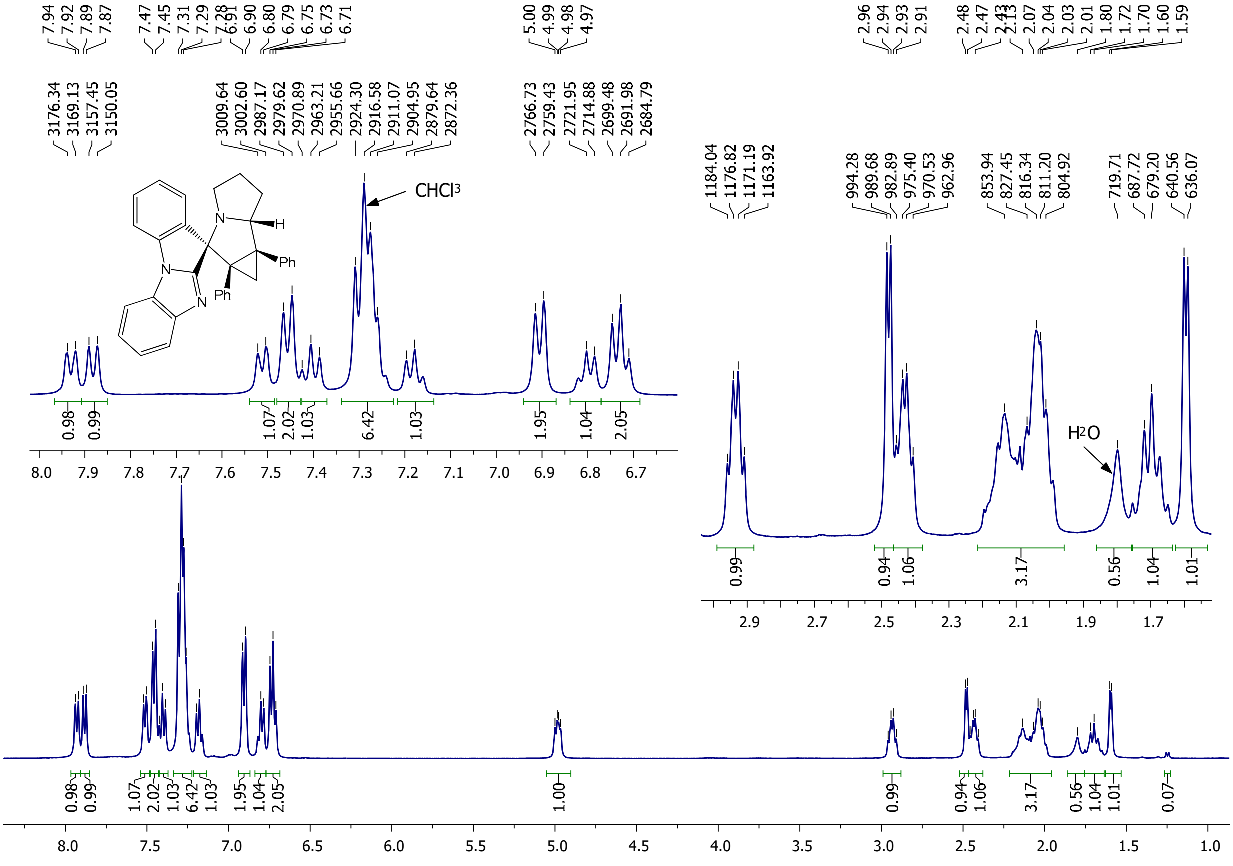

Figure S17. ^{1}H NMR spectrum of compound **4h** ($CDCl_3$, 400 MHz)

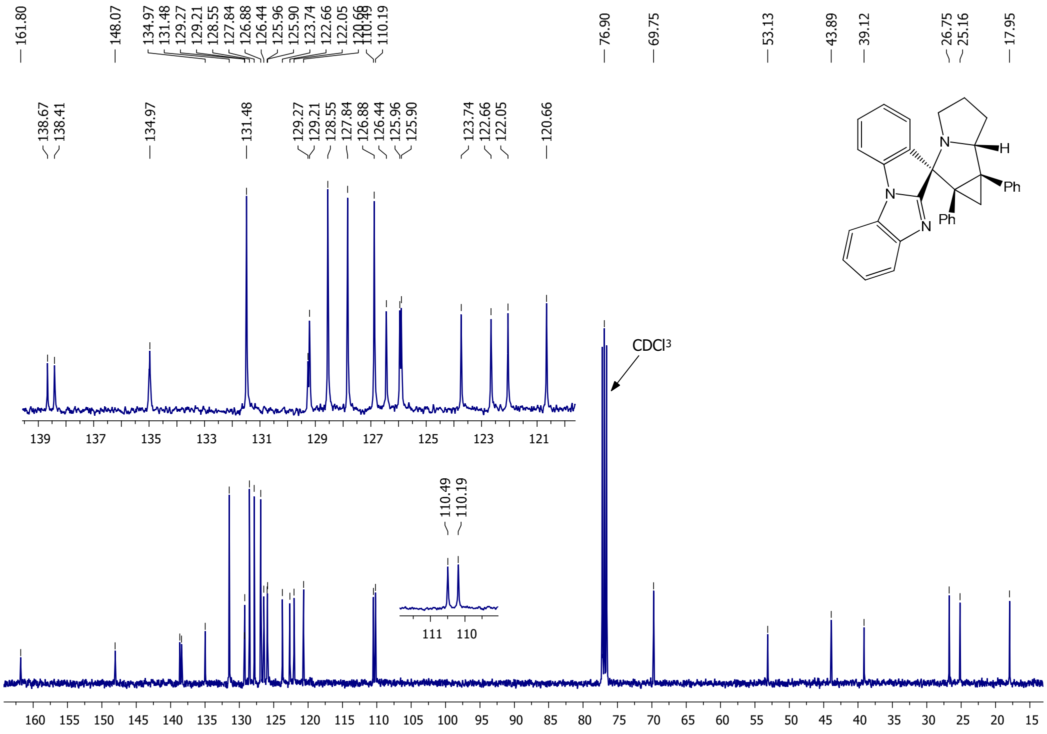

Figure S18. ^{13}C NMR spectrum of compound **4h** ($CDCl_3$, 400 MHz)

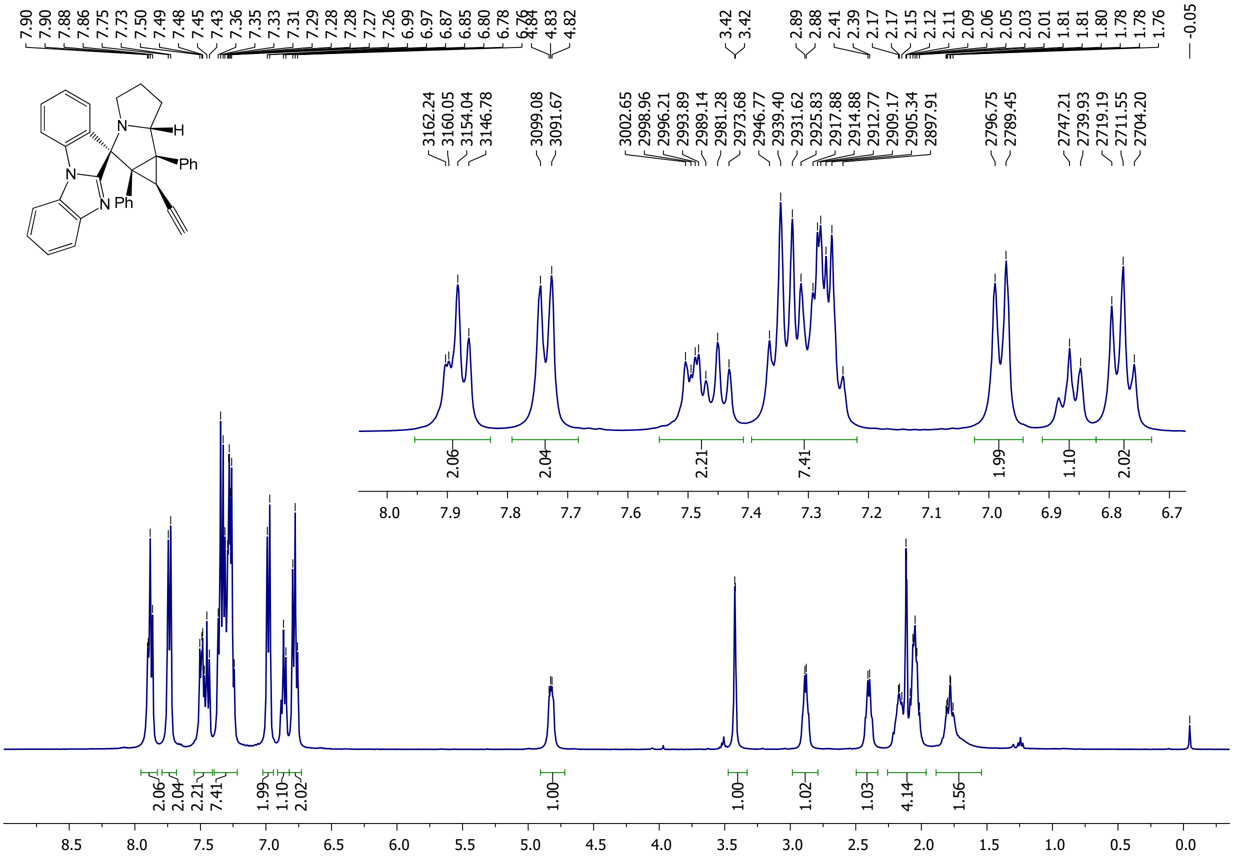

Figure S19. ^{1}H NMR spectrum of compound **4i** ($CDCl_3$, 400 MHz)

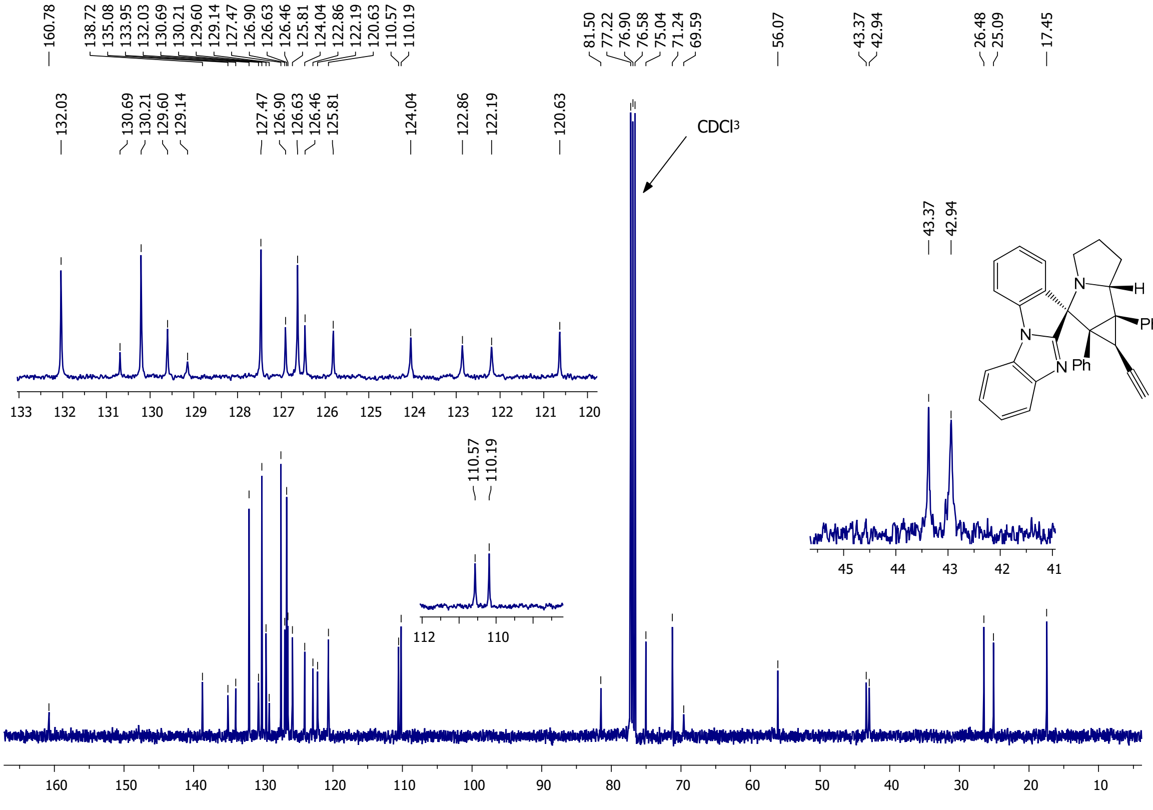

Figure S20. ^{13}C NMR spectrum of compound **4i** ($CDCl_3$, 101 MHz)

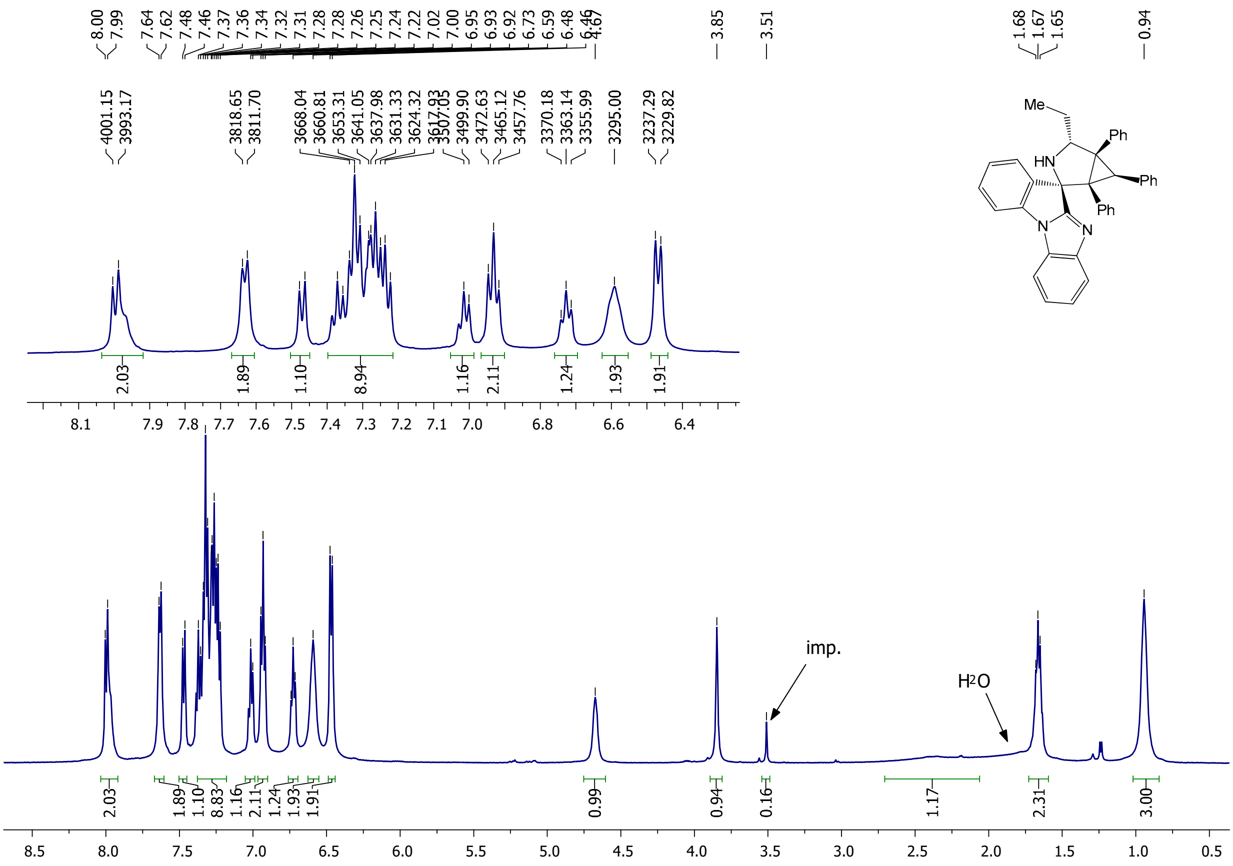

Figure S21. ^{1}H NMR spectrum of compound **6a** ($CDCl_3$, 400 MHz)

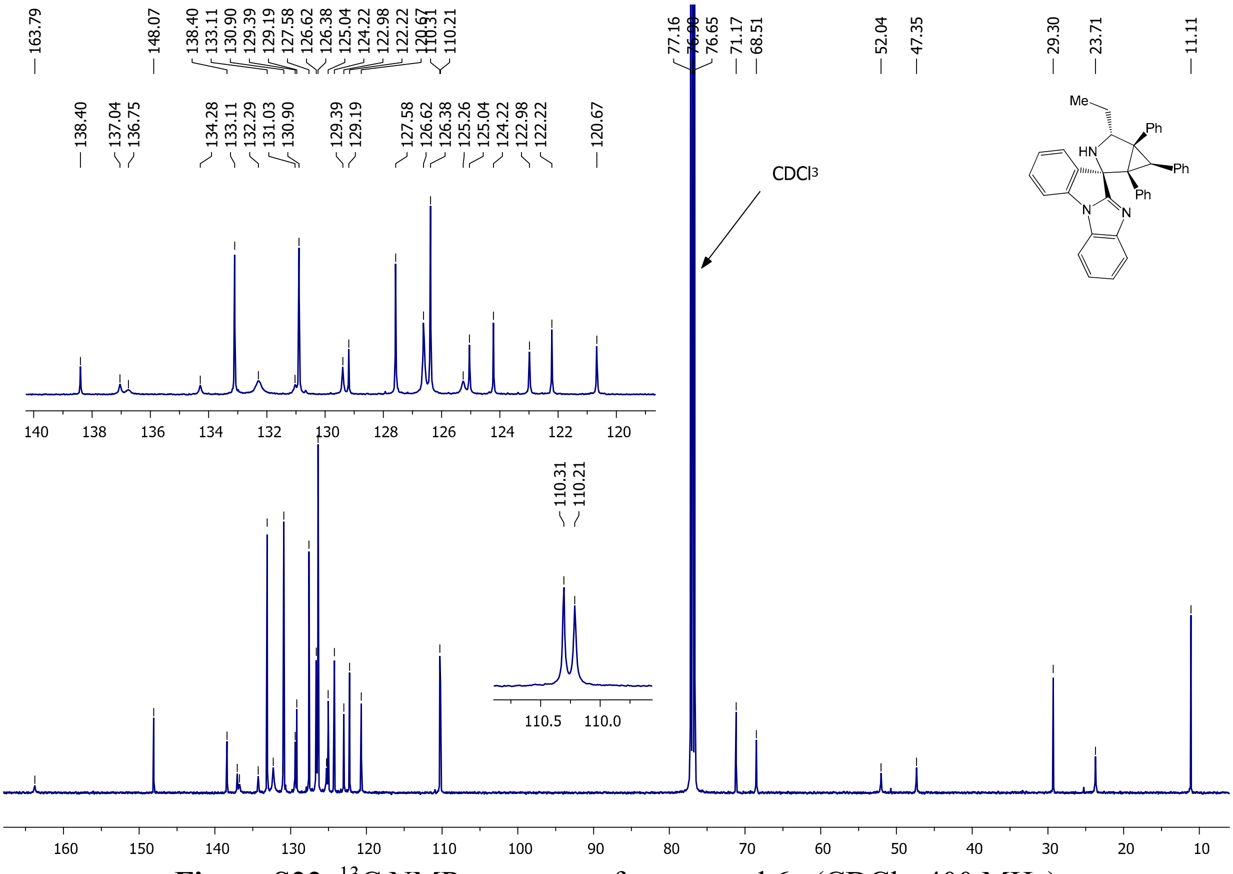

Figure S22. ^{13}C NMR spectrum of compound **6a** ($CDCl_3$, 400 MHz)

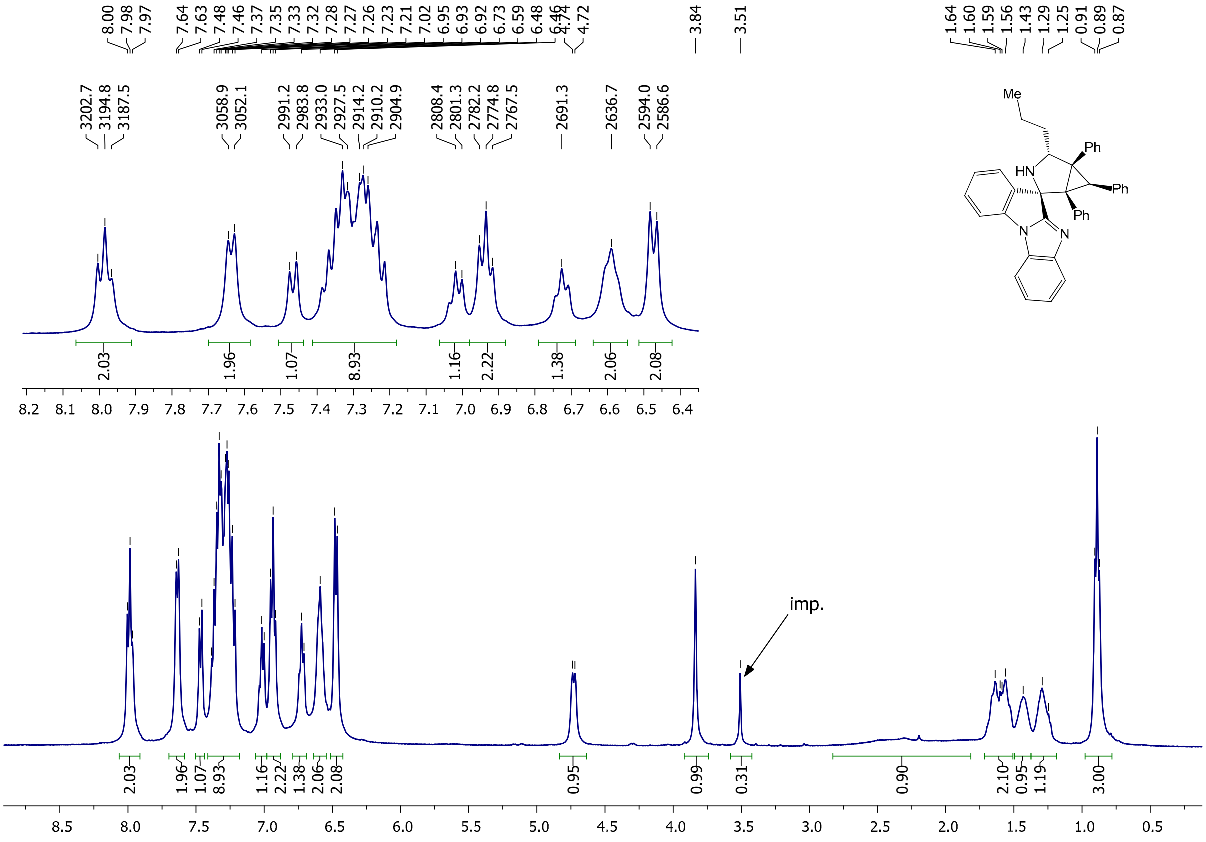

Figure S23. ^{1}H NMR spectrum of compound **6b** ($CDCl_3$, 400 MHz)

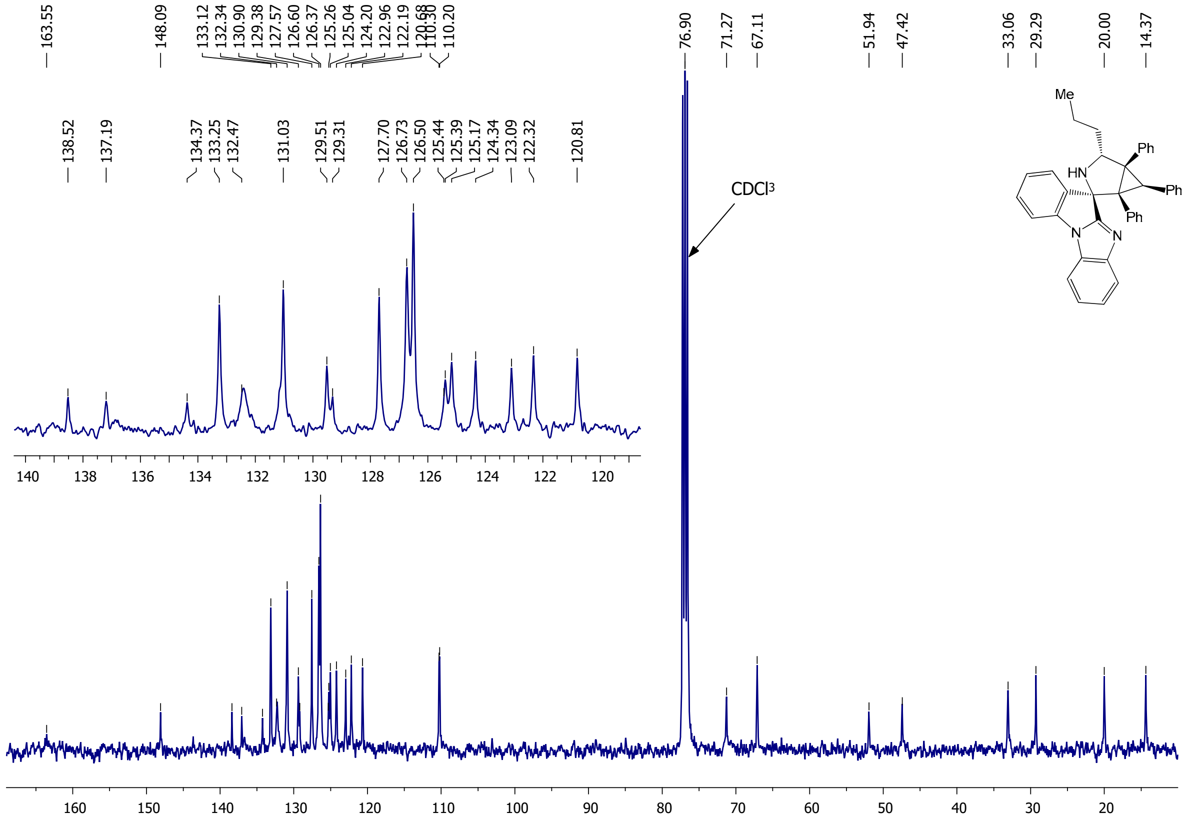

Figure S24. ^{13}C NMR spectrum of compound **6b** ($CDCl_3$, 400 MHz)

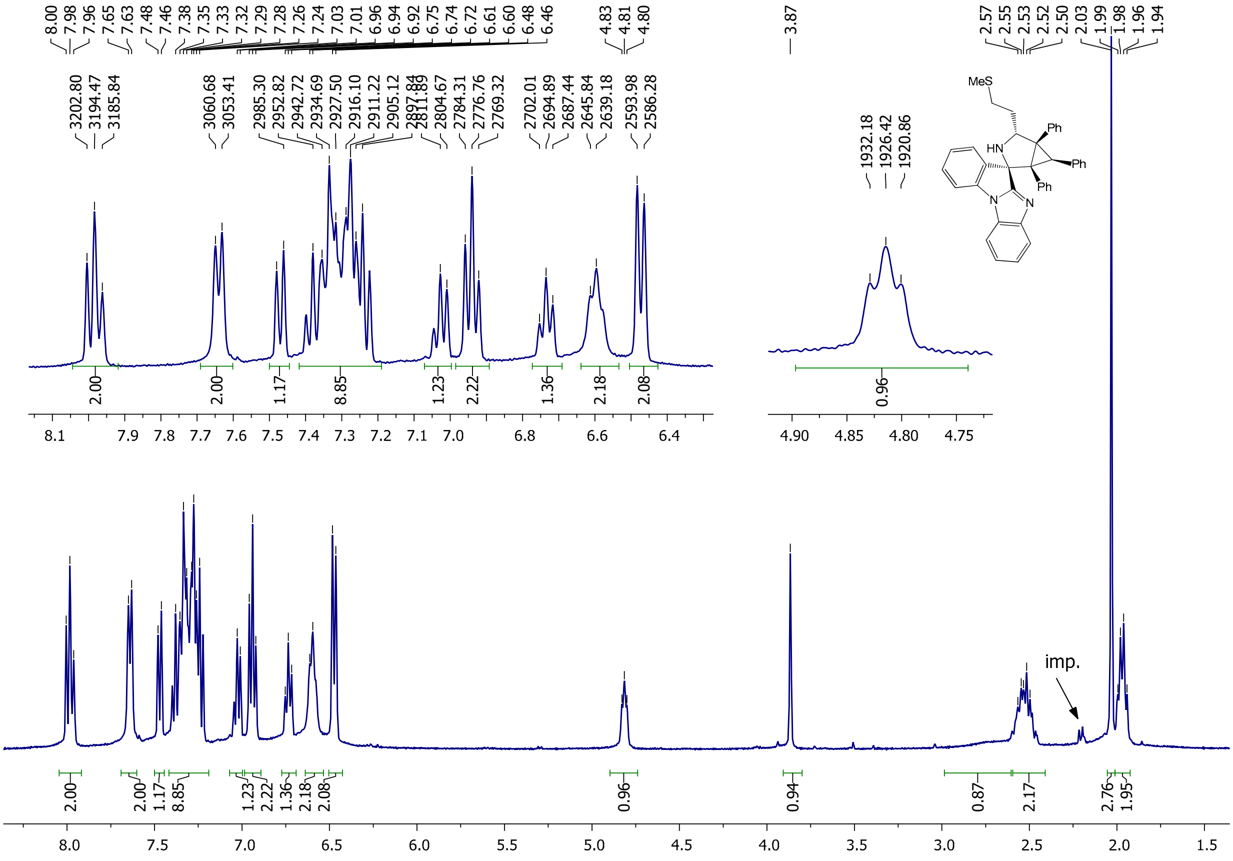

Figure S25. ^{1}H NMR spectrum of compound **6c** ($CDCl_3$, 400 MHz)

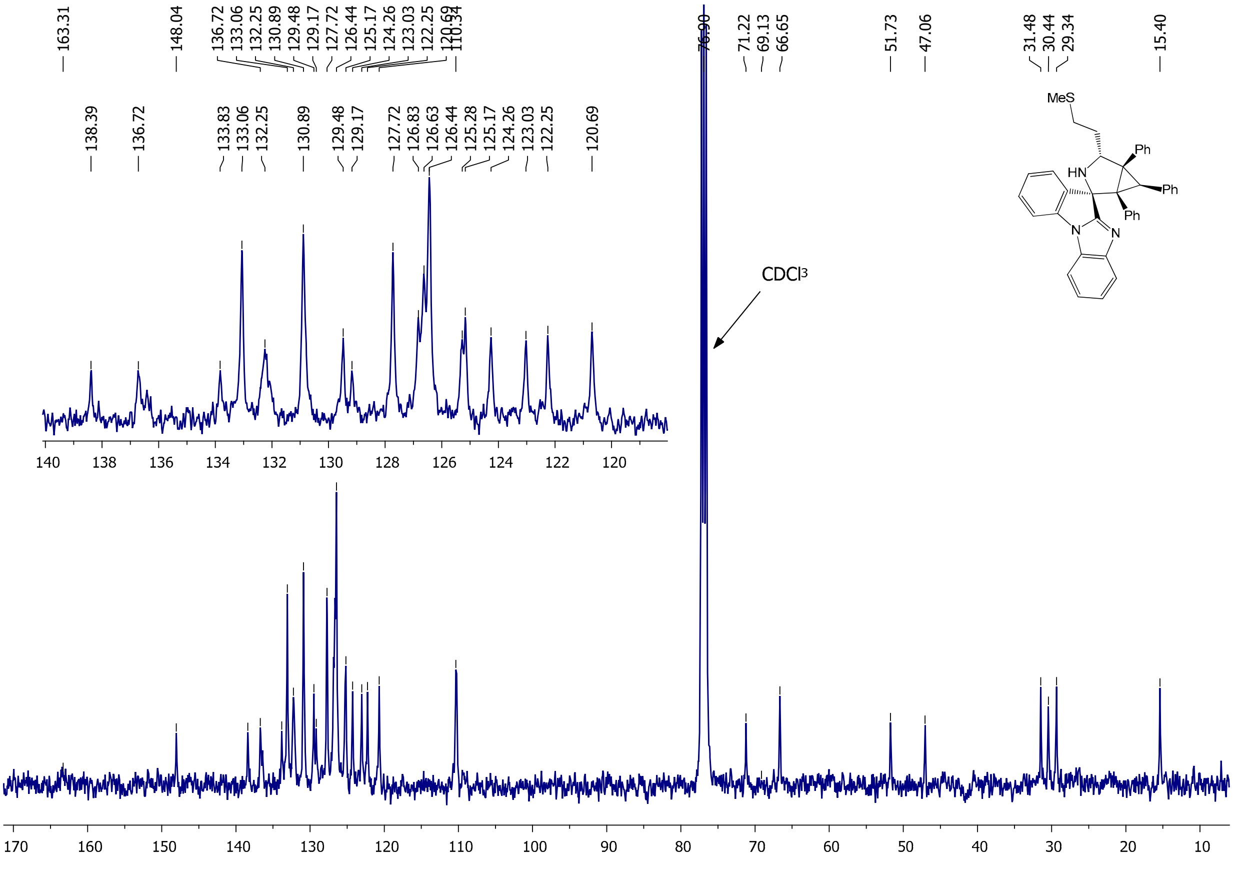

Figure S26. ^{13}C NMR spectrum of compound **6c** ($CDCl_3$, 400 MHz)

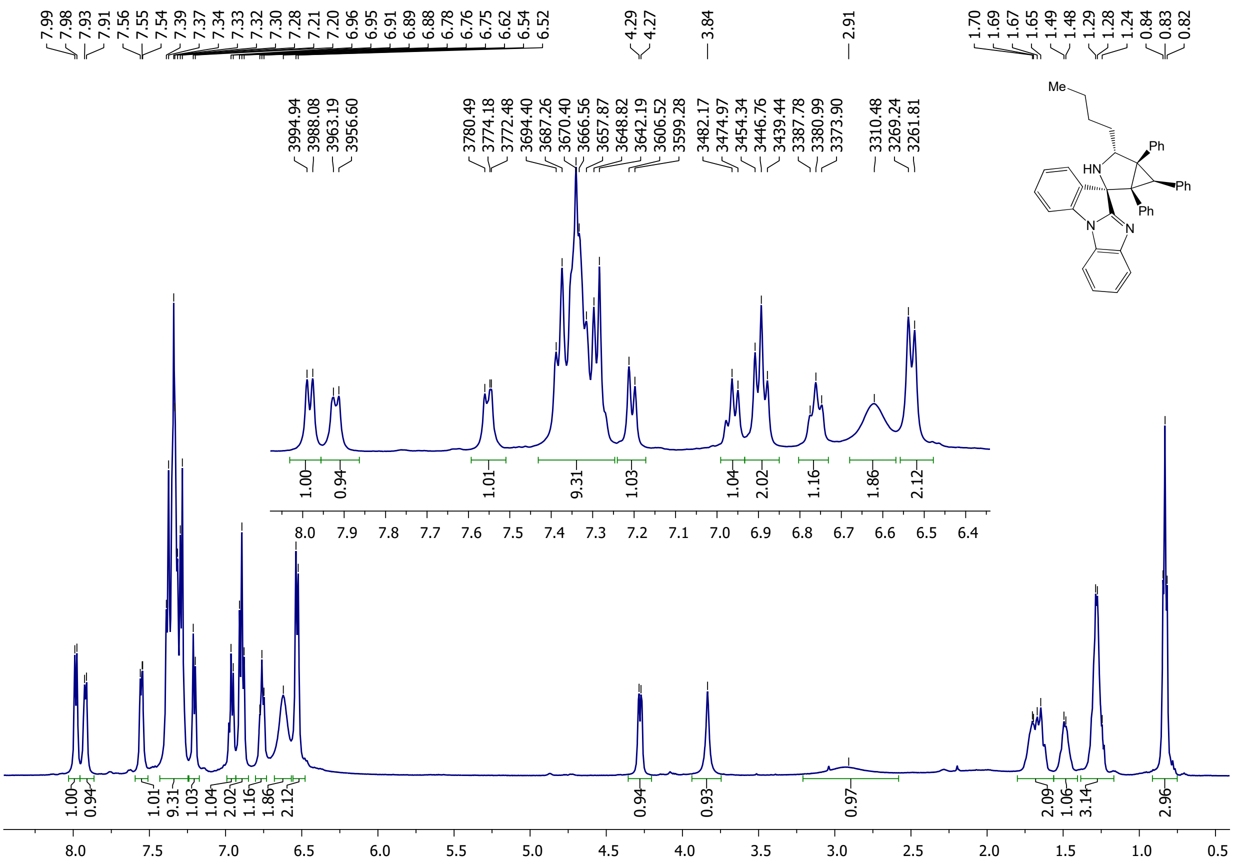

Figure S27. ^{1}H NMR spectrum of compound **6d** ($CDCl_3$, 400 MHz)

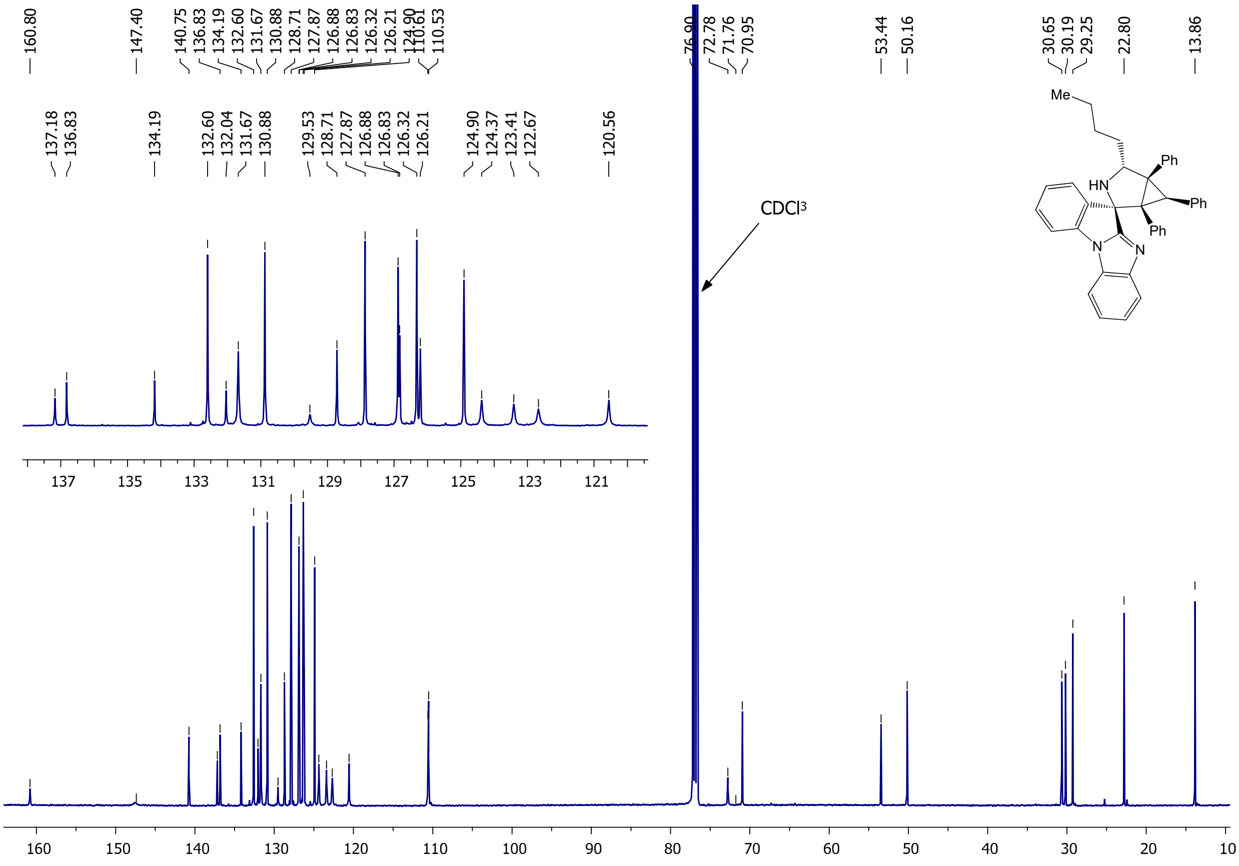

Figure S28. ^{13}C NMR spectrum of compound **6d** ($CDCl_3$, 400 MHz)

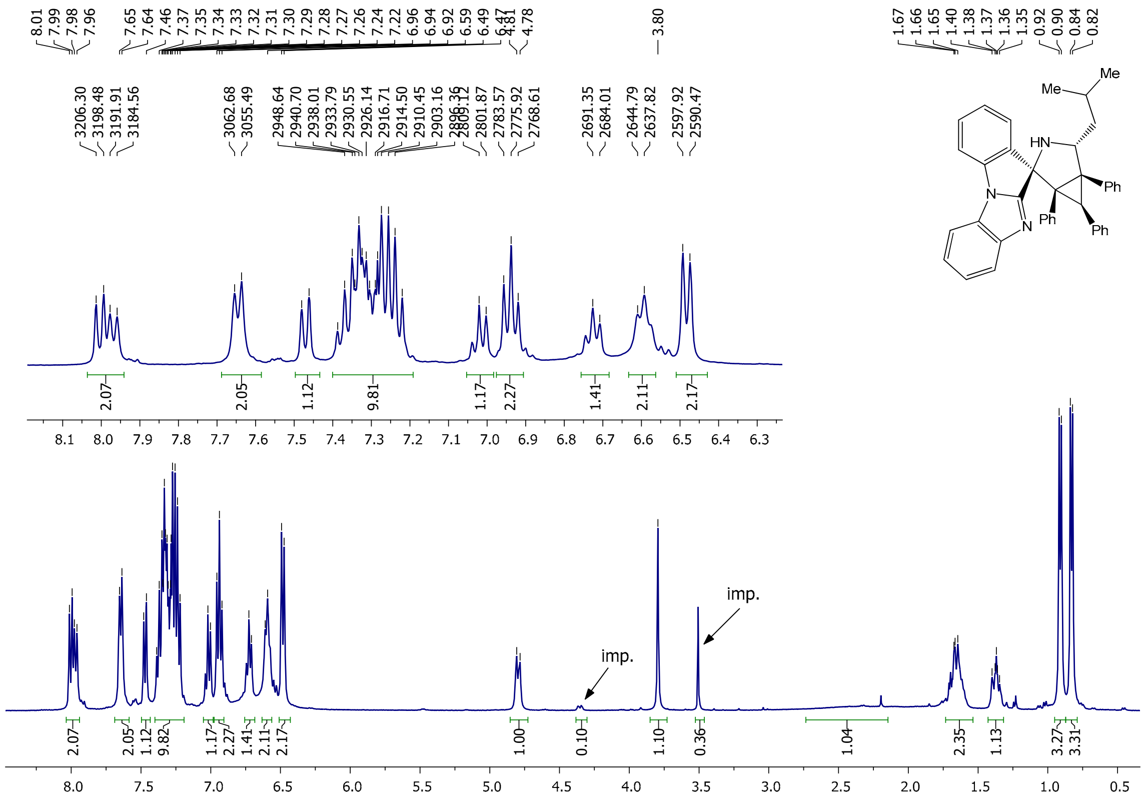

Figure S29. ^{1}H NMR spectrum of compound **6e** ($CDCl_3$, 400 MHz)

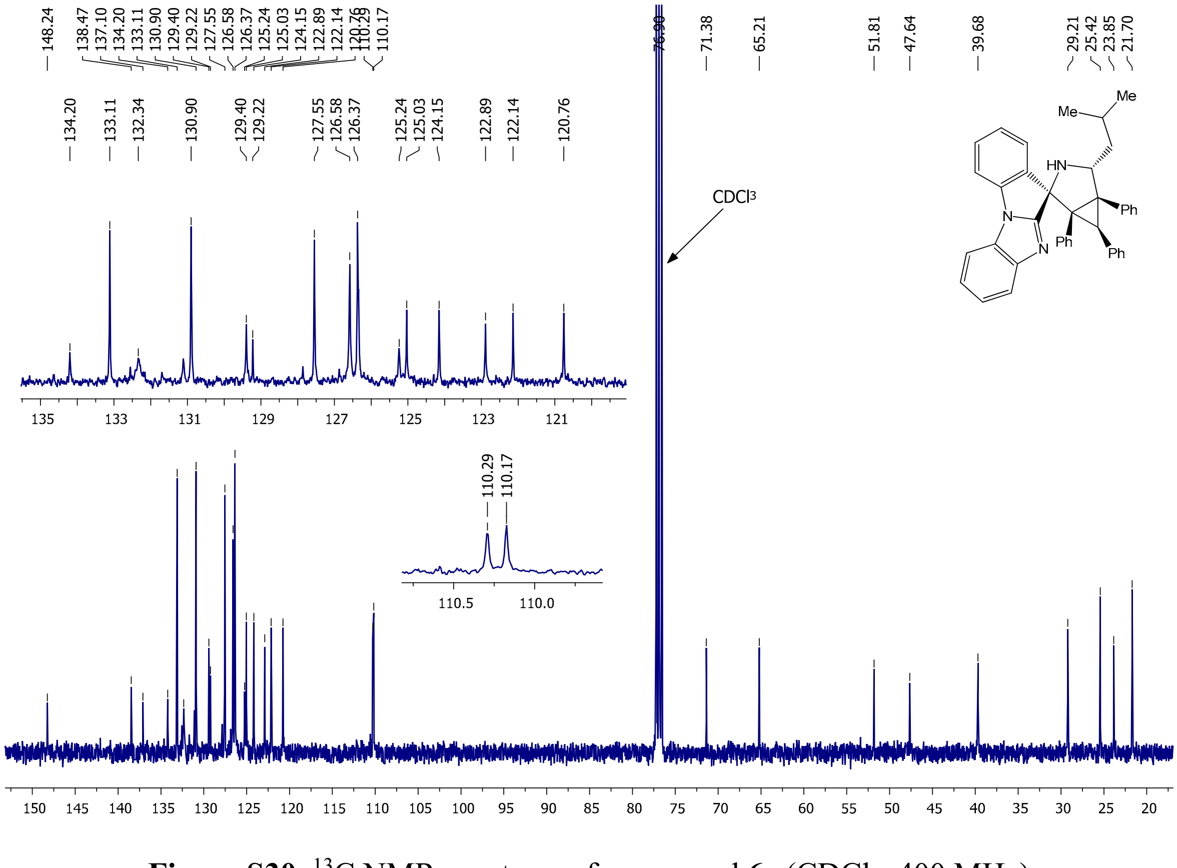

Figure S30. ^{13}C NMR spectrum of compound **6e** ($CDCl_3$, 400 MHz)

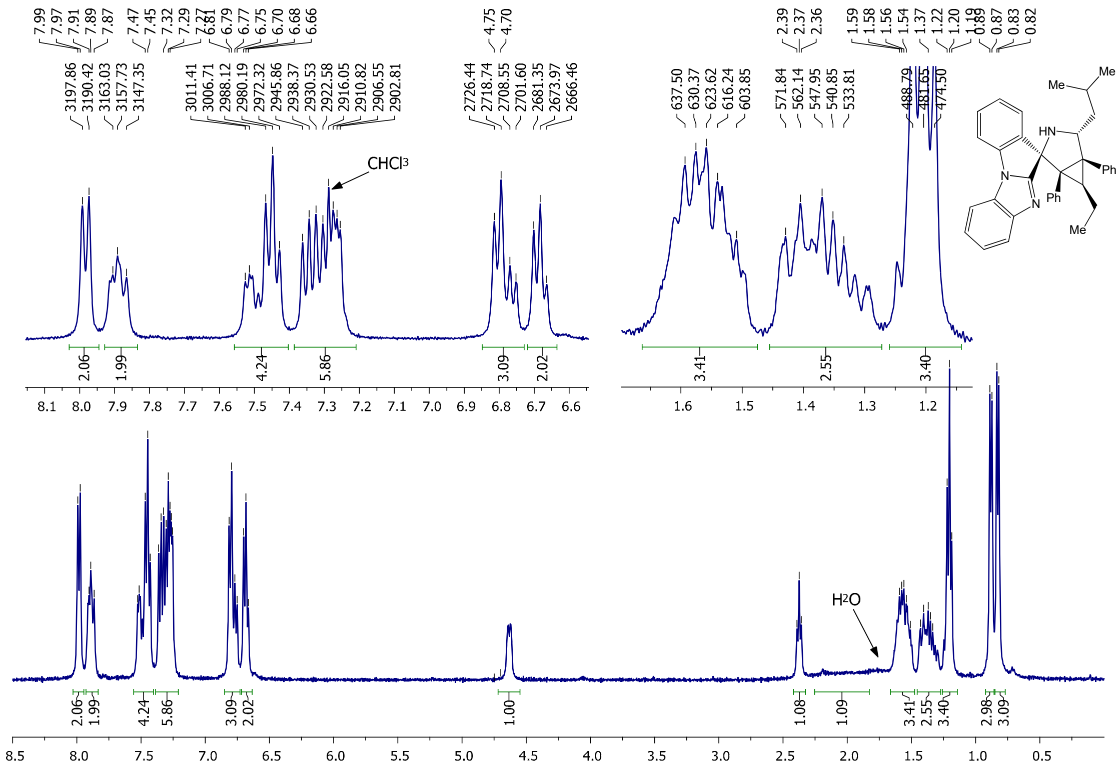

Figure S31. ^{1}H NMR spectrum of compound **6f** ($CDCl_3$, 400 MHz)

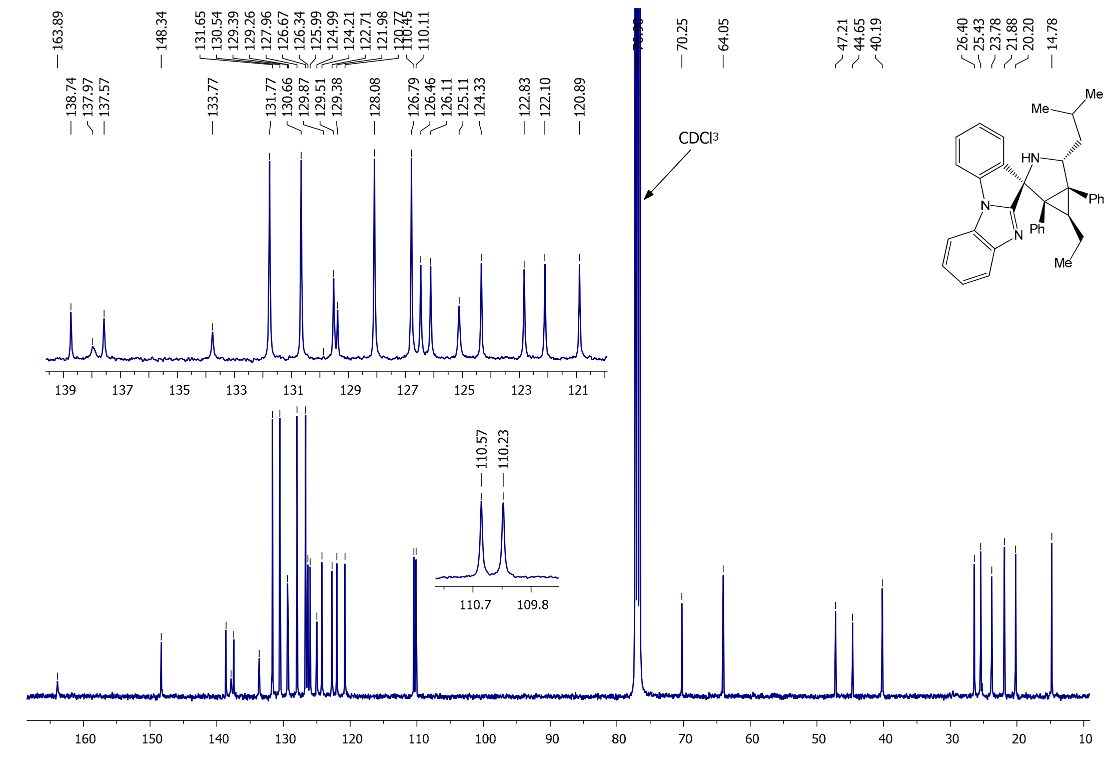

Figure S32. ^{13}C NMR spectrum of compound **6f** ($CDCl_3$, 101 MHz)

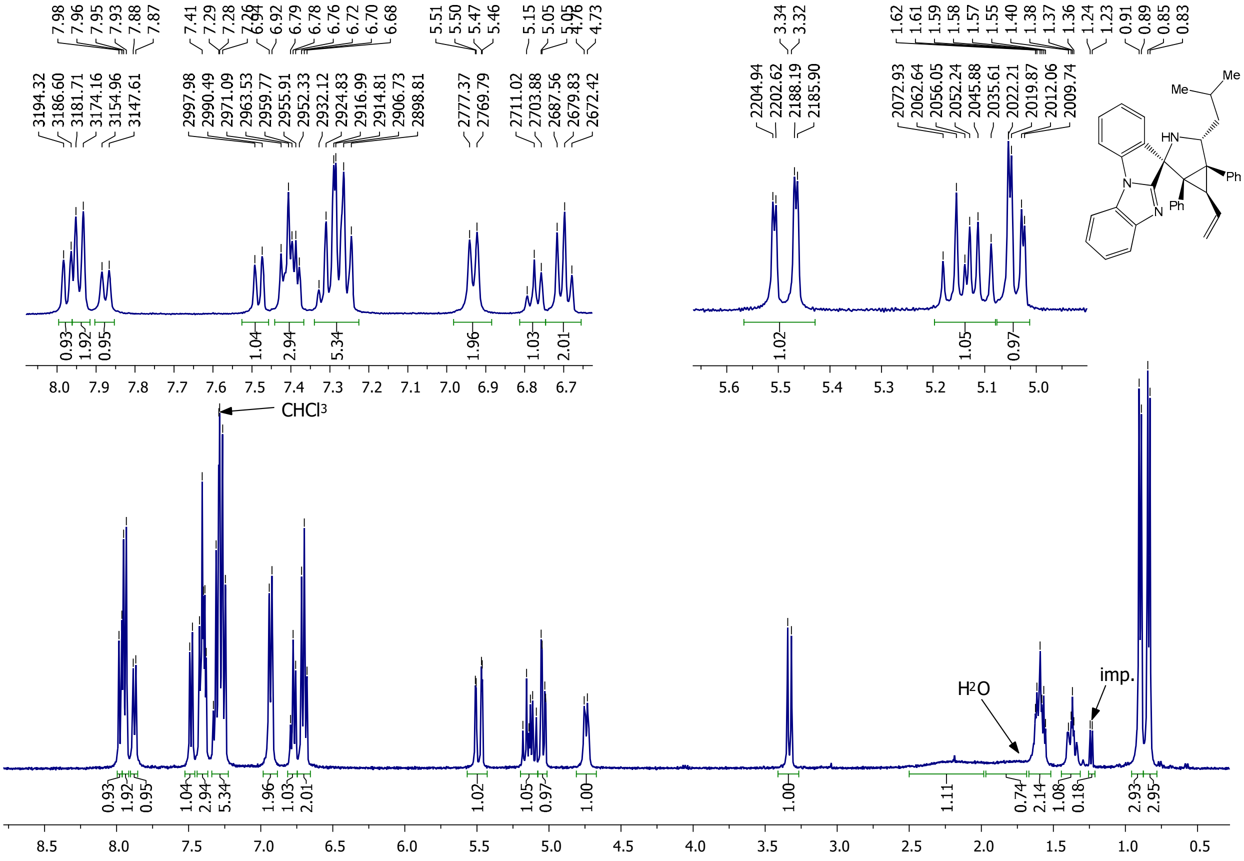

Figure S33. ^{1}H NMR spectrum of compound **6g** ($CDCl_3$, 400 MHz)

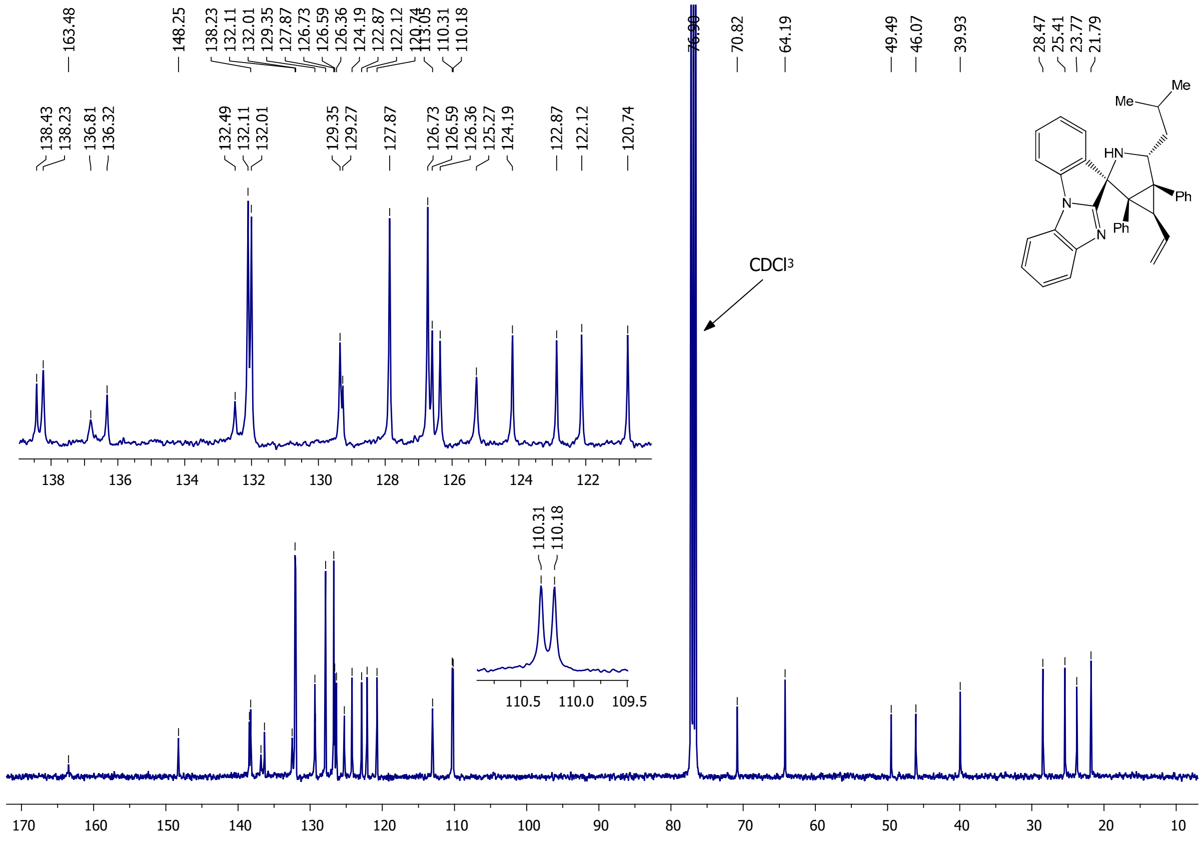

Figure S34. ^{13}C NMR spectrum of compound **6g** ($CDCl_3$, 101 MHz)

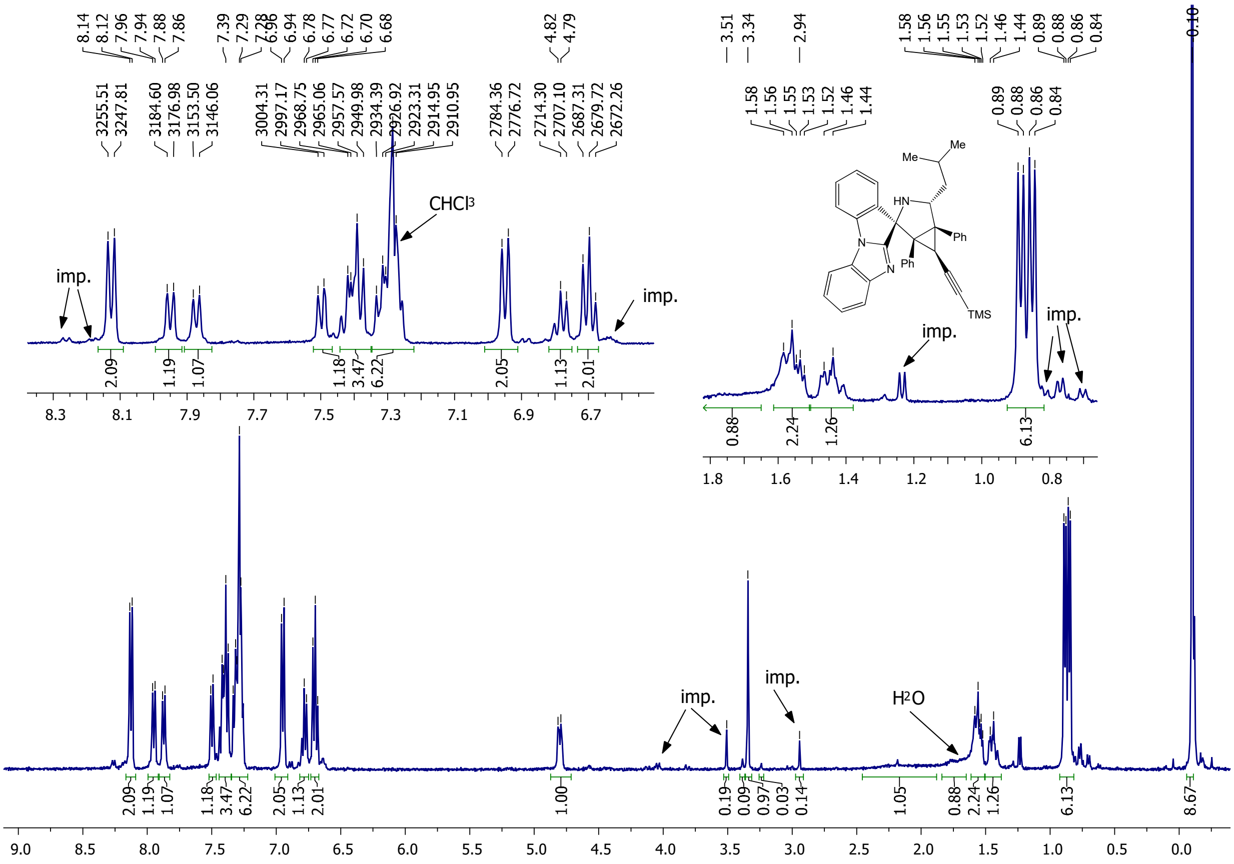

Figure S35. ^{1}H NMR spectrum of compound **6h** ($CDCl_3$, 400 MHz)

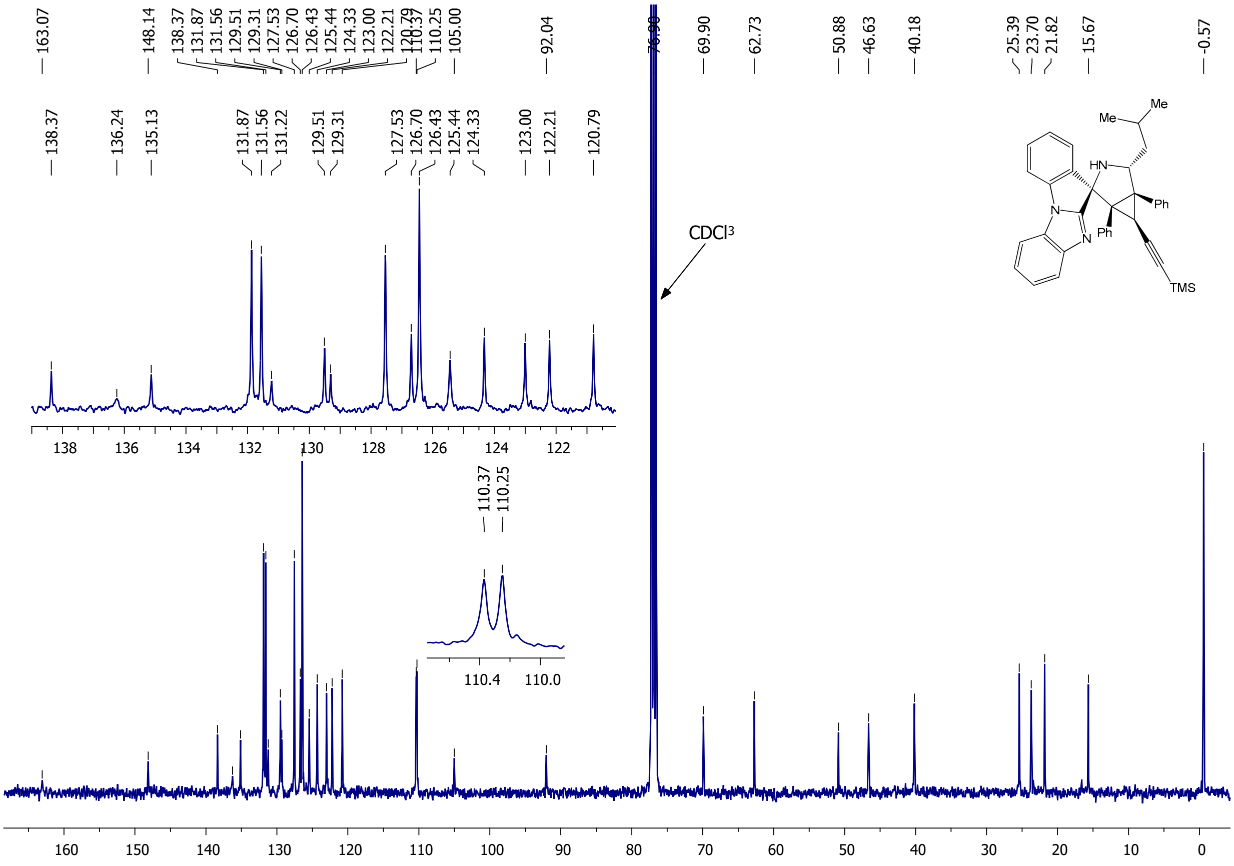

Figure S36. ^{13}C NMR spectrum of compound **6h** ($CDCl_3$, 101 MHz)

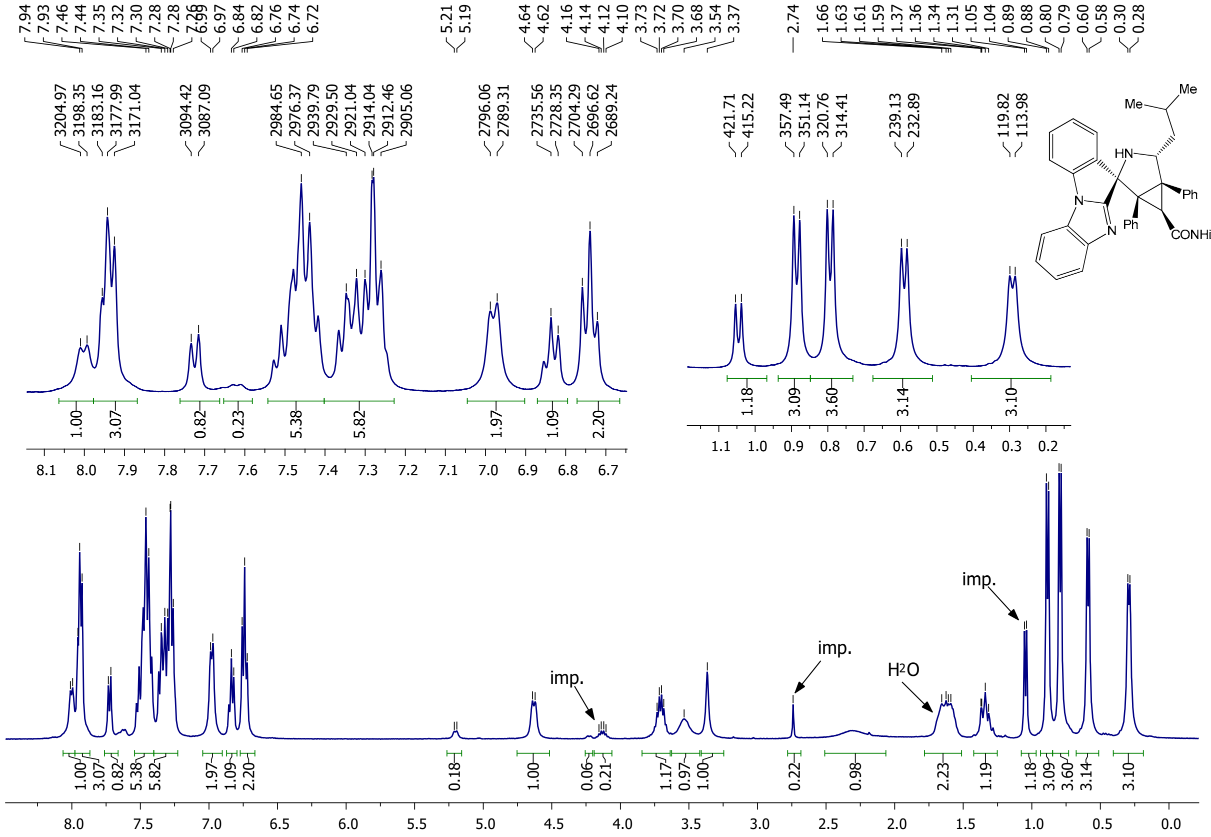

Figure S37. ^{1}H NMR spectrum of compound **6i** ($CDCl_3$, 400 MHz)

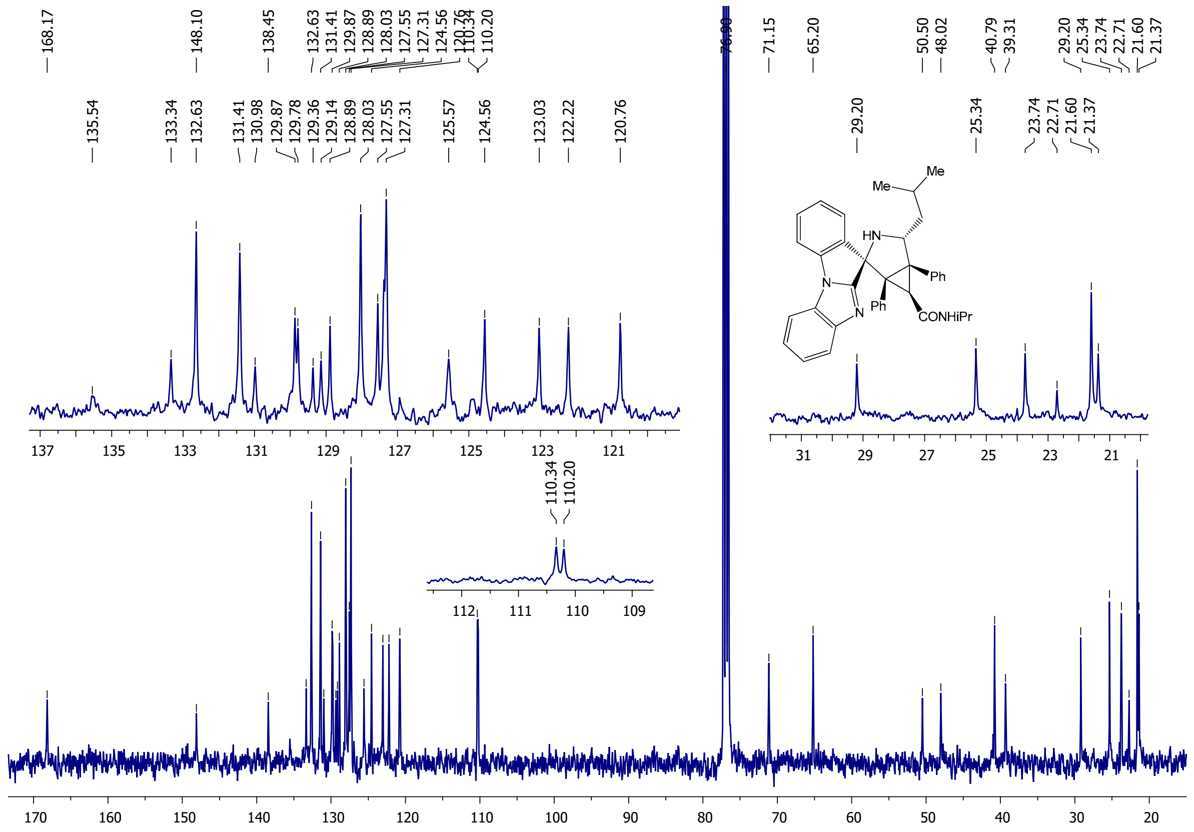

Figure S38. ^{13}C NMR spectrum of compound **6i** ($CDCl_3$, 101 MHz)

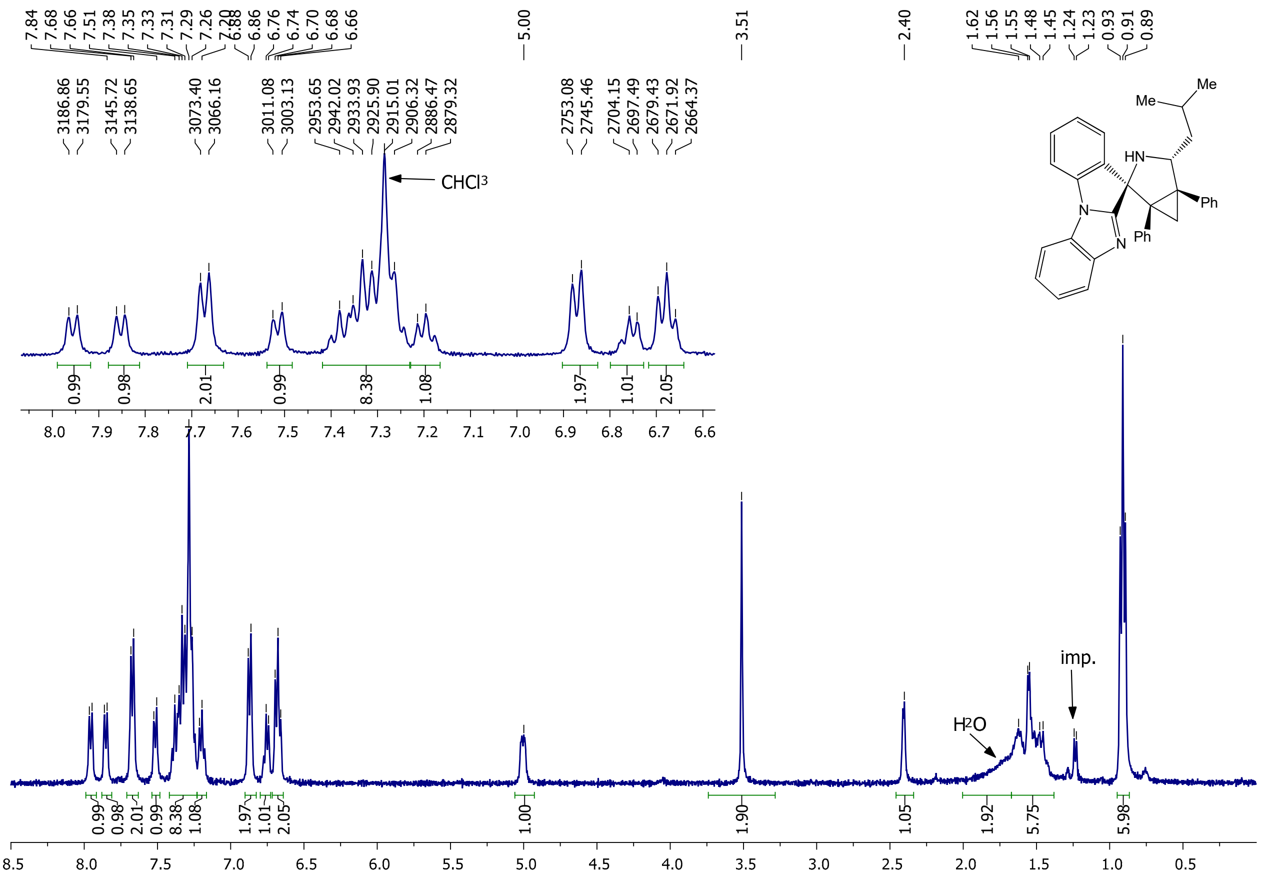

Figure S39. ^{1}H NMR spectrum of compound **6j** ($CDCl_3$, 400 MHz)

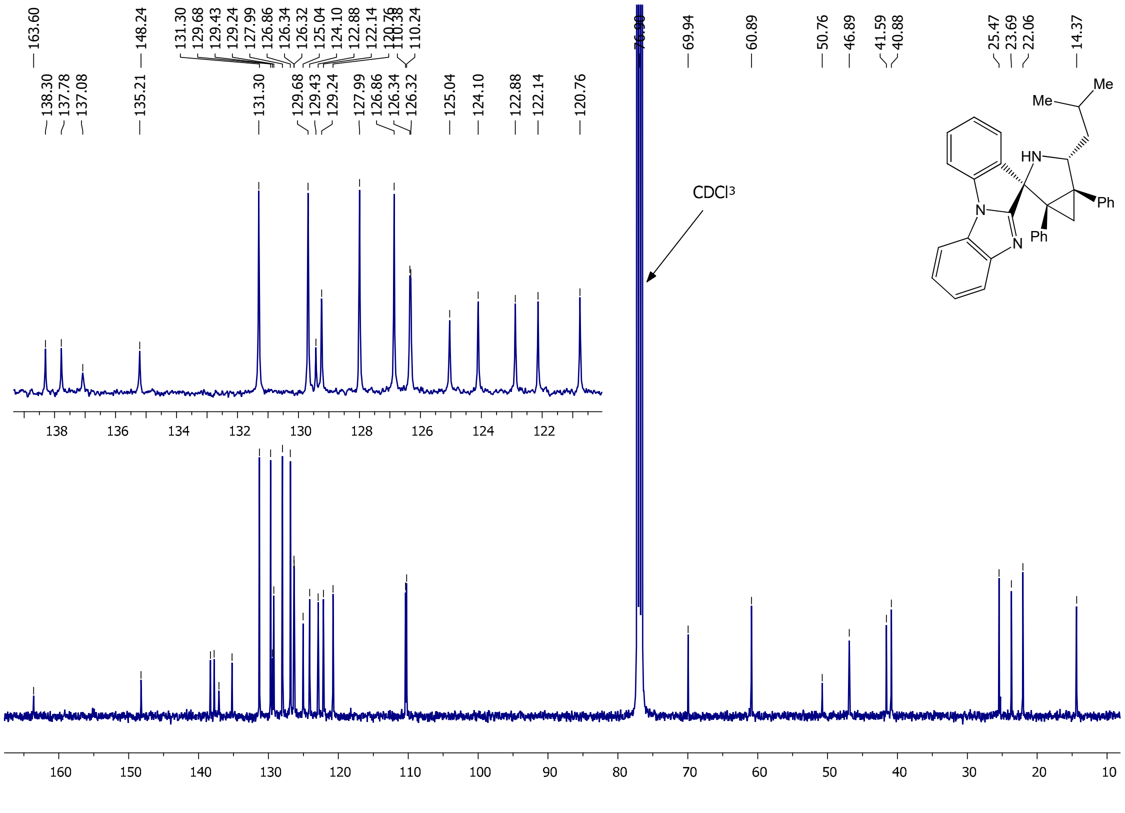

Figure S40. ^{13}C NMR spectrum of compound **6j** ($CDCl_3$, 101 MHz)

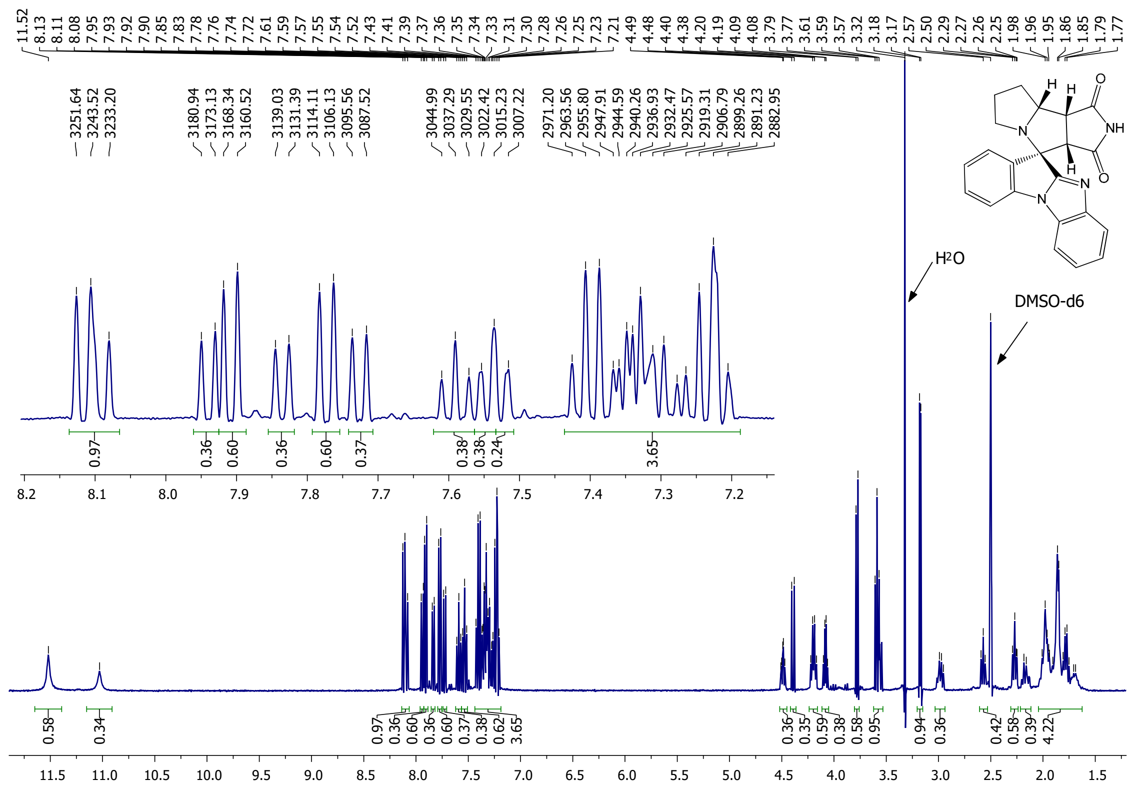

Figure S41. ^{1}H NMR spectrum of compound **8a**, 1.5:1 *dr* (DMSO-d_6, 400 MHz)

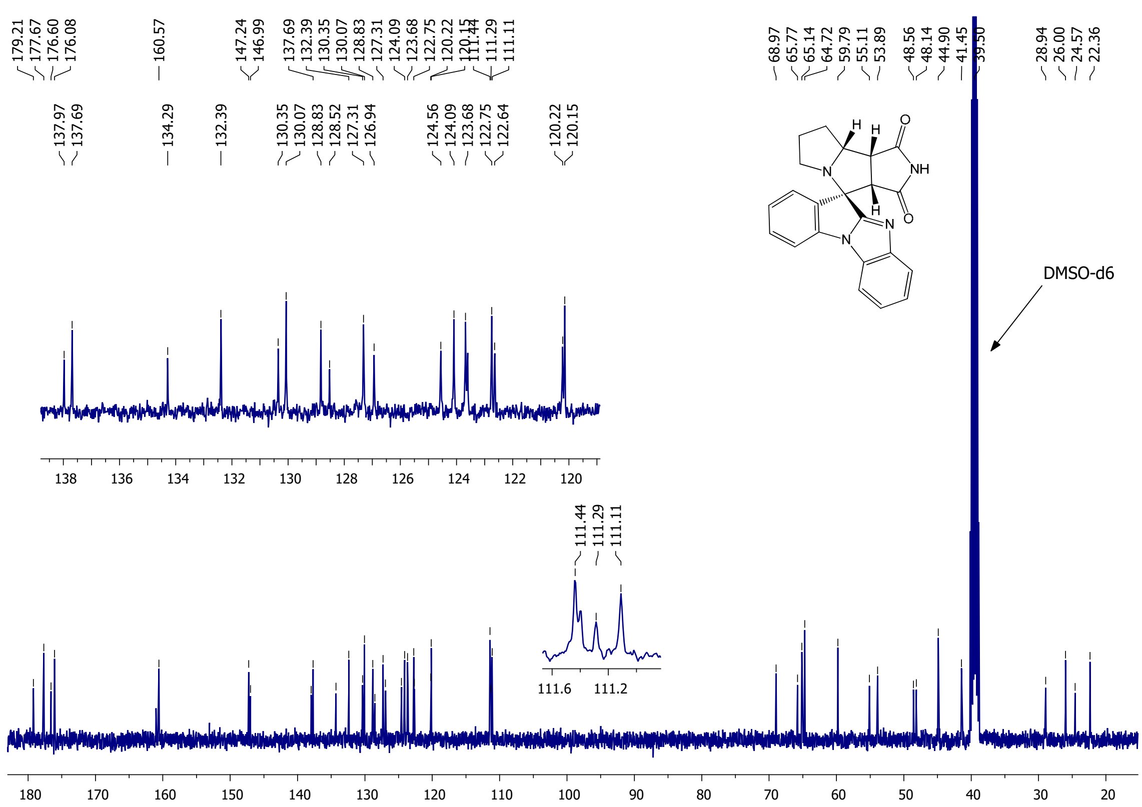

Figure S42. ^{13}C NMR spectrum of compound **8a**, 1.5:1 *dr* (DMSO-d_6, 101 MHz)

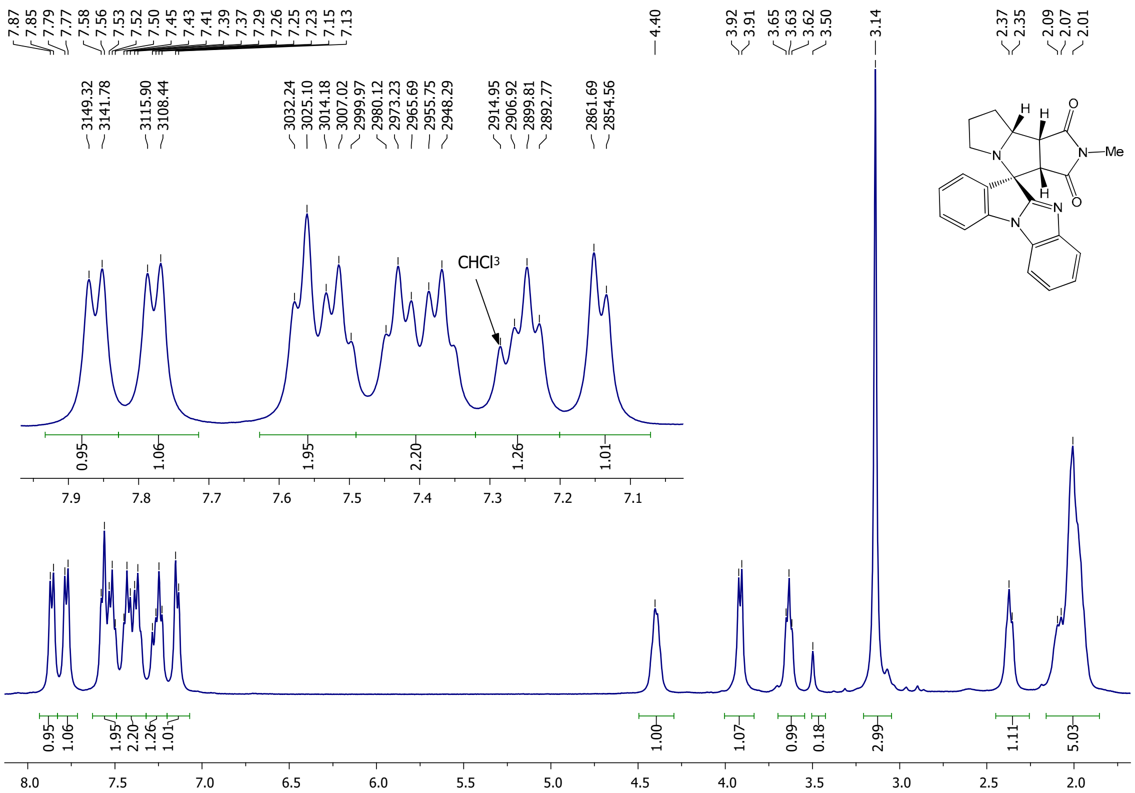

Figure S43. ^{1}H NMR spectrum of compound **8b**, 1.7:1 *dr* ($CDCl_3$, 400 MHz)

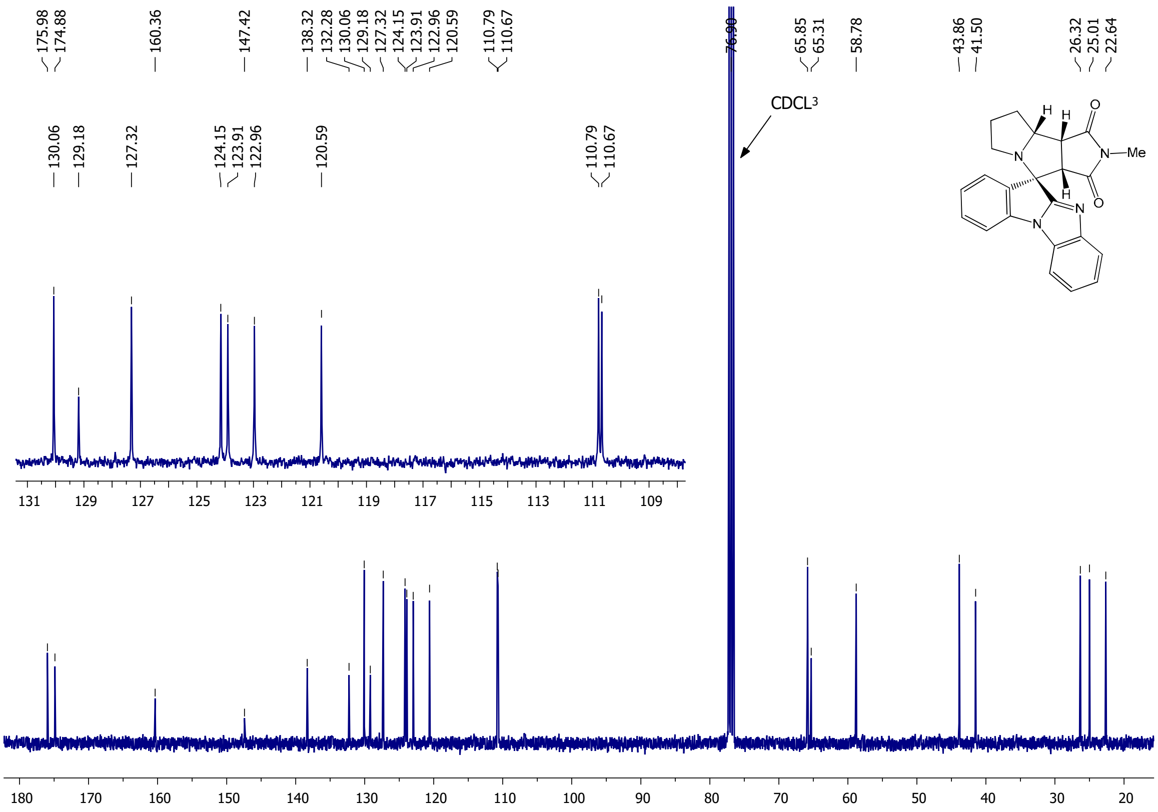

Figure S44. ^{13}C NMR spectrum of compound **8b**, 1.7:1 *dr* ($CDCl_3$, 101 MHz)

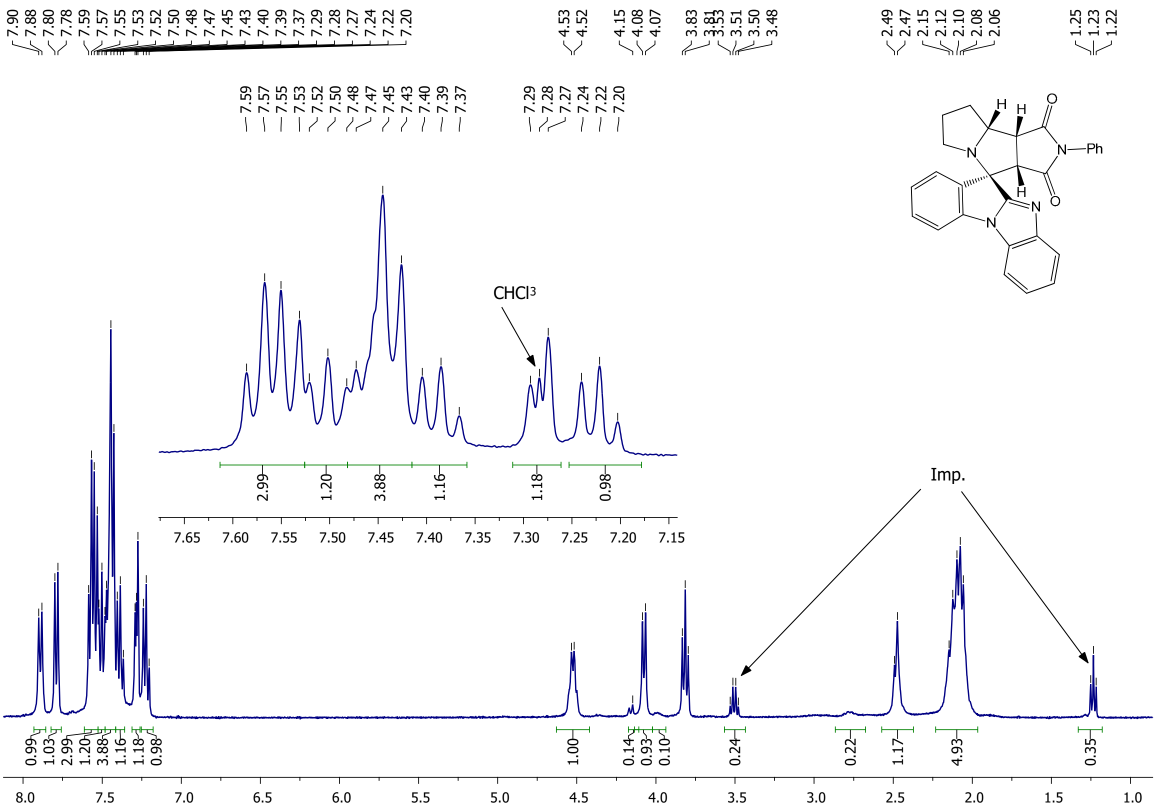

Figure S45. ^{1}H NMR spectrum of compound **8c**, 9:1 *dr* ($CDCl_3$, 400 MHz)

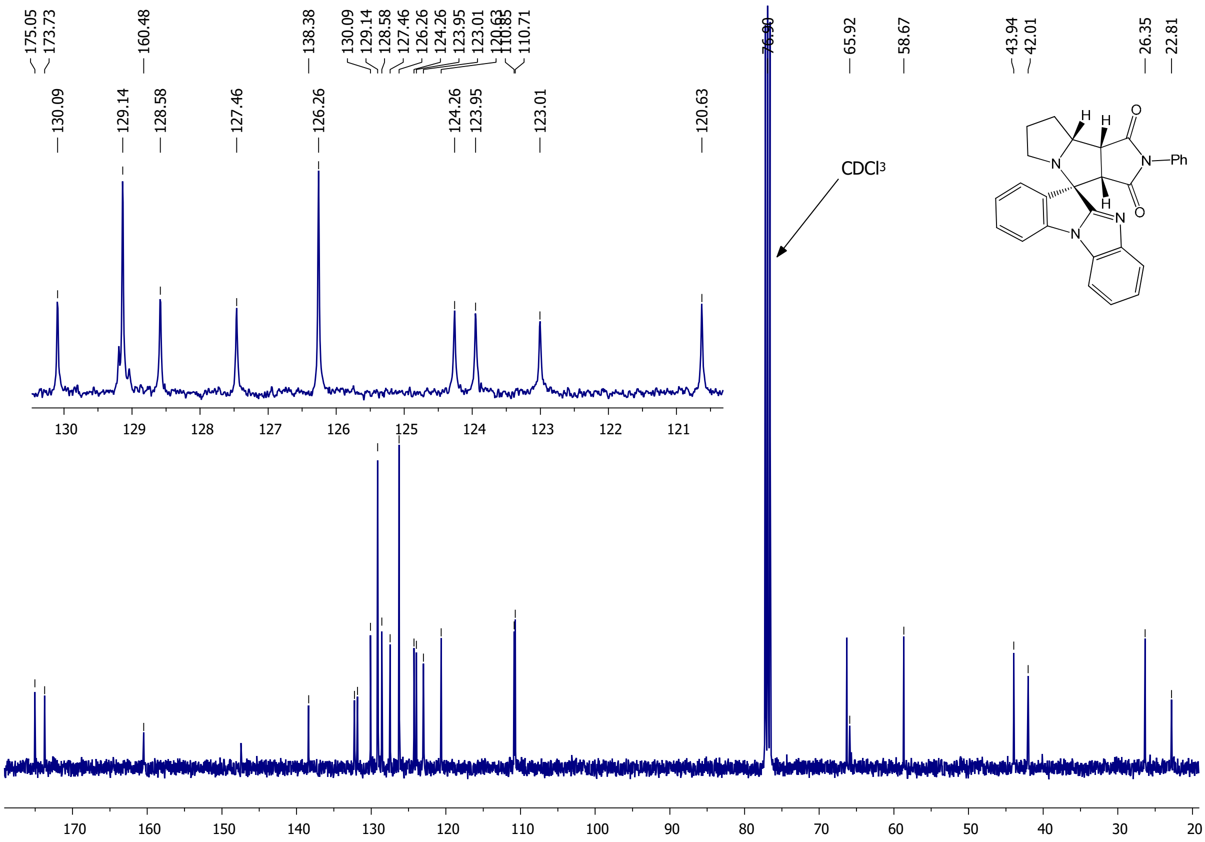

Figure S46. ^{13}C NMR spectrum of compound **8c**, 9:1 *dr* ($CDCl_3$, 101 MHz)

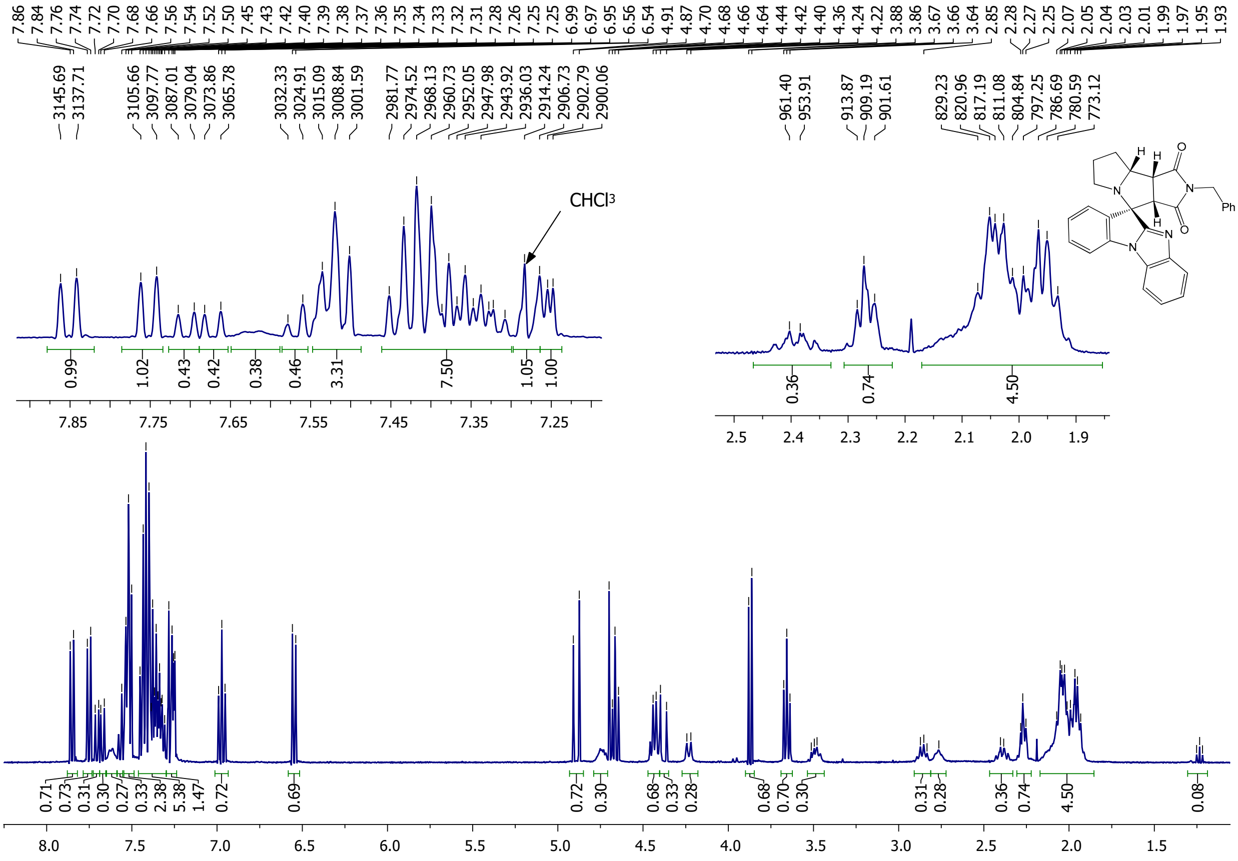

Figure S47. ^{1}H NMR spectrum of compound **8d**, 2.3:1 *dr* ($CDCl_3$, 400 MHz)

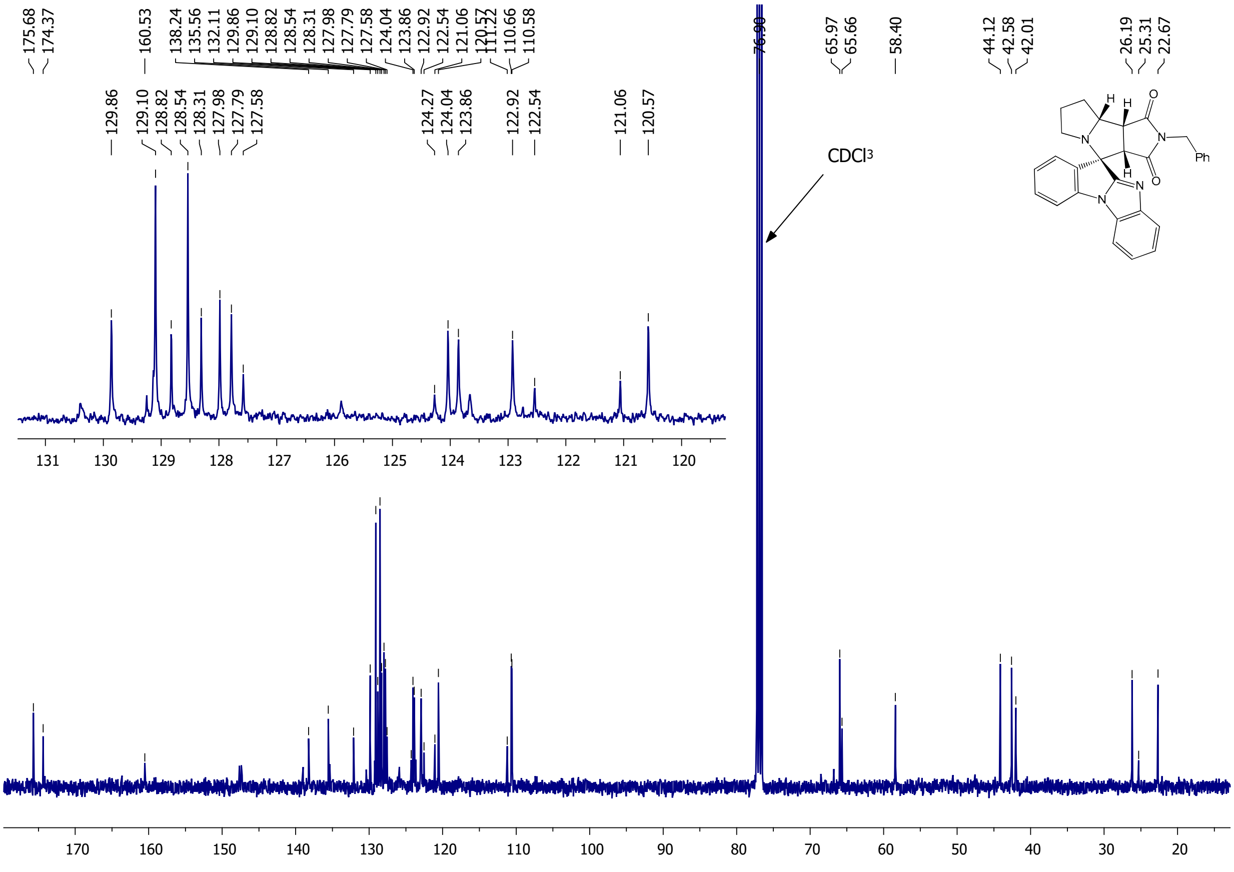

Figure S48. ^{13}C NMR spectrum of compound **8d**, 2.3:1 *dr* ($CDCl_3$, 101 MHz)

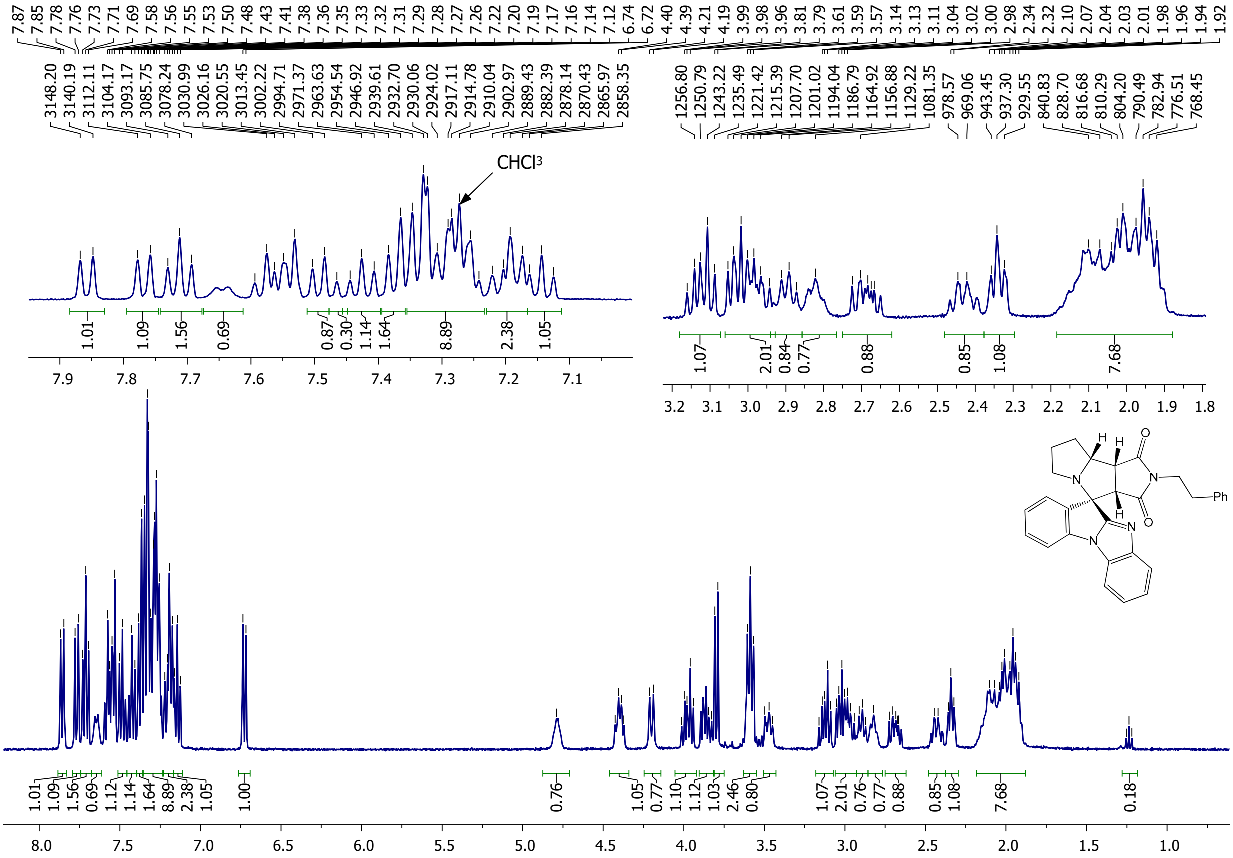

Figure S49. ^{1}H NMR spectrum of compound **8e**, 1.5:1 *dr* ($CDCl_3$, 400 MHz)

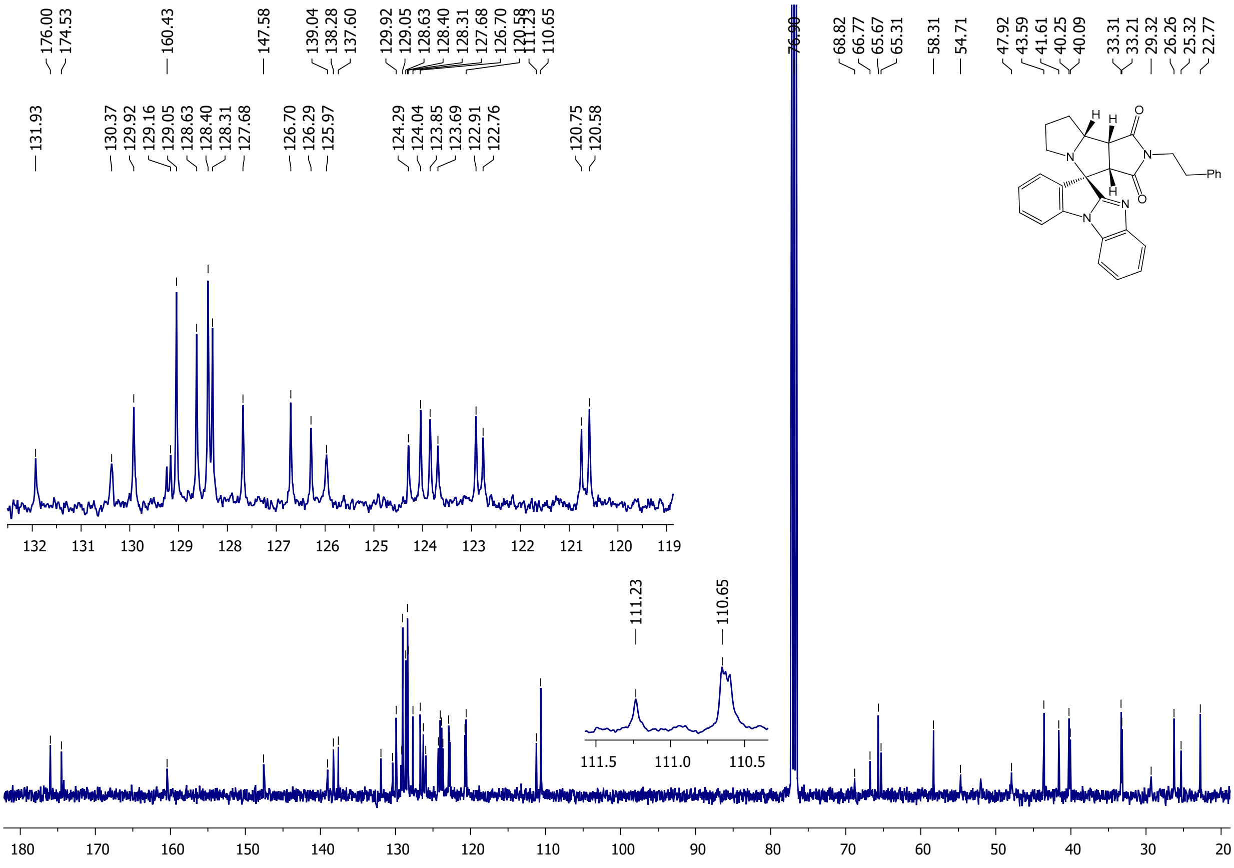

Figure S50. ^{13}C NMR spectrum of compound **8e**, 1.5:1 *dr* ($CDCl_3$, 101 MHz)

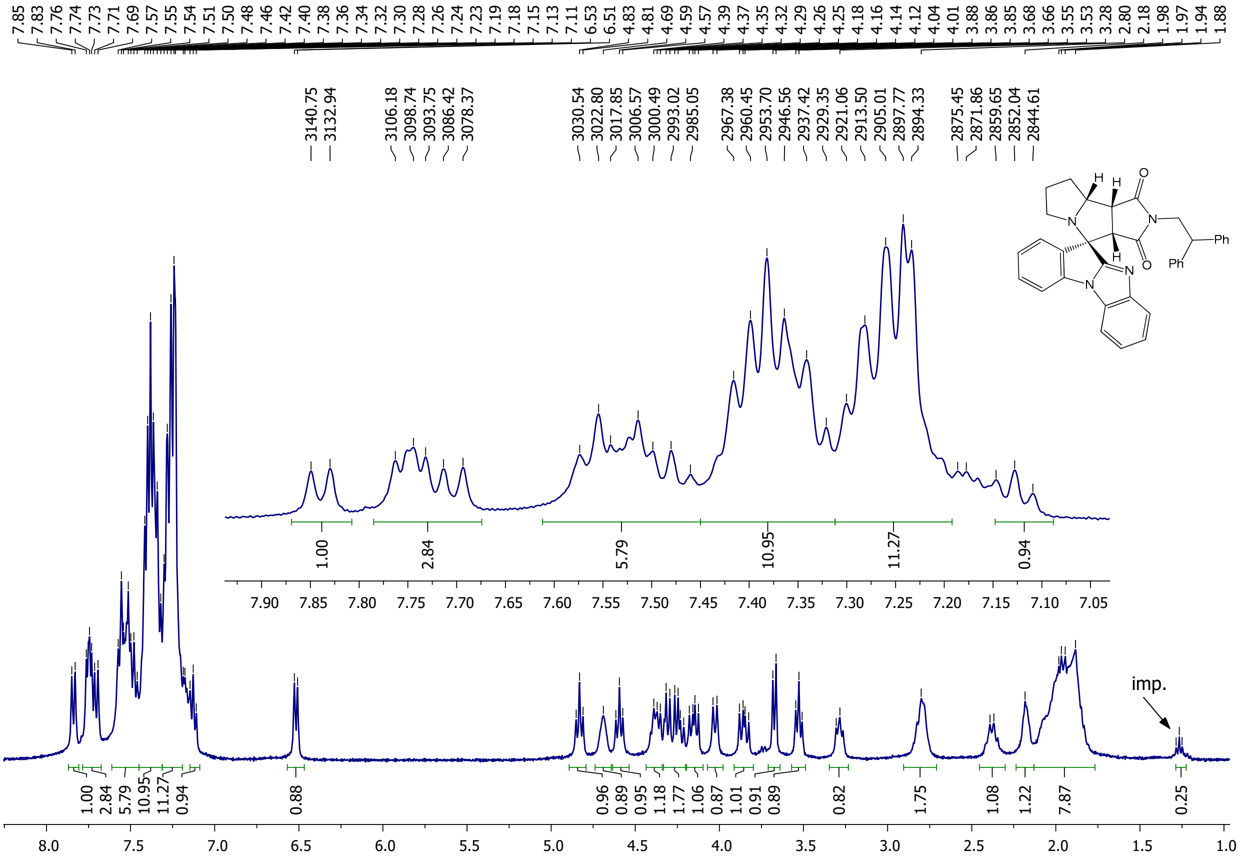

Figure S51 ^{1}H NMR spectrum of compound **8f**, 1.2:1 *dr* ($CDCl_3$, 400 MHz)

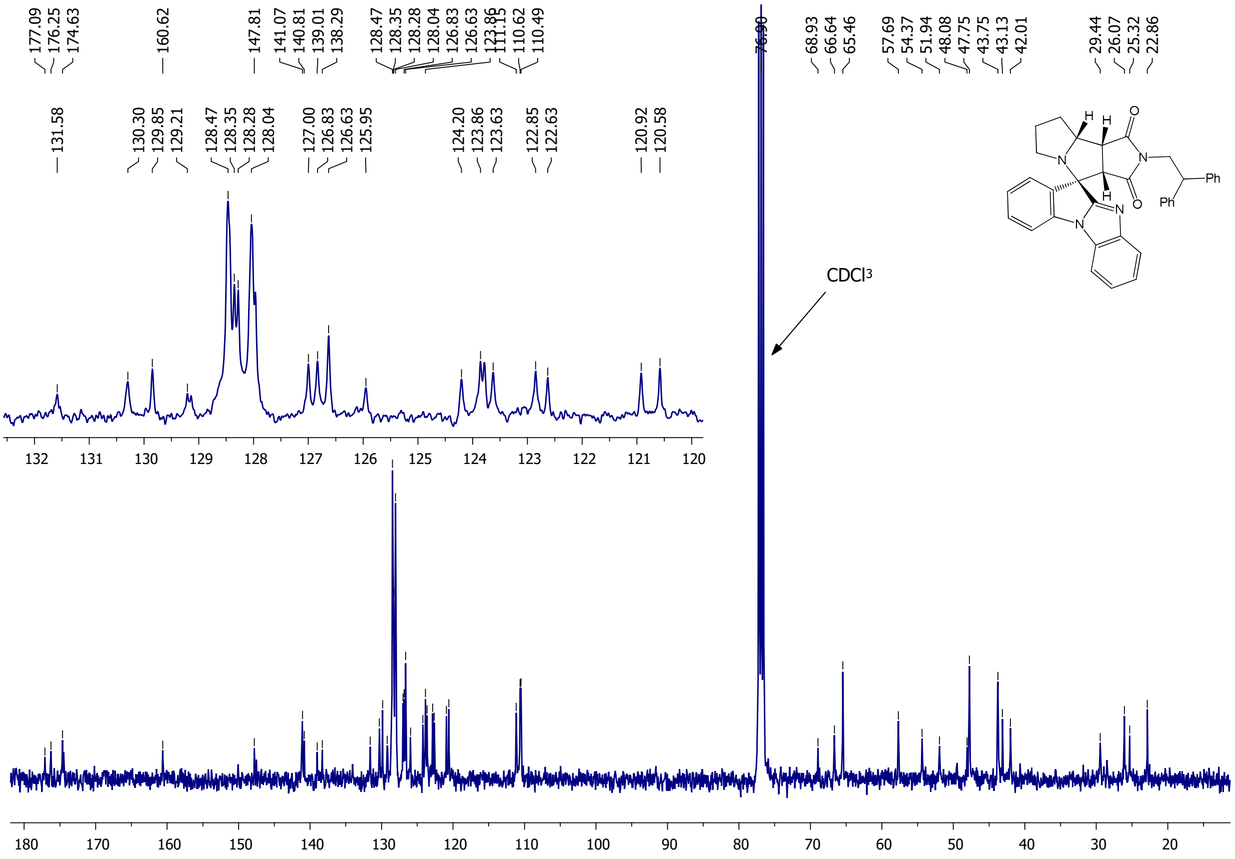

Figure S52 ^{13}C NMR spectrum of compound **8f**, 1.2:1 *dr* ($CDCl_3$, 101 MHz)

2. The 2D NMR analysis of the compounds 4d and 5d

The three-component 1,3-dipolar cycloaddition reaction between **1d**, **2** and **3a** led to two products **4d** and **5d**, which were in the reaction mixture in a ratio 6 : 1 and were isolated and studied individually using NMR spectroscopy methods. The spatial structure of the major product **4d** is shown in Fig. S53 and its proof was quite simply obtained from NOESY spectroscopy data after preliminary identification of all proton signals by COSY spectrum analysis (Fig. S54). The most typical for this compound are the following proton-proton spatial interactions (NOEs), which are shown in Fig. S53 using red asterisks, and the ratios of the integral intensities of the corresponding cross peaks, which were compared with the calculated values of the corresponding distances obtained by optimizing the geometry of the molecule **4d** by the MM2 molecular mechanics method.

It should be noted that the quantitative estimation of the experimental distances was carried out in the ***i***solated ***s***pin ***p***air ***a***pproximation (ISPA) [1] and relatively short mixing time ($\tau_m \approx 0.5$ s) was used. The values of the integral cross-peak intensities were reduced to the diagonal and so-called spherical calculation model [2,3] was implicated. The assumption of the absence (or insignificance of the contribution) of the anisotropy of the total rotational diffusion in the solution of the studied molecules **4d** and **5d** is based on the relatively low (about 2) calculated value of the diffusion coefficients around the principal axes $D_{\parallel}/D_{\perp}$ [4,5]. Moreover, when analyzing the distances between protons in the rigid part of the molecule (H6a`, H1`) and degenerate *ortho*-protons of mobile aromatic rings (H12,16 and H18,22), only the distance to the nearest of the two protons was taken into account [6-8], since, according to calculations, the distance to the farthest *ortho*-proton was significantly (by 1.5 – 2.0 Å) longer than that of the nearest one, and the contribution of distant protons to the total NOE was a negligibly small value (no more than 2–3% of the measured value of the effective distance [9,10].

From the NOESY spectrum of compound **4d**, it follows (Fig. S53) that the most energetically favorable conformer (A) is characterized by proximity of proton pairs H6a`-H6`β ($r^{calc.}$ = 2.32 Å), H6a`-H12 (2.53 Å), H1`-H4`α (2.2 Å), H1`-H1 (2.4 Å), H4`α-H1 (2.16 Å) (footnote: here we do not take into account the distances between the geminal protons of the aliphatic ring, which are 1.78 Å and have the most intense cross peaks in the NOESY spectrum and can only be used to confirm the identification of these protons) and sufficient (up to 4.0 Å) distance of proton pairs H6a`-H6`α (3.0 Å), H6a`-H5`β (3.14 Å), H6a`- H4`β (3.9 Å), H6a`-H18 (3.9 Å), H6a`-H1` (3.87 Å), H1`-H16 (3.56 Å), H1`-H22 (3.41 Å). Almost all of the distances listed above correspond to cross peaks in the NOESY spectrum, the intensities of which fully (i.e. at the experimental level - $r^{exp.}$) reflect) calculated (MM2) distance values ($r^{calc.}$) indicated in parentheses and/or by figures in Fig. S53.

For example, the distance between geminal protons (1.8 Å) corresponds to a cross peak with a relative intensity of about 18%, while the 1`/1 cross peak is 7.2%. Therefore, the experimental value of this distance is $r_{1`\text{-}1}^{(exp.)} = 1.8(16{:}7.2)^{1/6} \approx 2.15$ Å, which is slightly less (by 0.25 Å) than the calculated value of 2.4 Å. At the same time, the relative intensity of the 1/4`α cross peak is 2.4%, and this value corresponds to the experimental distance $r_{1\text{-}4\alpha}^{(exp.)}$ = 2.56 Å, while its calculated value (2.14 Å) is 0.42 Å less. A similar situation is observed for the direct dipole-dipole interaction between protons H1` and H4`α: $r_{1`\text{-}4`\alpha}^{(exp.)}$ = 2.58 Å turns out to be greater than $r_{1`\text{-}4`\alpha}^{(calc.)}$ = 2.2 Å by 0.38 Å. Finally, the overestimated value of the cross-peak

between the signals of the H6a` and H4`β protons, which is 2%, is completely unexpected, since the calculated value of the distance $r_{6a`-4`\beta}^{(calc.)}$ = 3.91 Å and the intensity of the indicated cross-peak should not exceed 0.3%.)

The above discrepancies between the calculated and experimental values of the interproton distances give grounds for assuming the existence of the **4d** molecule in solution in the form of a fast exchange on the NMR time scale between two (or more) conformations, which differ from the energetically more preferable conformation **(A)** of compound **4d** by the proximity of H6a` protons and H4`β and the distance from each other of the protons H1 and H4`α. Thus, in experiments on the measurement of NOE, its time-averaged value is observed, which leads to a deviation from the calculated values of the distances for each of the conformers, if they differ significantly from each other and the proximity of protons is characteristic of a minor conformer [5-7].

The existence of a fast conformational exchange of compound **4d** is indirectly confirmed by the dynamic broadening (up to 8.5 Hz) of individual carbon signals in the aliphatic region of the ^{13}C NMR spectrum (Fig. S55, a), and the signals of the H4`α protons (2.86 ppm) and H4`β (2.38 ppm) in the ^{1}H spectrum have the same (doublet of triplets)) multiplet structure, the sum of the scalar constants of which is 20.2 and 20.7 Hz, respectively (Fig. S55, b). At the same time, the width of the components of the second of these multiplets (H6`β) turns out to be 0.4 Hz larger, which can also be associated with the dynamic process of inversion of the nitrogen atom N3` in the **4d** molecule, which is fast on the NMR time scale (Fig. S53).

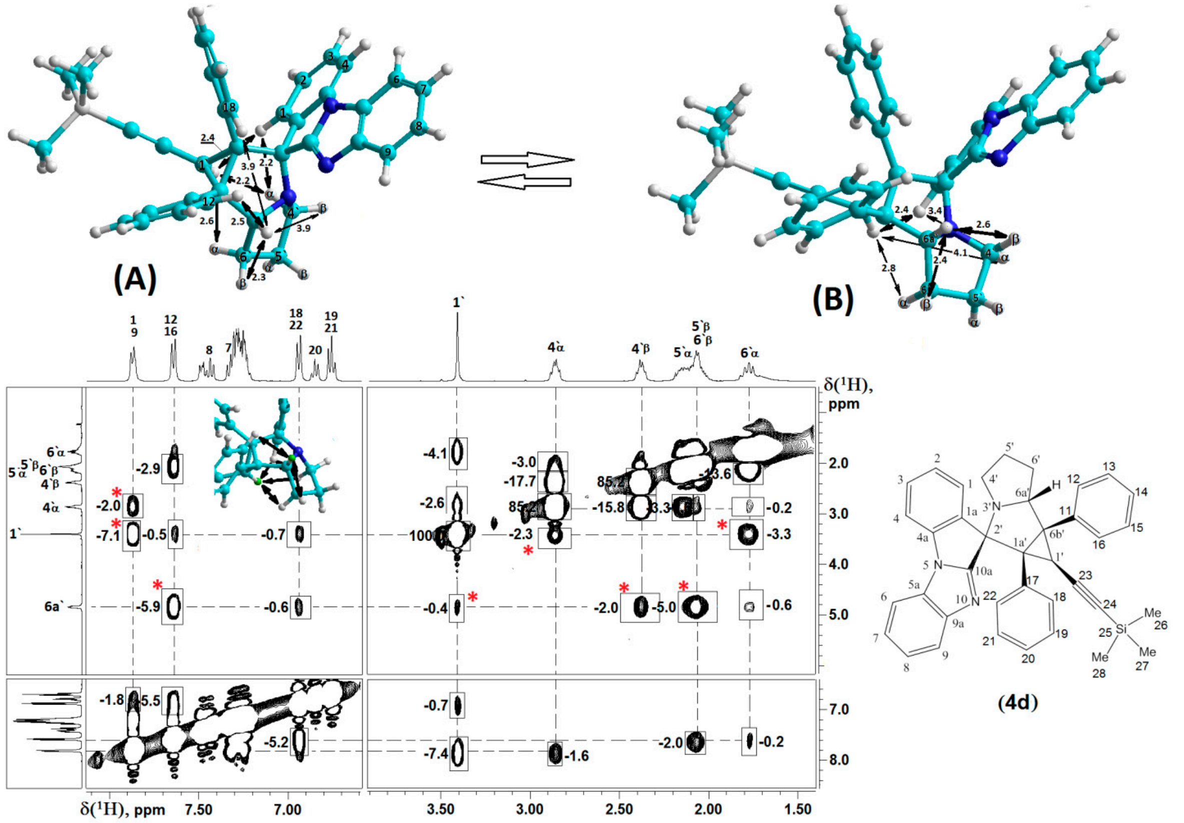

Fig. S53 The interproton distances most important for proving the structure and their corresponding cross peaks are shown by double arrows and rectangles on the 3D model of the molecule and on the spectrum, respectively. The calculated (MM2) distance values are given by figures in angstroms (Å), and the volume integrals are given in % relatively to the intensity of the diagonal signal of the proton H1`.

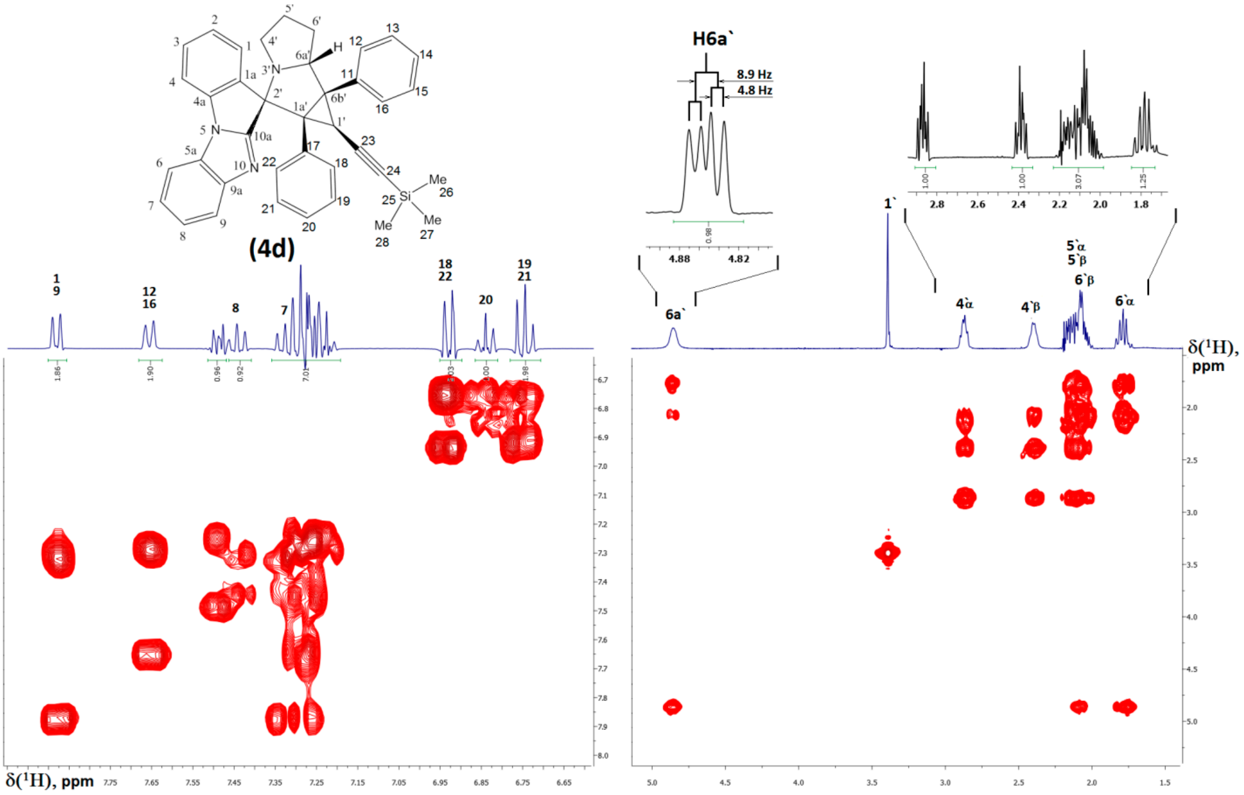

Fig. S54 Fragments of COSY spectrum of the major diastereomer **4d**. ^{1}H spectrum is present at different Lorentz-Gauss window functions: blue color at LB = -1 Hz, GB = 3 Hz; black color at LB = -4 Hz, GB = 2.2 Hz.

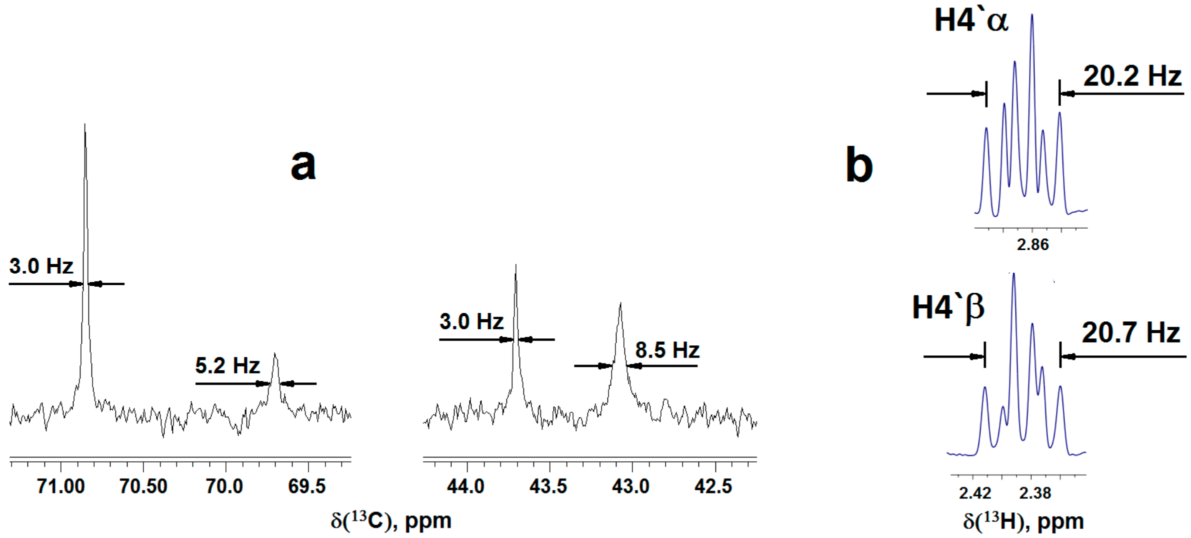

Fig. S55 Fragments of ^{13}C (**a**) and ^{1}H (**b**) NMR spectra of the compound **4d**. Dynamic effects of fast in NMR scale conformational exchange are shown by broadening of some carbon and proton signals.

However, calculations by the MM2 method indicate the possibility of the existence of at least one more minor conformer (**B**) and, therefore, the dynamic exchange of compound **4d** is more complicated (Fig. S56).

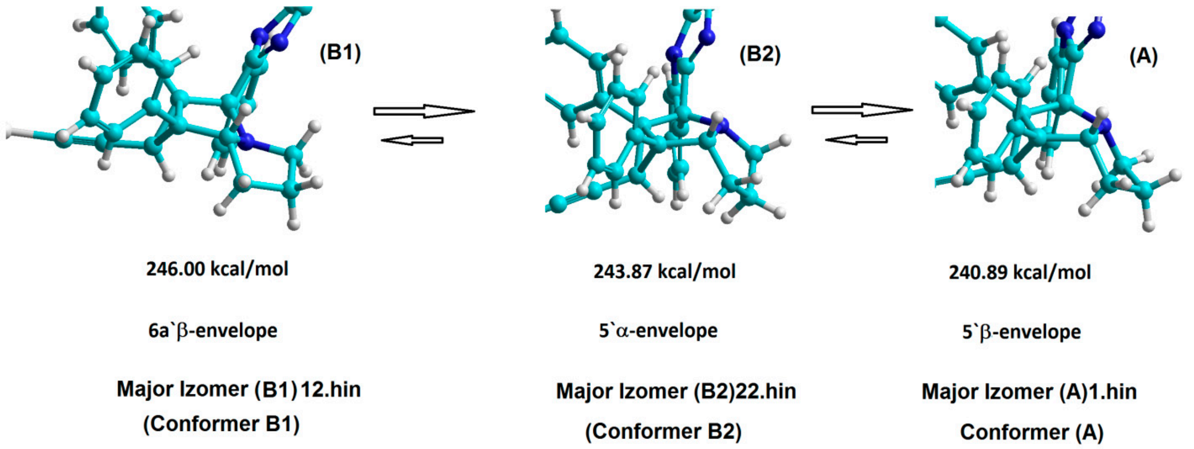

Fig. S56 Conformational exchange of the compound **4d**. (Calculated values of energy, type of 5-membered ring and HyperChem file-name of conformers are given by text)

The structure of the minor product **5d** was established in a similar way, taking into account the results obtained for the dominant product **4d**. Comparison of the ^{1}H NMR spectra of these products indicates to significant changes in the chemical shifts in the spectrum of compound **5d** compared to **4d**, which are observed in both its aliphatic and aromatic parts (Fig. S57).

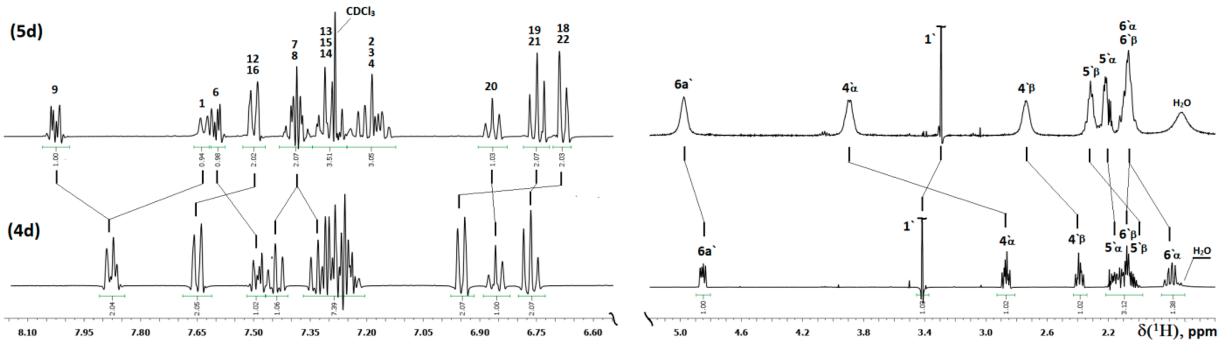

Fig. S57 Fragments of NMR ^{1}H spectrum of the compound **4d** and **5d**. The most significant differences in the proton chemical shifts in aromatic and aliphatic regions are shown by lines.

Almost all aliphatic protons of the compound **5d** (with the exception of the H1` proton signal) have higher chemical shifts than those of the compound **4d**. For example, for the H4`α proton signal, this increase exceeds 1.0 ppm. At the same time, for the doublet signals of *ortho*-protons of aromatic rings in positions 1a` and 6b` of compound **5d**, a decrease in chemical shifts by 0.25 and 0.14 ppm, respectively, is observed, and for the H1 proton, this decrease is 0.24 ppm.)

It should be also mentioned that there is a noticeable broadening of the signals of aliphatic protons, as well as a broadening of the doublet signal of the H1 proton at 7.61 ppm in the spectrum of compound **5d**, compared with the spectrum of compound **4d** (see Fig. S57). These broadenings may indicate the existence of a fast dynamic equilibrium in the 5-membered ring in the NMR time scale similar to that described above for **4d**, but occurring at a slower rate.

One of the possible variants of the structure of the minor compound **5d** is the 6a' diastereomer, which differs from the dominant compound **4d** by the opposite orientation (i.e., α-,

not β-) of the proton in position 6a`. This structure of compound **5d** can quite logically explain the indicated changes in the aliphatic region of the proton spectrum, but cannot be in any way an explanation for changes in the position in the spectrum of aromatic H18(22) protons, which are much farther from the five-membered ring than, for example, the *ortho*-protons H12(16).

Analysis of the NOESY spectrum of compound **5d** (Fig. S58) gives an unambiguous answer about its actual structure. The variant of the 6a`-diastereomer proposed above turns out to be impossible, since it assumes the proximity of the H6a`α proton to the H1` proton ($r_{6a`\alpha\text{-}1`}^{(calc.)}$ = 2.41 Å) and a significant distance from the H12 and H16 *ortho*-protons ($r_{6a`\alpha\text{-}12}^{(calc.)}$ = 4.24 Å; $r_{6a`\alpha\text{-}16}^{(calc.)}$ = 3.67 Å). However, only very weak (about 0.4%) cross peak 6a`/1` and an intense (about 6.9%) cross peak 6a`/12 are observed in the NOESY spectrum.

The most important source of information about the actual structure of the minor product **5d** is undoubtedly the spatial interactions of the H1 proton, which are indicated by double arrows in Fig. S58 (right fragment). Among them, the interaction with the Ha` proton, which has a β-orientation, plays a decisive role in asserting that the minor product **5d** is an epimer with respect to the dominant product **4d**. According to the calculations by the MM2 method, this distance in the **5d** epimer should be 2.7 Å, which corresponds to the 6a`/1 cross peak with an intensity of 5.7% in the NOESY spectrum. No less important for proving the structure of compound **5d** could be the discovery of a significant (2.4 Å) proximity of the H1 proton at 7.61 ppm. with *ortho*-proton H12 at 7.48 ppm. This interaction is shown in Fig. S58 by a red double arrow), however, due to the spectral closeness of the corresponding signals (Δδ = 0.13 ppm), the cross peak at 400 MHz is covered by diagonal signals and is inaccessible for observation.

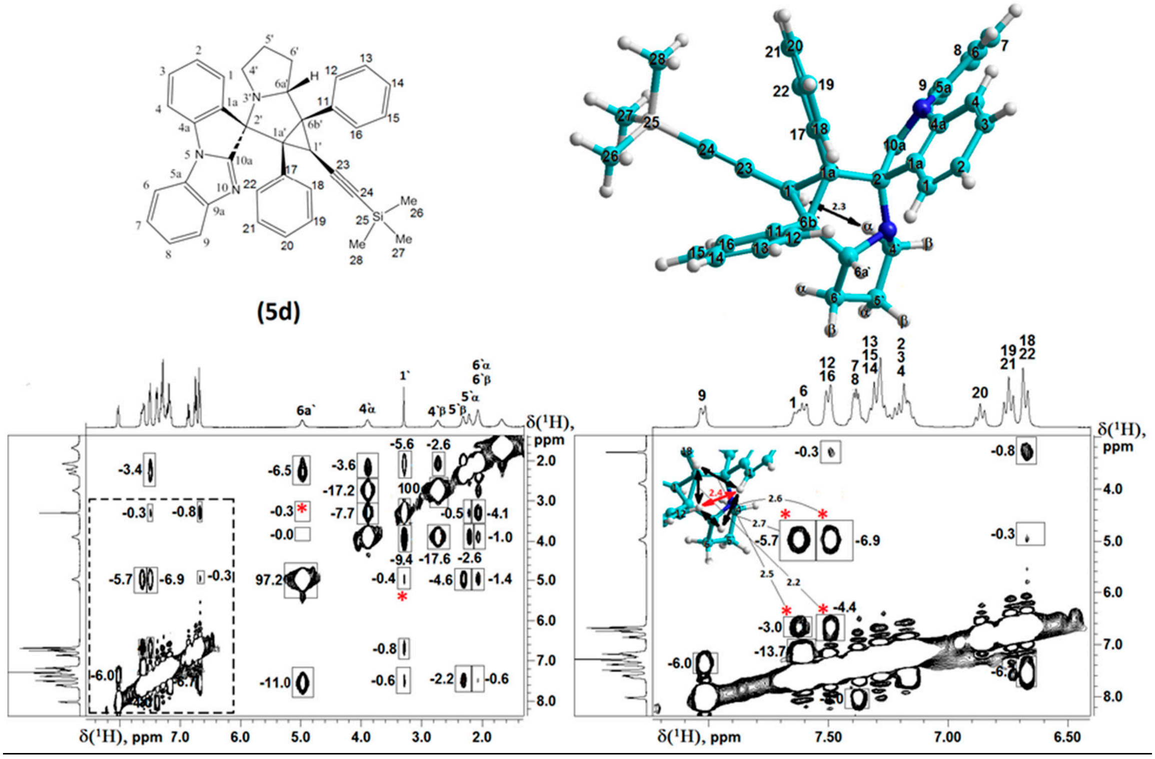

Fig. S58 The interproton distances most important for proving the structure (marked with a red star) and their corresponding cross peaks are shown with double arrows and rectangles on the 3D model of the molecule and on the spectrum, respectively. The calculated (MM2) distance values are given by figures in angstroms (Å), and the volume integrals are given in % relatively to the intensity of the diagonal signal of the proton H1`.

The structure of epimer **5d**, as well as product **4d**, is characterized by the proximity of H1`-H4`α proton pairs in the α-region of this molecule ($r^{(calc.)}$ = 2.3 Å; cross-peak intensity is equal to 7.7%) and also the proximity of *ortho*-protons H12 and H18 of two aromatic rings ($r^{(calc.)}$ = 2.2 Å; cross-peak intensity is equal to 4.4%) and H1-H18 protons ($r^{(calc.)}$ = 2.5 Å; cross-peak intensity is equal to 3.0%) in its β-region. All these cross peaks, which are essential for proving the structure of epimer **5d**, are noted in the NOESY spectrum in Fig. S58 with a red asterisk.

Thus, the epimeric structure of the minor product **5d** was proved by NOE measurements, and the dynamic mobility of the 5-membered aliphatic ring, similar to that observed in the dominant product **4d**, was found based on the broadening of some signals in both the ^{1}H and ^{13}C spectra. A distinctive point in the case of **5d** is the fact that the broadening is observed not only in the aliphatic regions of these spectra, but also in the region of aromatic protons (see the broadening of the H1 doublet signal at 7.61 ppm in Fig. S57) and aromatic carbon-13 atoms on Fig. S59.

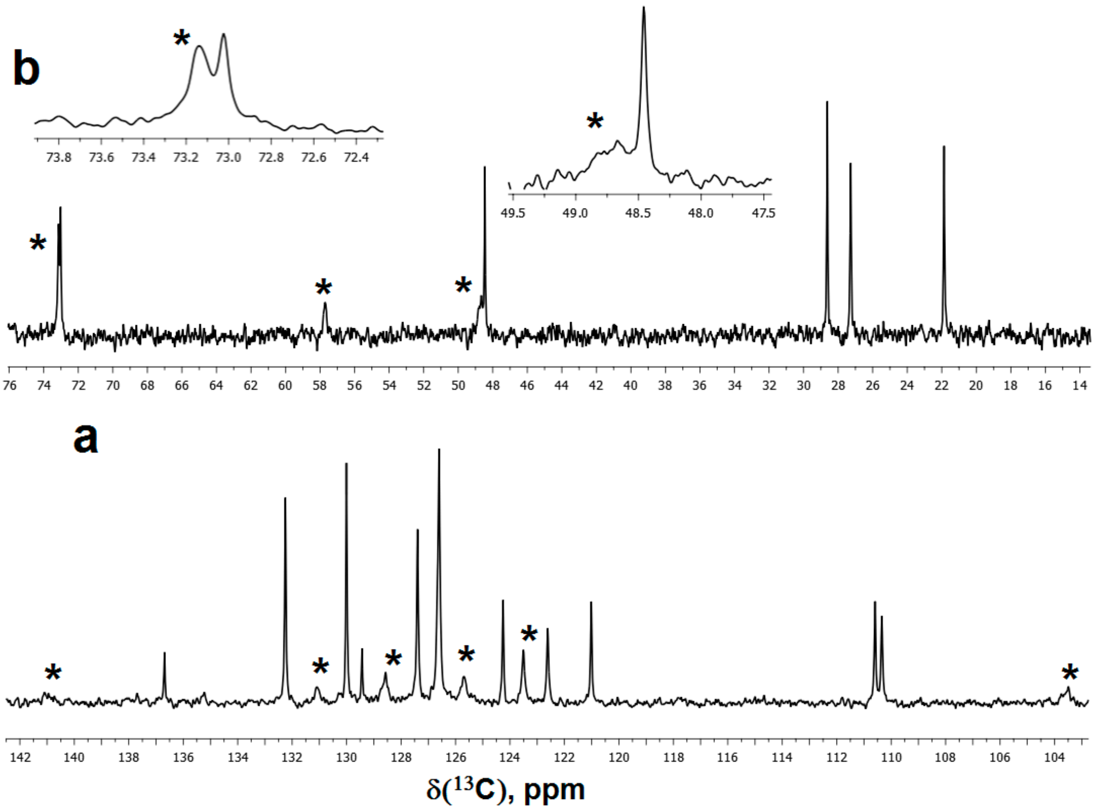

Fig. S59 Aromatic (a) and aliphatic (b) fragments of ^{13}C NMR Spectra of compound **5d**. Dynamic effects are depicted by asterisk.

It should be also noted that for product **5d** in the NOESY spectrum, there is no significant increase in the integrated intensity of the 6a`/4`β cross peak associated with dynamic averaging of the observed (i.e., effective) NOE, as was the case for product **4d** (See Fig. S53). This may indicate a shift in the conformational equilibrium in product **5d** towards the more energetically favorable conformer (**A**) as compared to the dominant product **4d**. A separate article will be devoted to a more detailed study of the dynamic equilibrium processes found in the studied compounds.

3. X-ray crystallographic data for compounds 4f and 8f

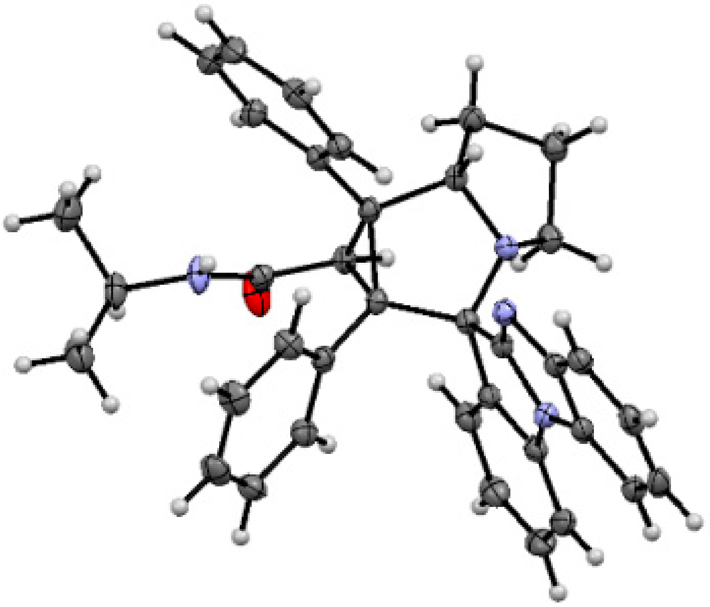

Figure S60. ORTEP representation of the molecular structure of **4f** (CCDC 2195042). Thermal ellipsoids are drawn at 50% probability level.

Table S1. Crystal data for compound **4f**

Empirical formula	$C_{37}H_{34}N_4O$
Formula weight	550.68
Temperature/K	99.9(4)
Crystal system	Monoclinic
Space group	$P2_1/c$
a/Å	18.6509(4)
b/Å	8.5325(2)
c/Å	17.8670(4)
α/°	90
β/°	98.274(2)
γ/°	90
Volume/Å^3	2813.74(11)
Z	4
ρ_{calc}g/cm^3	1.300
Radiation	CuKα (λ = 1.54184)

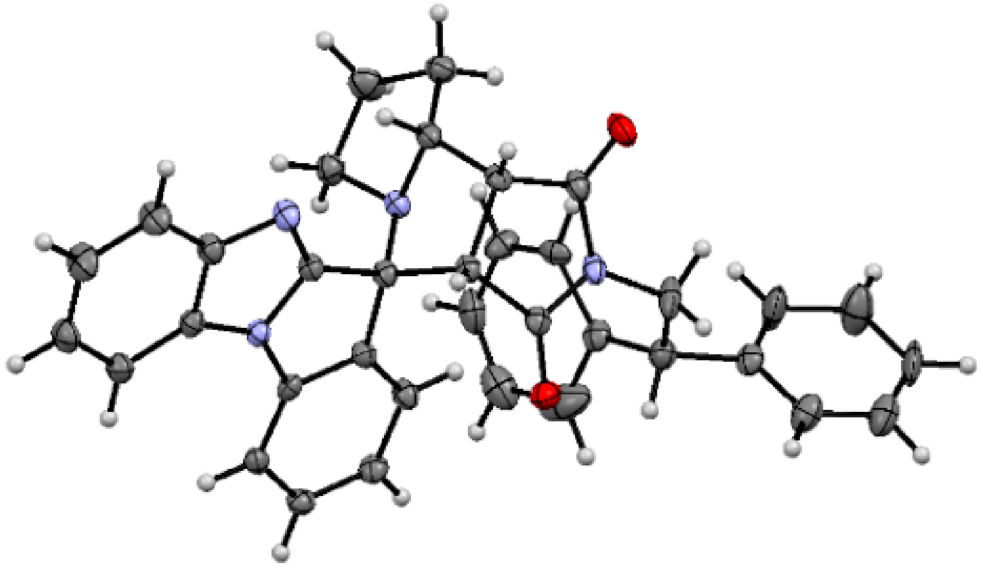

Figure S61. ORTEP representation of the molecular structure of **8f** (CCDC 2195043). Thermal ellipsoids are drawn at 50% probability level.

Table S2. Crystal data for compound **8f**

Empirical formula	$C_{36.35}H_{30.35}N_4O_2$
Formula weight	555.21
Temperature/K	99.8(7)
Crystal system	Monoclinic
Space group	C2/c
a/Å	16.6469(3)
b/Å	22.9614(3)
c/Å	15.4065(3)
α/°	90
β/°	111.421(2)
γ/°	90
Volume/Å^3	5482.12(18)
Z	8
ρ_{calc}g/cm^3	1.345
Radiation	Cu Kα (λ = 1.54184)

4. Bioassay details

MTS assay. A colorimetric MTS assay was used for assessing cell metabolic activity. This method in based on the reduction of 3-(4,5-dimethylthiazol-2-yl)-5-(3-carboxymethoxyphenyl)-2-(4-sulfophenyl)-2H-tetrazolium compound (MTS) to colored and soluble in cell culture media formazan product by NAD(P)H-dependent dehydrogenase enzymes. Since this conversion and such decrease of MTS can only occur in metabolically active cells, the level of activity is the measure of the viability of the cells. Shortly, cells were seeded in a 96-well microtiter plates at a density of $5x10^3$ cells per well in 100 µL of complete medium and allowed to grow and adhere onto the wells during 24h at 37°C. After that the cells were treated with various concentrations of the compounds for a period of 24 or 72 h. After the treatment, 20 µL of MTS reagent was added into each well and incubated at 37°C for 2 h. Finally the absorbance was recorded at 490 nm using 96 well plate reader "Multiskan GO" (Thermo Fisher Scientific, USA).

Table S3. K562 cells viability after the treatment for 24 h

	1	2	5	10	30
**4a**	92.7<br>(3.2)	90.6<br>(1.4)	84.8<br>(2.1)	85.2<br>(4.7)	85.7<br>(5.1)
**4f**	95.7<br>(4.9)	78.8<br>(7.3)	63.9<br>(2.7)	63.8<br>(2.5)	59.4<br>(2.1)
**6b**	88.0<br>(5.9)	87.9<br>(2.2)	84.5<br>(3.6)	87.3<br>(0.6)	84.2<br>(1.3)
**6c**	91.1<br>(4.1)	87.2<br>(4.5)	89.2<br>(5.7)	89.0<br>(2.2)	83.1<br>(2.4)
**6d**	93.9<br>(2.8)	88.0<br>(3.5)	83.8<br>(1.6)	89.3<br>(2.2)	84.1<br>(5.6)
**6e**	88.8<br>(5.4)	86.0<br>(3.0)	89.7<br>(2.5)	89.2<br>(3.9)	85.8<br>(2.9)
**6i**	95.3<br>(2.0)	85.8<br>(0.5)	87.8<br>(5.4)	73.4<br>(10.7)	54.5<br>(1.4)

Table S4. K562 cells viability after the treatment for 72 h

	1	2	5	10	30
**4a**	106.7<br>(2.7)	105.7<br>(3.3)	100.9<br>(8.3)	88.7<br>(1.7)	90.4<br>(5.4)
**4f**	106.5<br>(1.7)	97.8<br>(8.9)	41.5<br>(7.7)	33.1<br>(1.0)	29.3<br>(1.9)
**6b**	109.7<br>(4.5)	114.3<br>(2.6)	84.4<br>(10.6)	73.8<br>(6.7)	73.3<br>(3.0)
**6c**	100.4<br>(1.8)	94.3<br>(4.7)	102.2<br>(3.3)	93.1<br>(11.0)	97.6<br>(1.1)
**6d**	103.6<br>(7.0)	67.9<br>(15.5)	68.8<br>(2.1)	63.0<br>(5.5)	61.8<br>(3.7)
**6e**	108.3<br>(4.6)	97.7<br>(5.6)	80.8<br>(6.6)	74.5<br>(2.6)	60.5<br>(7.7)
**6i**	108.8<br>(7.2)	94.8<br>(8.1)	67.9<br>(13.9)	53.3<br>(4.8)	28.1<br>(7.1)

5. Computational data

Computational methodology: The full geometry optimization of reactants, products and transition states structures (TSs) were performed at DFT/HF level of theory using M062x hybrid exchange-correlation functional [11] and cc-pVDZ basis set [12]. The polarizable continuum model (PCM) was used to calculate solvent effects of 1,4-dioxane [13]. The optimizations were carried out using the Berny analytical gradient optimization method [14]. All stationary points were described by harmonic vibrational frequency calculations to prove the location of correct minima (only real frequencies) and transition states (only one imaginary frequency). For the transition states, the normal modes corresponding to the imaginary frequencies were related to the vibrations of new developing bonds. IRC calculations were performed to check the energy profiles connecting each TS to the two associated minima of the proposed mechanism [15]. Thermal corrections to enthalpy and entropy values were evaluated at 298.15 K and 1.0 atm. All calculations were performed using Gaussian 09 computational program package [16].

Table S5. Energies (a.u.) and cartesian coordinates of stationary points for reactants, intermediates, products and the transition states (M062x/cc-pVDZ, PCM = 1,4-dioxane).

<table>
<tr>
<td>Carbon dioxide
E_0 = -188.514763
E (298 K) = -188.512146
H (298 K) = -188.511201
G (298 K) = -188.535457
Imaginary frequencies = 0
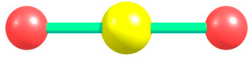
Cartesian coordinates:
O 0.000000 0.000000 1.161591
C 0.000000 0.000000 0.000000
O 0.000000 0.000000 -1.161591</td>
<td>Water
E_0 = -76.374085
E (298 K) = -76.371249
H (298 K) = -76.370305
G (298 K) = -76.392399
Imaginary frequencies = 0
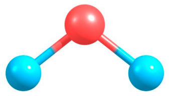
Cartesian coordinates:
H -5.748808 0.818836 -0.664458
H -4.508199 1.415114 -0.021069
O -5.473266 1.448328 0.014768</td>
</tr>
<tr>
<td>1,2-Diphenyl-3-vinylcyclopropene (1c)
E_0 = -655.508865
E (298 K) = -655.494250
H (298 K) = -655.493306
G (298 K) = -655.553836
Imaginary frequencies = 0
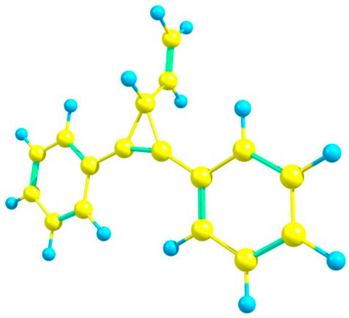
Cartesian coordinates:
C -1.620294 0.388120 -0.542003</td>
<td>11H-Benzo[4,5]imidazo[1,2-a]indol-11-one (2)
E_0 = -722.494471
E (298 K) = -722.483154
H (298 K) = -722.482210
G (298 K) = -722.532138
Imaginary frequencies = 0
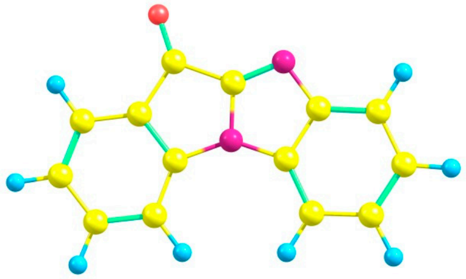
Cartesian coordinates:
H -6.221819 -1.695654 0.000000</td>
</tr>
</table>

C	-2.169939	-0.996551	-0.276953
C	-0.865992	-0.897914	-0.285408
C	0.482019	-1.420426	-0.154551
C	1.568675	-0.542364	-0.270158
C	2.871887	-1.019377	-0.146766
C	3.099809	-2.375417	0.092354
C	2.020744	-3.255308	0.209158
C	0.717704	-2.782497	0.086808
H	-0.132312	-3.469147	0.177420
H	1.372411	0.521621	-0.454695
H	3.717798	-0.327432	-0.237438
H	4.125707	-2.750703	0.189108
H	2.199296	-4.320688	0.397375
C	-3.429121	-1.704711	-0.135135
C	-4.625560	-0.977538	-0.210136
C	-5.850842	-1.627132	-0.081574
C	-5.891770	-3.007478	0.121383
C	-4.702907	-3.737705	0.198120
C	-3.476718	-3.092025	0.071661
H	-2.541765	-3.661978	0.129099
H	-6.856457	-3.519425	0.221323
H	-4.733884	-4.822175	0.357799
H	-4.576574	0.107649	-0.367747
H	-6.782977	-1.052565	-0.140495
C	-1.697153	1.440821	0.510500
H	-1.658556	0.781780	-1.575591
C	-1.814685	2.747456	0.262285
H	-1.653638	1.078309	1.550585
H	-1.860502	3.125058	-0.769221
H	-1.869965	3.484083	1.072895

C	-5.127072	-1.738663	0.000000
H	-4.856841	0.432351	0.000000
H	-5.084222	-3.894450	0.000000
C	-4.379188	-0.554968	0.000000
C	-4.483566	-2.977102	0.000000
C	-2.998389	-0.653874	0.000000
C	-3.084651	-3.088177	0.000000
O	-2.111174	1.618551	0.000000
C	-2.362602	-1.907229	0.000000
H	-2.596790	-4.067919	0.000000
C	-1.955949	0.424066	0.000000
N	-0.966020	-1.692890	0.000000
C	-0.657450	-0.349715	0.000000
H	-0.188915	-4.525345	0.000000
C	0.229980	-2.376518	0.000000
N	0.616484	-0.091490	0.000000
C	0.568593	-3.735000	0.000000
C	1.211481	-1.350454	0.000000
C	1.923165	-4.036164	0.000000
C	2.573372	-1.684520	0.000000
H	2.234412	-5.087517	0.000000
C	2.912480	-3.028346	0.000000
H	3.330801	-0.892734	0.000000
H	3.968991	-3.320498	0.000000

L-proline (**3a**)

E_0 = -400.835993

E (298 K) = -400.828364

H (298 K) = -400.827420

G (298 K) = -400.869197

Imaginary frequencies = 0

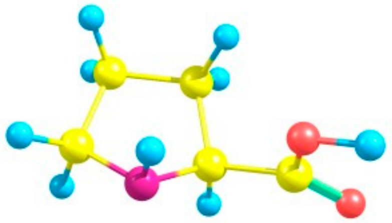

Cartesian coordinates:

8	-0.816772	-1.333335	1.016523
8	0.004182	-0.742866	-0.983543
7	-3.331238	-1.457367	-0.062721
6	-0.939558	-0.863512	-0.242622
6	-2.366448	-0.474970	-0.553873
6	-2.750223	0.876549	0.111551
6	-4.597290	-0.718236	-0.054807
6	-4.271526	0.741463	0.357457

OX-1

E_0 = -1046.965809

E (298 K) = -1046.948814

H (298 K) = -1046.947870

G (298 K) = -1047.010827

Imaginary frequencies = 0

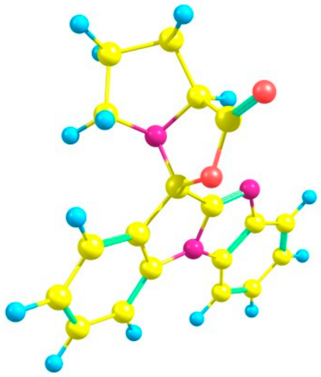

Cartesian coordinates:

O	-3.569835	4.782573	2.554058
O	-3.397663	2.650654	1.852117
N	-3.750922	2.947770	-0.423241
N	-0.680946	2.537716	0.196929

1	-3.071126	-1.678052	0.903029
1	0.123049	-1.542973	1.156239
1	-2.444505	-0.397395	-1.653458
1	-5.327821	-1.211640	0.607863
1	-5.016505	-0.735886	-1.079286
1	-2.481130	1.740631	-0.518055
1	-2.206313	0.972964	1.068802
1	-4.853723	1.469258	-0.233243
1	-4.511960	0.917029	1.420239

N	-1.521828	0.459883	0.027457
C	-4.453360	5.261691	-0.345474
C	-5.461335	4.378462	-1.110391
C	-5.222250	2.987157	-0.527191
C	-3.457025	3.998707	1.655420
C	-3.394666	4.261197	0.143947
C	0.640096	-0.776749	-0.551346
C	1.988673	-0.511999	-0.754721
C	2.511572	0.793365	-0.652754
C	1.699902	1.875953	-0.341400
C	-0.172631	0.317287	-0.238625
C	0.336971	1.636705	-0.130221
C	-1.736802	1.790160	0.277154
C	-3.215158	2.027471	0.558067
C	-2.904229	-1.652138	0.088530
C	-2.706132	-0.284957	0.189868
C	-3.742992	0.599602	0.534356
C	-4.999789	0.103064	0.837085
C	-4.188377	-2.136154	0.367653
C	-5.219359	-1.277978	0.745882
H	-2.352176	4.529342	-0.104573
H	-4.011451	6.041142	-0.985419
H	-4.911025	5.762624	0.525581
H	-5.217695	4.367330	-2.187047
H	-6.505357	4.711087	-0.993278
H	-5.591827	2.161404	-1.157975
H	-5.702974	2.917623	0.476205
H	0.243164	-1.793802	-0.632786
H	2.662988	-1.340696	-1.000664
H	3.582725	0.952981	-0.822563
H	2.097557	2.893471	-0.257671
H	-5.805772	0.773415	1.155681
H	-6.209130	-1.685539	0.979751
H	-4.377991	-3.213763	0.298683
H	-2.090205	-2.330313	-0.185315

OX-2

E_0 = -1046.963324
E (298 K) = -1046.946277
H (298 K) = -1046.945333
G (298 K) = -1047.008459
Imaginary frequencies = 0

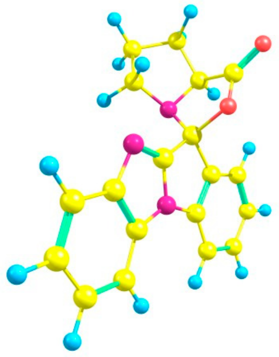

Cartesian coordinates:

ZI-1

E_0 = -1046.939237
E (298 K) = -1046.921329
H (298 K) = -1046.920385
G (298 K) = -1046.985753
Imaginary frequencies = 0

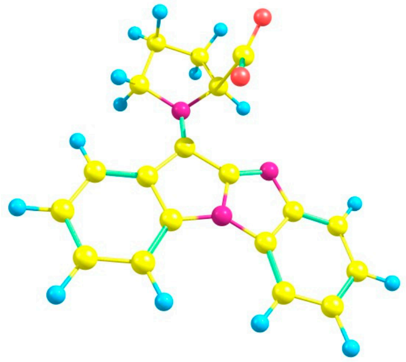

Cartesian coordinates:

O	-3.966115	4.543349	2.695987	C	0.310337	4.214429	0.126175
O	-3.925989	2.505153	1.747382	C	-1.064532	4.151305	0.350452
N	-5.705845	0.409154	0.120210	C	-1.750623	2.931655	0.329308
N	-3.931834	3.089479	-0.505251	C	-1.009726	1.789890	0.082373
N	-3.588058	-0.300910	-0.121857	C	0.390349	1.825032	-0.130062
C	-1.012085	2.099072	0.544781	C	1.050019	3.052607	-0.115943
C	0.172805	1.366973	0.384399	N	-1.444145	0.461227	-0.022995
C	0.132821	0.023327	0.018015	C	-0.396582	-0.382481	-0.310554
C	-1.084932	-0.637531	-0.186203	C	0.833067	0.431987	-0.341124
C	-6.768801	-1.830711	-0.359807	C	-2.592533	-0.307494	-0.046535
C	-6.486168	-3.150700	-0.678244	C	-2.124980	-1.608715	-0.364611
C	-4.072868	-2.750122	-0.672056	N	-0.742366	-1.617451	-0.527053
C	-5.157686	-3.601550	-0.831494	C	-3.946210	-0.031588	0.169863
C	-4.363416	-1.419037	-0.350537	C	-4.823282	-1.102411	0.055392
C	-5.692159	-0.949874	-0.193676	C	-4.374948	-2.402556	-0.259273
C	-4.449399	0.733094	0.148774	C	-3.030970	-2.671875	-0.470698
C	-4.563802	5.412087	-0.192185	N	2.014290	-0.040119	-0.566188
C	-5.434993	4.719422	-1.255278	C	2.300996	-1.443628	-0.464470
C	-5.363175	3.249280	-0.860009	C	2.205063	-1.787220	1.167972
C	-3.844405	3.860183	1.718879	O	1.395160	-1.078059	1.773415
C	-3.605414	4.297518	0.268820	C	3.261965	0.752980	-0.564341
C	-2.222713	1.465187	0.323404	C	4.373534	-0.304136	-0.473463
C	-2.242218	0.105324	-0.019796	C	3.716088	-1.590841	-0.988718
C	-3.657269	2.001611	0.415607	O	2.959745	-2.706896	1.484734
H	-1.115982	-1.697373	-0.457590	H	0.820541	5.183417	0.138934
H	1.138088	1.856138	0.555465	H	-1.624241	5.074057	0.542570
H	1.069020	-0.533913	-0.103250	H	-2.830350	2.886269	0.499641
H	-0.968835	3.149999	0.854198	H	2.124733	3.130395	-0.301574
H	-3.046375	-3.110496	-0.793743	H	-4.305996	0.971043	0.421513
H	-7.308733	-3.862279	-0.814743	H	-5.893845	-0.931719	0.218321
H	-4.976161	-4.653024	-1.083331	H	-5.107080	-3.214874	-0.333116
H	-7.796124	-1.469892	-0.237889	H	-2.669408	-3.677999	-0.709410
H	-2.542537	4.580608	0.161489	H	1.514002	-2.052640	-0.934413
H	-4.016920	6.280301	-0.591878	H	3.246123	1.437009	0.300467
H	-5.159037	5.760623	0.670005	H	3.298210	1.341233	-1.499676
H	-4.991038	4.852408	-2.257589	H	5.267514	0.001053	-1.039288
H	-6.469281	5.098786	-1.279905	H	4.658488	-0.448080	0.583469
H	-5.640168	2.551396	-1.666362	H	3.738060	-1.650556	-2.092589
H	-6.019589	3.040590	0.013250	H	4.167370	-2.496203	-0.556862

ZI-2	AY-1
E_0 = -1046.942785	E_0 = -858.442531
E (298 K) = -1046.924977	E (298 K) = -858.427327
H (298 K) = -1046.924032	H (298 K) = -858.426383
G (298 K) = -1046.989107	G (298 K) = -858.485122
Imaginary frequencies = 0	Imaginary frequencies = 0

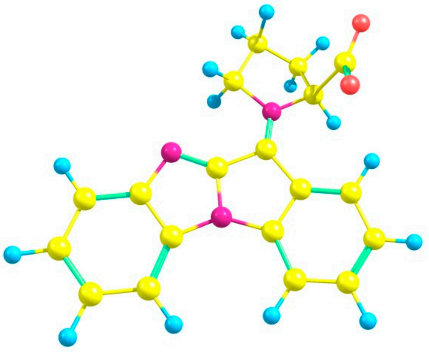

Cartesian coordinates:

C	-4.188263	-0.206888	-0.749013
C	-4.073087	-1.594391	-0.819311
C	-2.834790	-2.231499	-0.668026
C	-1.739317	-1.426573	-0.417960
C	-1.838300	-0.019968	-0.304490
C	-3.069149	0.596718	-0.511021
N	-0.388593	-1.794127	-0.252934
C	0.393621	-0.693060	-0.014662
C	-0.486396	0.492714	0.005896
C	0.447122	-2.890735	-0.218603
C	1.727363	-2.339243	0.050913
N	1.656851	-0.955794	0.178586
C	0.246883	-4.266053	-0.386598
C	1.370111	-5.072679	-0.275124
C	2.650924	-4.540194	-0.007520
C	2.845913	-3.178607	0.156988
N	-0.084598	1.649694	0.411260
C	-0.883462	2.852854	0.510132
C	-1.040470	3.456374	-1.010061
O	-1.341692	2.577806	-1.829946
C	1.334172	1.930563	0.757327
C	1.357491	3.444666	0.971742
C	-0.072884	3.765396	1.415683
O	-0.855106	4.672090	-1.082752
H	-5.162734	0.266351	-0.910037
H	-4.963127	-2.202650	-1.019086
H	-2.741162	-3.318401	-0.752856
H	-3.144716	1.685604	-0.552485
H	-0.739346	-4.691500	-0.597088
H	1.259917	-6.156281	-0.400721
H	3.505141	-5.222554	0.068969
H	3.834211	-2.752826	0.362541
H	-1.897098	2.601954	0.865683
H	1.569439	1.355909	1.672374
H	1.982794	1.553494	-0.048996
H	1.553915	3.959715	0.015378
H	2.130130	3.736427	1.700254
H	-0.230609	3.525847	2.483664
H	-0.351824	4.809638	1.212675

AY-2

E_0 = -858.440746

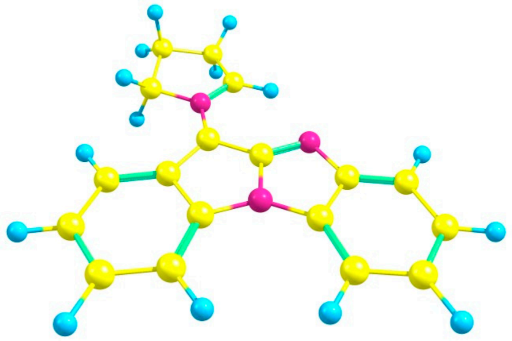

Cartesian coordinates:

H	-3.212360	8.750407	-1.221510
H	-4.360258	5.683683	-1.021462
H	-5.292936	7.830988	-0.364398
H	-0.971995	7.448720	-0.181208
C	-3.141809	8.339739	-0.194403
C	-2.059343	7.309527	-0.134810
N	0.599332	5.837903	-0.056359
H	3.346258	6.524251	-0.042556
C	-0.379413	4.945366	-0.045993
C	1.750324	5.060293	-0.026453
C	3.093029	5.457926	-0.021801
N	-2.535450	6.095507	-0.070069
C	-1.802223	4.933897	-0.053145
N	0.055612	3.631190	-0.012298
C	1.442038	3.670446	0.001031
C	4.078632	4.475224	0.008727
C	-4.016267	6.074590	-0.047124
H	5.134328	4.773014	0.012811
C	-2.221745	3.545902	-0.039310
H	-4.401032	3.402766	-0.116824
C	-1.029605	2.756472	-0.008793
C	2.425137	2.682813	0.031222
C	-3.449115	2.864342	-0.068625
C	3.752544	3.107709	0.034886
H	2.172806	1.617087	0.051870
C	-1.044173	1.367899	0.011506
H	4.555192	2.361273	0.058860
H	-0.109431	0.797503	0.037494
C	-3.465285	1.469128	-0.048092
C	-4.397133	7.538910	0.204473
C	-2.282290	0.723555	-0.004448
H	-4.430401	0.948953	-0.070058
H	-2.323887	-0.371394	0.011711
H	-2.940322	9.191400	0.477778
H	-4.344484	5.389120	0.749690
H	-4.611487	7.686385	1.276103

Cycloadduct **4c**

E_0 = -1514.043463

E (298 K) = -858.425446
H (298 K) = -858.424502
G (298 K) = -858.483720
Imaginary frequencies = 0

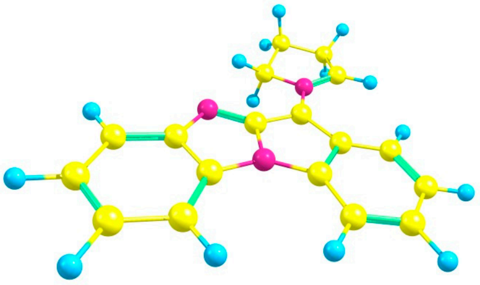

Cartesian coordinates:

H	-2.899439	8.691956	-1.325410
H	-5.121367	7.882228	-0.884599
H	-1.020114	7.484885	-0.366908
C	-3.055308	8.418745	-0.267591
H	-4.508759	5.333588	-0.447411
C	-3.892036	6.211322	-0.250652
C	-1.026150	2.803501	-0.033155
C	-2.220185	3.585773	-0.042085
C	-1.817220	4.978158	-0.052039
N	0.051867	3.685977	-0.037366
C	-1.032870	1.416818	-0.010596
N	-2.602647	6.099288	-0.084113
H	-0.096700	0.848663	-0.005448
H	2.159657	1.656348	-0.033854
C	-4.366415	7.626039	-0.121752
C	-0.381751	5.003731	-0.043939
C	1.437956	3.714714	-0.043042
C	2.415873	2.721264	-0.041187
C	-2.270867	0.767978	0.009713
C	-3.445174	2.904204	-0.010395
C	1.752152	5.101695	-0.052860
C	3.745253	3.139835	-0.049316
N	0.606011	5.883546	-0.052542
H	4.544417	2.389253	-0.048342
C	-3.456279	1.506720	0.015855
H	-2.307201	-0.327250	0.025725
C	3.096703	5.493840	-0.060531
C	4.077638	4.506234	-0.058851
H	3.354522	6.559236	-0.068379
H	5.134787	4.798881	-0.065229
H	-4.401999	3.433984	0.010363
H	-4.419265	0.983032	0.041859
C	-1.976642	7.430239	0.173506
H	-3.036332	9.341234	0.332589
H	-4.838831	7.781311	0.869488
H	-1.755577	7.469378	1.255733

E (298 K) = -1514.014146
H (298 K) = -1514.013201
G (298 K) = -1514.104326
Imaginary frequencies = 0

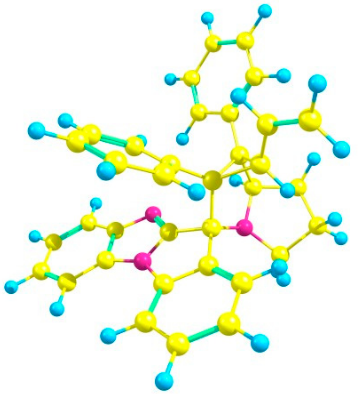

Cartesian coordinates:

N	-0.637593	1.159646	-1.992176
N	1.121890	-1.417510	-1.483350
N	2.419301	0.170665	-0.560302
C	-1.861537	0.373356	-1.917196
C	-2.942722	1.234787	-2.607063
C	-2.341259	2.651296	-2.697042
C	-0.820365	2.409479	-2.714548
C	4.586818	-1.174800	-0.374597
C	5.075229	-2.443980	-0.662937
C	4.259679	-3.434209	-1.245174
C	2.928927	-3.184694	-1.557303
C	3.246710	-0.930131	-0.687279
C	2.410723	-1.915689	-1.275174
C	1.189474	-0.196471	-1.042759
C	0.186562	0.928017	-0.834445
C	3.416603	2.239479	0.484258
C	2.401754	1.502598	-0.106355
C	1.110618	2.024950	-0.312169
C	0.828110	3.318754	0.100744
C	3.117090	3.549165	0.876520
C	1.843730	4.085048	0.688775
H	-1.752019	-0.602955	-2.431782
H	-3.891996	1.206355	-2.042761
H	-3.146955	0.840838	-3.617951
H	-2.625455	3.257157	-1.817566
H	-2.685832	3.198015	-3.591128
H	-0.242106	3.231175	-2.257567
H	-0.462312	2.311339	-3.761206
H	6.120501	-2.679179	-0.430244
H	4.687537	-4.421713	-1.454953
H	2.289171	-3.949666	-2.011985
H	5.227298	-0.410353	0.077537
H	-0.179384	3.737363	-0.018946
H	1.631927	5.111153	1.010052
H	4.410979	1.812408	0.648597
H	3.899210	4.158662	1.344401

C	-2.025579	1.321634	0.440404
C	-2.072815	0.068393	-0.418564
C	-0.791773	0.448182	0.283604
C	-0.150615	-0.177643	1.488401
C	0.280275	0.664543	2.524281
C	0.977426	0.151769	3.616041
C	1.253629	-1.214598	3.687549
C	0.828148	-2.059927	2.663429
C	0.131503	-1.545687	1.569355
H	-0.181608	-2.211278	0.759211
H	0.060019	1.739067	2.462124
H	1.309033	0.824799	4.416090
H	1.804094	-1.621496	4.544816
H	1.044817	-3.133950	2.710625
C	-2.906773	-1.138030	-0.122834
C	-4.175959	-1.048326	0.456200
C	-4.948057	-2.196254	0.648341
C	-4.464315	-3.441976	0.252653
C	-3.206506	-3.536740	-0.348002
C	-2.436173	-2.392767	-0.538540
H	-1.448473	-2.455656	-1.020706
H	-5.071356	-4.342791	0.405351
H	-2.823851	-4.512114	-0.672508
H	-4.564141	-0.066612	0.756144
H	-5.940603	-2.112551	1.107804
C	-2.685293	1.394169	1.767401
H	-2.026668	2.265885	-0.119707
C	-3.258111	2.501451	2.245468
H	-2.673903	0.475243	2.372247
H	-3.277081	3.431298	1.659621
H	-3.726982	2.519086	3.236513

Cycloadduct **4c'**

E_0 = -1514.032302
E (298 K) = -1514.003333
H (298 K) = -1514.002389
G (298 K) = -1514.091873
Imaginary frequencies = 0

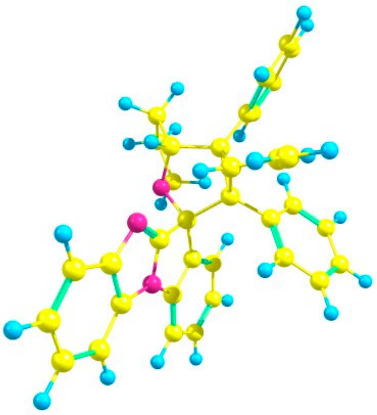

Cartesian coordinates:

N	-0.042469	-0.705924	-1.842661
N	2.009157	-1.975685	0.150642

Cycloadduct **5c**

E_0 = -1514.044549
E (298 K) = -1514.016288
H (298 K) = -1514.015343
G (298 K) = -1514.102205
Imaginary frequencies = 0

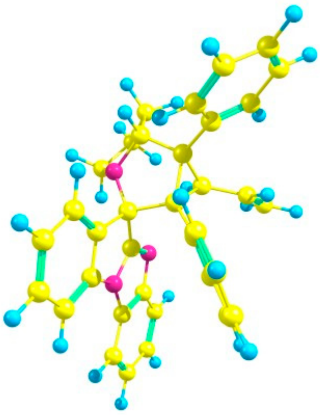

Cartesian coordinates:

N	0.489996	-1.117806	-2.009537
N	-1.991838	-1.884108	0.031468

N	2.840692	0.060686	-0.279999	N	-2.503557	0.216428	-0.588947
C	-0.968396	-1.720342	-1.305334	C	1.907901	-1.223394	-1.665001
C	-1.851057	-2.096542	-2.502053	C	2.291034	-2.703525	-1.906774
C	-1.738879	-0.892854	-3.470270	C	0.951915	-3.450598	-2.040569
C	-0.874201	0.125536	-2.718344	C	-0.016365	-2.373888	-2.557185
C	5.310540	-0.440165	0.153941	C	-4.890975	0.304954	0.317861
C	6.125025	-1.500030	0.534150	C	-5.801144	-0.504540	0.986335
C	5.595820	-2.776302	0.813052	C	-5.480254	-1.825205	1.363460
C	4.234106	-3.031774	0.716149	C	-4.236807	-2.375612	1.083364
C	3.940432	-0.703712	0.057955	C	-3.639473	-0.254049	0.038186
C	3.392181	-1.981713	0.330679	C	-3.298793	-1.579301	0.414406
C	1.736857	-0.752136	-0.206228	C	-1.574388	-0.795432	-0.550828
C	0.490032	0.002417	-0.667765	C	-0.242056	-0.290550	-1.079183
C	3.318008	2.452010	-0.900243	C	-2.606282	2.567865	-1.537477
C	2.497725	1.346028	-0.731572	C	-1.993928	1.359318	-1.243610
C	1.114789	1.372033	-0.975593	C	-0.673957	1.076246	-1.618409
C	0.534207	2.569539	-1.375117	C	0.054198	2.002932	-2.340770
C	2.714420	3.639324	-1.324555	C	-1.849099	3.508576	-2.248180
C	1.340562	3.699810	-1.553945	C	-0.543358	3.231444	-2.654787
H	-0.380347	-2.581117	-0.939978	H	2.534437	-0.562212	-2.300027
H	-2.888614	-2.305044	-2.193816	H	2.918372	-3.087375	-1.082038
H	-1.458030	-3.011990	-2.973608	H	2.876456	-2.793335	-2.838665
H	-2.721638	-0.469298	-3.741279	H	0.604842	-3.825466	-1.061055
H	-1.239292	-1.195849	-4.405858	H	1.018598	-4.317450	-2.719723
H	-1.518393	0.813441	-2.121277	H	-1.057135	-2.553394	-2.240090
H	-0.246872	0.746620	-3.379992	H	-0.001702	-2.342157	-3.666431
H	7.205979	-1.337739	0.619539	H	-6.793748	-0.105641	1.226782
H	6.276667	-3.582418	1.110715	H	-6.231377	-2.426139	1.889561
H	3.814714	-4.021873	0.927820	H	-3.977965	-3.400077	1.373992
H	5.730703	0.547979	-0.059946	H	-5.147890	1.329064	0.027750
H	-0.546926	2.643924	-1.533153	H	1.079278	1.769257	-2.659772
H	0.882012	4.643594	-1.869901	H	0.017392	3.981903	-3.224048
H	4.392976	2.401440	-0.700877	H	-3.636128	2.782505	-1.234383
H	3.331458	4.534519	-1.466719	H	-2.300055	4.476432	-2.497234
C	-0.847203	-1.257546	1.197038	C	1.112970	-1.450205	0.741512
C	-0.686435	0.022572	0.378792	C	2.035787	-0.722191	-0.218240
C	-1.667674	-1.052154	-0.065640	C	0.702354	-0.109830	0.152692
C	-3.171851	-1.021251	-0.016592	C	0.395594	1.039987	1.064878
C	-3.853902	-1.900436	0.833523	C	-0.629993	0.872112	2.007346
C	-5.248631	-1.961070	0.840264	C	-1.036294	1.934725	2.812014
C	-5.990918	-1.149254	-0.015231	C	-0.413283	3.178316	2.692595
C	-5.325890	-0.286121	-0.886960	C	0.612614	3.351662	1.763735
C	-3.933777	-0.231684	-0.891126	C	1.009621	2.290405	0.949968
H	-3.429551	0.437410	-1.599418	H	1.802497	2.439035	0.209271
H	-3.276374	-2.550278	1.502342	H	-1.108131	-0.113473	2.104691
H	-5.757381	-2.655366	1.520322	H	-1.842716	1.788523	3.540824
H	-7.086703	-1.194395	-0.010217	H	-0.728331	4.015714	3.327168
H	-5.896276	0.348099	-1.576563	H	1.105853	4.326161	1.664317
C	-0.976890	1.328539	1.058101	C	3.393868	-0.223462	0.172537
C	-0.032659	1.800223	1.981654	C	4.212751	-0.957312	1.037502
C	-0.193258	3.038013	2.596627	C	5.505441	-0.522176	1.334380
C	-1.314937	3.820345	2.309447	C	6.001816	0.646753	0.759480

C	-2.266464	3.352582	1.406334
C	-2.096420	2.114403	0.780819
H	-2.845987	1.761447	0.065368
H	-1.446406	4.794882	2.794979
H	-3.153447	3.956680	1.179256
H	0.846397	1.178785	2.208161
H	0.559954	3.395273	3.309321
C	-1.438093	-1.133156	2.554747
H	-0.035898	-1.992342	1.121792
C	-1.010543	-1.840677	3.603111
H	-2.253245	-0.404445	2.680080
H	-0.193495	-2.568437	3.501634
H	-1.457403	-1.713788	4.596516

C	5.203713	1.372226	-0.127487
C	3.914418	0.934765	-0.421446
H	3.296024	1.496710	-1.135536
H	7.016366	0.990259	0.994985
H	5.591064	2.284946	-0.596355
H	3.828219	-1.883724	1.482507
H	6.131514	-1.106873	2.019401
C	1.394603	-1.527979	2.196673
H	0.574152	-2.316497	0.337600
C	1.028689	-2.562404	2.957352
H	1.915174	-0.669295	2.647044
H	0.495849	-3.421934	2.527275
H	1.239865	-2.580880	4.033315

Cycloadduct **5c'**

E_0 = -1514.050099

E (298 K) = -1514.021010

H (298 K) = -1514.020066

G (298 K) = -1514.109524

Imaginary frequencies = 0

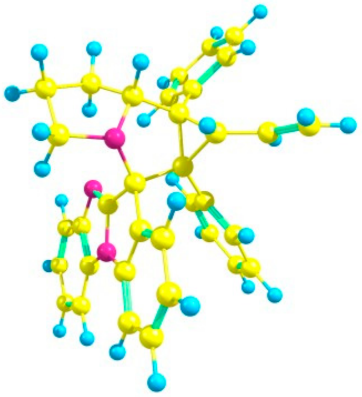

Cartesian coordinates:

N	0.540272	0.977168	-2.247719
N	-0.853264	-1.752575	-0.919280
N	-2.330161	-0.142537	-0.400639
C	1.867445	0.355427	-2.045508
C	1.785984	-0.987600	-2.775891
C	0.997674	-0.579893	-4.028395
C	-0.032712	0.446118	-3.499063
C	-4.248727	-1.632454	0.392236
C	-4.549612	-2.979975	0.548414
C	-3.623629	-3.988302	0.212365
C	-2.366241	-3.677757	-0.289076
C	-2.980871	-1.325204	-0.111451
C	-2.036798	-2.326684	-0.452424
C	-1.080265	-0.470409	-0.865632
C	-0.250700	0.804608	-1.042823
C	-3.632627	1.997259	-0.013726
C	-2.520040	1.255150	-0.378179
C	-1.334480	1.854589	-0.827672
C	-1.258700	3.229617	-0.958648
C	-3.535507	3.390255	-0.128281
C	-2.373628	4.001031	-0.601625

TS-ZI-1

E_0 = -1046.935645

E (298 K) = -1046.918539

H (298 K) = -1046.917594

G (298 K) = -1046.980856

Imaginary frequencies = 1 (-221 cm^{-1})

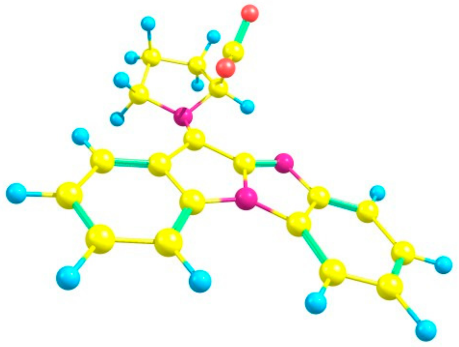

Cartesian coordinates:

H	-2.041510	3.986163	-1.216076
H	-3.536900	1.008144	-1.523972
H	-4.280875	3.146209	-0.698170
H	-0.086556	2.685853	-0.383098
C	-2.149645	3.518797	-0.221749
C	-1.095227	2.453261	-0.007419
N	1.529187	0.984542	-0.498034
H	4.286037	1.614414	-0.626373
C	0.551149	0.138075	-0.386743
C	2.669197	0.181917	-0.454554
C	4.015804	0.557468	-0.526112
N	-1.637729	1.251893	-0.619671
C	-0.936608	0.175573	-0.351463
N	0.939212	-1.174006	-0.275865
C	2.321214	-1.185745	-0.317159
C	4.973937	-0.444432	-0.462573
C	-3.114402	1.284111	-0.541716
H	6.036453	-0.179945	-0.513571
C	-1.345867	-1.250473	-0.360179
H	-3.513301	-1.325567	-0.535366
C	-0.170378	-2.031719	-0.265080
C	3.282125	-2.198924	-0.253683

H	2.637287	0.984552	-2.542359	C	-2.581640	-1.887084	-0.429068
H	1.196134	-1.706804	-2.182107	C	4.610834	-1.800412	-0.329806
H	2.776603	-1.419657	-2.998828	H	3.012070	-3.253927	-0.143756
H	0.513277	-1.432122	-4.533449	C	-0.200806	-3.413613	-0.204308
H	1.671579	-0.094351	-4.759026	H	5.398172	-2.561843	-0.281567
H	-1.005421	-0.042305	-3.294337	H	0.718088	-4.002068	-0.123006
H	-0.217248	1.257138	-4.226864	C	-2.626853	-3.284117	-0.374625
H	-5.531977	-3.265254	0.942916	C	-3.459180	2.722628	-0.099286
H	-3.905341	-5.038631	0.352046	C	-1.456034	-4.030814	-0.254867
H	-1.641197	-4.456264	-0.552714	H	-3.594992	-3.793471	-0.426752
H	-4.972743	-0.854841	0.656872	H	-1.513738	-5.124653	-0.207787
H	-0.339504	3.692244	-1.338941	H	-2.049989	4.288938	0.558337
H	-2.334367	5.092444	-0.695664	H	-3.444190	0.554609	0.217818
H	-4.551118	1.522140	0.345311	H	-3.779403	2.709034	0.956592
H	-4.396986	4.008113	0.151294	O	-0.979352	2.940661	2.354208
C	1.908707	1.815332	-0.023603	C	-0.948478	2.023939	1.536685
C	0.788398	0.791453	0.103478	O	-0.828456	0.774260	1.704250
C	2.103401	0.392087	-0.534716				
C	3.122472	-0.554131	0.023138				
C	4.410183	-0.112936	0.346495				
C	5.376603	-1.013104	0.797738				
C	5.067758	-2.367027	0.923688				
C	3.790501	-2.819134	0.586678				
C	2.826465	-1.919769	0.135725				
H	1.824868	-2.273590	-0.145686				
H	4.652848	0.952480	0.243812				
H	6.380582	-0.651790	1.051932				
H	5.826061	-3.074186	1.281389				
H	3.542952	-3.884119	0.674498				
C	0.241745	0.467495	1.466402				
C	-0.301141	1.521885	2.215924				
C	-0.926775	1.280966	3.437017				
C	-1.014464	-0.021650	3.930752				
C	-0.473206	-1.075653	3.196258				
C	0.149393	-0.833820	1.970736				
H	0.549205	-1.670776	1.391649				
H	-1.507060	-0.215202	4.891484				
H	-0.540425	-2.102673	3.574802				
H	-0.228449	2.545016	1.822682				
H	-1.349419	2.117463	4.006882				
C	2.577550	2.279622	1.217054				
H	1.801951	2.575510	-0.809401				
C	3.026773	3.524925	1.389803				
H	2.690947	1.538012	2.022058				
H	2.921049	4.282628	0.600874				
H	3.512769	3.830836	2.323921				

**TS-ZI-2**	**TS-AY-1**
E_0 = -1046.935602	E_0 = -1046.938396
E (298 K) = -1046.918461	E (298 K) = -1046.920548
H (298 K) = -1046.917516	H (298 K) = -1046.919604
G (298 K) = -1046.981150	G (298 K) = -1046.984962
Imaginary frequencies = 1 (-205 cm^{-1})	Imaginary frequencies = 1 (-381 cm^{-1})

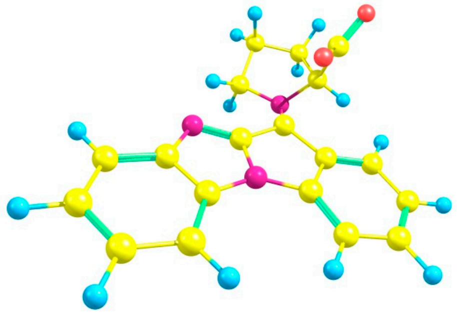

Cartesian coordinates:

H	-2.762743	3.979997	-0.911237
H	-3.417473	1.354411	-1.855804
H	-4.585729	2.516400	-0.056526
H	-0.442302	3.207079	-0.675718
C	-2.540164	3.375879	-0.012880
C	-1.225346	2.623498	-0.167937
N	-2.086876	-1.770191	-0.776970
H	-3.376714	-4.291507	-0.751647
C	-1.023514	-1.020006	-0.861380
C	-1.594792	-3.062851	-0.907706
C	-2.289887	-4.281413	-0.889188
N	-1.569608	1.392041	-0.859589
C	-0.690999	0.423647	-0.771345
N	0.143181	-1.714978	-1.048000
C	-0.186406	-3.051550	-1.082107
C	-1.557050	-5.446415	-1.044651
C	-3.037334	1.171523	-0.833274
H	-2.070997	-6.414396	-1.032222
C	0.785021	0.478256	-1.012228
H	1.394701	2.564054	-0.957546
C	1.245254	-0.844727	-1.166924
C	0.557515	-4.227967	-1.238723
C	1.696792	1.522881	-1.095888
C	-0.154454	-5.417431	-1.216770
H	1.644228	-4.219003	-1.367455
C	2.571049	-1.154283	-1.408985
H	0.386813	-6.363498	-1.333909
H	2.905270	-2.189387	-1.527296
C	3.042473	1.229750	-1.344232
C	-3.542988	2.228628	0.151737
C	3.472212	-0.086767	-1.496543
H	3.765676	2.049925	-1.408245
H	4.531664	-0.295347	-1.684380
H	-2.473532	4.036971	0.864897
H	-3.261535	0.133185	-0.549473
H	-3.488371	1.833236	1.182464
O	-0.566139	3.025126	2.103840
C	-0.654876	2.136804	1.256833
O	-0.376775	0.908907	1.314759

TS-AY-2

E_0 = -1046.939316

E (298 K) = -1046.921368

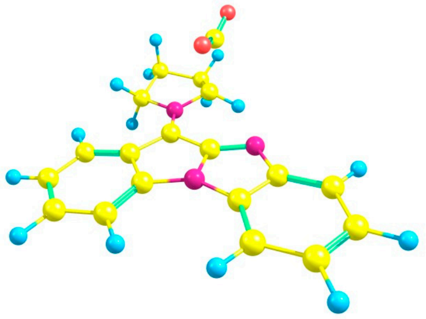

Cartesian coordinates:

C	0.243667	4.219337	0.173601
C	-1.136051	4.146118	0.366033
C	-1.805710	2.919538	0.314098
C	-1.048497	1.785680	0.071440
C	0.358377	1.829443	-0.110722
C	1.000073	3.068007	-0.066143
N	-1.468201	0.455151	-0.053385
C	-0.401722	-0.376171	-0.316358
C	0.804896	0.444743	-0.335598
C	-2.603135	-0.335665	-0.065858
C	-2.110742	-1.636623	-0.349512
N	-0.726109	-1.628525	-0.499290
C	-3.961990	-0.077293	0.133456
C	-4.824512	-1.163228	0.037376
C	-4.353021	-2.461155	-0.244694
C	-3.001585	-2.713266	-0.439048
N	2.002948	-0.039035	-0.557559
C	2.306689	-1.383692	-0.574092
C	2.297925	-1.793628	1.331267
O	3.165232	-2.612632	1.505078
C	3.232277	0.786711	-0.595997
C	4.369034	-0.244187	-0.574002
C	3.716218	-1.537675	-1.083099
O	1.430620	-1.138697	1.847631
H	0.746750	5.191899	0.210421
H	-1.708013	5.061558	0.556211
H	-2.888985	2.859331	0.457868
H	2.078298	3.163067	-0.222825
H	-4.338248	0.926250	0.356850
H	-5.898850	-1.004494	0.187336
H	-5.071025	-3.287052	-0.308574
H	-2.624380	-3.719131	-0.654602
H	1.482854	-2.044607	-0.872033
H	3.204662	1.388782	-1.522475
H	3.235124	1.458898	0.277230
H	4.706671	-0.398875	0.465688
H	5.229801	0.088258	-1.174760
H	3.744434	-1.610269	-2.187613
H	4.180478	-2.438090	-0.651651

TS-ISO

E_0 = -858.419877

E (298 K) = -858.405138

H (298 K) = -1046.920424
G (298 K) = -1046.985806
Imaginary frequencies = 1 (-399 cm^{-1})

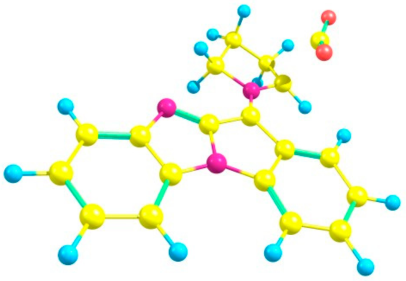

Cartesian coordinates:

C	-0.840399	3.778660	-0.378771
C	0.532181	4.010005	-0.295912
C	1.434666	2.946581	-0.182673
C	0.912076	1.664961	-0.179243
C	-0.476333	1.400269	-0.289953
C	-1.357068	2.478963	-0.363107
N	1.582681	0.437926	-0.057818
C	0.698785	-0.616994	-0.091787
C	-0.645297	-0.060899	-0.257856
C	2.848558	-0.102445	0.063542
C	2.621993	-1.503479	0.094672
N	1.264865	-1.794646	-0.008158
C	4.129148	0.451801	0.146429
C	5.188456	-0.439824	0.263892
C	4.982247	-1.834265	0.297973
C	3.708649	-2.379957	0.215419
N	-1.708479	-0.798550	-0.488043
C	-3.001991	-0.381337	-0.663687
C	-3.562997	0.014675	1.236680
O	-2.631657	0.581766	1.732841
C	-1.627633	-2.286351	-0.481356
C	-3.092708	-2.724316	-0.483976
C	-3.823591	-1.549364	-1.146793
O	-4.680603	-0.405139	1.338132
H	-1.531010	4.627144	-0.439197
H	0.912944	5.037762	-0.303030
H	2.510938	3.123835	-0.093395
H	-2.439863	2.332918	-0.367363
H	4.299123	1.532942	0.122529
H	6.209944	-0.048004	0.332853
H	5.849179	-2.498634	0.392713
H	3.539248	-3.462192	0.242226
H	-3.128525	0.624109	-1.079553
H	-1.077602	-2.581457	-1.393477
H	-1.034097	-2.612648	0.385803
H	-3.453358	-2.833848	0.554178
H	-3.228207	-3.684430	-1.005521
H	-3.809243	-1.627100	-2.251905
H	-4.867321	-1.456639	-0.807486

TS-4c

H (298 K) = -858.404194
G (298 K) = -858.462438
Imaginary frequencies = 1 (-93 cm^{-1})

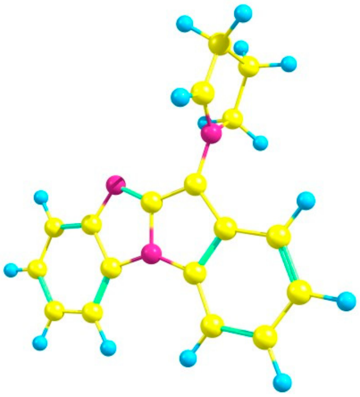

Cartesian coordinates:

H	-3.918283	8.739171	1.152279
H	-3.332881	8.734949	-1.214508
H	-2.046746	7.169281	1.354840
C	-4.076240	7.773080	0.650271
H	-3.046828	6.235044	-2.258292
C	-3.230911	6.632770	-1.252054
C	-1.073422	2.743600	-0.046615
C	-2.283681	3.504001	-0.119252
C	-1.904487	4.874487	-0.285001
N	-0.020052	3.646568	-0.136209
C	-1.077437	1.360073	0.094083
N	-2.743680	6.020932	-0.237176
H	-0.138418	0.797267	0.143805
H	2.147694	1.708073	0.061412
C	-3.957648	7.881214	-0.886297
C	-0.494564	4.952431	-0.250647
C	1.366286	3.732808	-0.100619
C	2.374190	2.778172	-0.006182
C	-2.310520	0.709207	0.176193
C	-3.508874	2.823686	-0.032603
C	1.628517	5.132679	-0.193116
C	3.692342	3.237789	0.000207
N	0.463004	5.873731	-0.283919
H	4.514706	2.516693	0.074599
C	-3.507918	1.437696	0.115767
H	-2.339523	-0.380403	0.290300
C	2.962905	5.562726	-0.183812
C	3.976356	4.610888	-0.087451
H	3.189394	6.633243	-0.253895
H	5.022665	4.941430	-0.080082
H	-4.456544	3.375894	-0.083504
H	-4.464738	0.905436	0.184942
C	-3.013244	6.736534	1.037867
H	-5.079869	7.406313	0.923120
H	-4.920677	7.963624	-1.416865
H	-3.332864	5.996875	1.788965

TS-4c'

E$_0$ = -1513.965775
E (298 K) = -1513.935705
H (298 K) = -1513.934761
G (298 K) = -1514.026287
Imaginary frequencies = 1 (-287 cm^{-1})

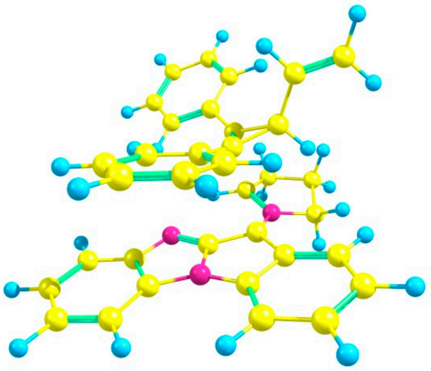

Cartesian coordinates:

N	4.373138	-7.336925	3.173676
N	6.954540	-9.156947	2.882893
N	5.348146	-10.745635	2.728440
C	5.407034	-6.534765	2.930291
C	5.094018	-5.125235	3.335064
C	3.555538	-5.131745	3.364435
C	3.198744	-6.589858	3.678318
C	6.871323	-12.781772	2.507559
C	8.225414	-13.105539	2.453909
C	9.217351	-12.113246	2.553615
C	8.888340	-10.769619	2.712874
C	6.548767	-11.434911	2.662501
C	7.535312	-10.417814	2.769755
C	5.658752	-9.406325	2.857857
C	4.420445	-8.666124	2.876581
C	3.247917	-12.097311	2.434479
C	3.967802	-10.927794	2.642086
C	3.350604	-9.650628	2.758049
C	1.956737	-9.583744	2.632684
C	1.860359	-11.998711	2.327339
C	1.227079	-10.754772	2.422329
H	6.407129	-6.976291	2.868825
H	5.519070	-4.397716	2.621781
H	5.529150	-4.909703	4.330852
H	3.165572	-4.861515	2.366724
H	3.127947	-4.436471	4.103286
H	2.275556	-6.928108	3.187866
H	3.114966	-6.787013	4.763319
H	8.521858	-14.153901	2.331555
H	10.273081	-12.407153	2.507298
H	9.660172	-9.995016	2.790951
H	6.100407	-13.555140	2.421599
H	1.419903	-8.631513	2.684118
H	0.136672	-10.693614	2.326120
H	3.758143	-13.061436	2.336504
H	1.265351	-12.903731	2.158916

E$_0$ = -1513.958743
E (298 K) = -1513.928507
H (298 K) = -1513.927563
G (298 K) = -1514.020494
Imaginary frequencies = 1 (-291 cm^{-1})

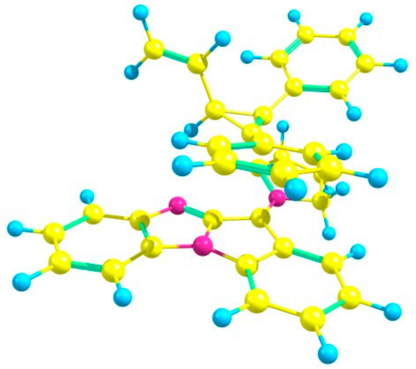

Cartesian coordinates:

N	4.475381	-7.258121	3.527526
N	6.918724	-9.251877	2.986237
N	5.173983	-10.671864	2.710304
C	5.485188	-6.465143	3.155818
C	5.267714	-5.064370	3.648635
C	3.773384	-5.039906	4.047869
C	3.276340	-6.489842	3.895943
C	6.500963	-12.765232	2.104006
C	7.818630	-13.185109	1.930192
C	8.896435	-12.300174	2.110764
C	8.693791	-10.969951	2.467540
C	6.304925	-11.433120	2.465113
C	7.380107	-10.520783	2.647417
C	5.606777	-9.388485	2.992621
C	4.433531	-8.579528	3.226973
C	2.961301	-11.861921	2.614309
C	3.787170	-10.765620	2.819297
C	3.292137	-9.485545	3.197626
C	1.911180	-9.347038	3.390356
C	1.589574	-11.689431	2.807326
C	1.076468	-10.449390	3.198737
H	6.467845	-6.928639	3.011503
H	5.508090	-4.323167	2.866798
H	5.931092	-4.860771	4.509646
H	3.200212	-4.368622	3.388701
H	3.640311	-4.687730	5.083002
H	2.536271	-6.599088	3.080610
H	2.845334	-6.915051	4.818404
H	8.016807	-14.225295	1.646162
H	9.919183	-12.667683	1.963882
H	9.532381	-10.277779	2.606084
H	5.662719	-13.455355	1.959920
H	1.468163	-8.393980	3.694933
H	-0.002356	-10.335383	3.357804
H	3.373047	-12.828296	2.304717
H	0.912545	-12.538043	2.655242

C	3.916694	-6.990828	0.303189	C	6.265987	-7.462356	0.462636
C	5.374470	-6.780042	0.665719	C	4.849580	-7.899932	0.708245
C	4.971784	-8.042428	0.441391	C	5.114663	-6.595301	0.947386
C	5.307776	-9.347421	-0.096101	C	4.622678	-5.260305	0.590535
C	4.290917	-10.257781	-0.422458	C	5.544388	-4.268049	0.223648
C	4.615924	-11.528762	-0.894034	C	5.110302	-2.986445	-0.111553
C	5.953025	-11.903271	-1.037448	C	3.750253	-2.674296	-0.078191
C	6.970088	-10.998024	-0.720944	C	2.825034	-3.657427	0.278586
C	6.651417	-9.726258	-0.257682	C	3.256679	-4.941486	0.606657
H	7.442263	-9.013287	0.003733	H	2.525467	-5.716801	0.868952
H	3.241982	-9.960912	-0.280941	H	6.612421	-4.522883	0.198485
H	3.815438	-12.237749	-1.139878	H	5.842183	-2.222483	-0.401694
H	6.206659	-12.908569	-1.395932	H	3.408956	-1.665372	-0.338787
H	8.022038	-11.294045	-0.821146	H	1.753397	-3.423362	0.293054
C	6.441021	-5.823772	0.365062	C	3.996463	-8.936659	0.164780
C	6.131678	-4.582713	-0.207856	C	4.611213	-10.090615	-0.351209
C	7.147341	-3.671805	-0.498823	C	3.842509	-11.135270	-0.858527
C	8.475805	-3.983619	-0.207347	C	2.449000	-11.047500	-0.846618
C	8.785882	-5.211560	0.382870	C	1.828368	-9.907280	-0.328604
C	7.776753	-6.127102	0.672133	C	2.593456	-8.860876	0.175668
H	8.006016	-7.087620	1.157078	H	2.104636	-7.976070	0.603669
H	9.272988	-3.265740	-0.434845	H	1.841391	-11.872202	-1.239014
H	9.827466	-5.457218	0.623828	H	0.733614	-9.840683	-0.305582
H	5.083462	-4.344996	-0.433824	H	5.708561	-10.152907	-0.337939
H	6.897517	-2.706695	-0.956676	H	4.336091	-12.028958	-1.260181
C	3.397474	-6.635001	-1.046073	C	6.731875	-7.362441	-0.949587
H	3.166906	-6.892781	1.112740	H	7.046221	-7.749697	1.189633
C	2.158134	-6.198018	-1.283090	C	7.982493	-7.616129	-1.343456
H	4.109653	-6.753513	-1.878796	H	5.970419	-7.063959	-1.689586
H	1.434453	-6.077841	-0.463927	H	8.751388	-7.920524	-0.619026
H	1.818901	-5.950838	-2.296097	H	8.281251	-7.536165	-2.395818

TS-5c

E_0 = -1513.963183
E (298 K) = -1513.932955
H (298 K) = -1513.932010
G (298 K) = -1514.024776
Imaginary frequencies = 1 (-312 cm^{-1})

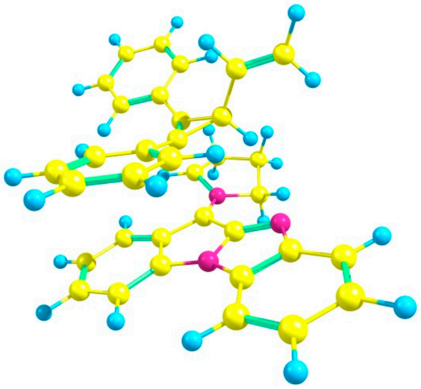

Cartesian coordinates:

N	3.827058	-7.948855	2.756456
N	6.970084	-8.559348	2.678407
N	6.163683	-10.652391	2.986302

TS-5c'

E_0 = -1513.958306
E (298 K) = -1513.928179
H (298 K) = -1513.927235
G (298 K) = -1514.018797
Imaginary frequencies = 1 (-307 cm^{-1})

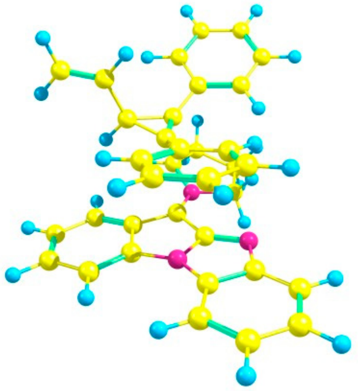

Cartesian coordinates:

N	3.833562	-8.003948	2.667674
N	6.982935	-8.424845	2.373292
N	6.356075	-10.525473	2.930641

C	2.700858	-7.587991	3.362606	C	2.686869	-7.700835	3.276458
C	2.307853	-6.193638	2.969380	C	2.186466	-6.349457	2.853533
C	3.657180	-5.606607	2.515636	C	3.381985	-5.739976	2.083417
C	4.419861	-6.823601	1.984737	C	4.499324	-6.793030	2.142330
C	8.392128	-11.888286	3.104374	C	8.674513	-11.587350	3.015542
C	9.759724	-11.637613	3.026205	C	10.012358	-11.246341	2.839007
C	10.253452	-10.336389	2.814573	C	10.393813	-9.937918	2.484159
C	9.399208	-9.247041	2.679862	C	9.451363	-8.933089	2.293057
C	7.539857	-10.792862	2.961158	C	7.734527	-10.573607	2.826810
C	8.018201	-9.469355	2.758434	C	8.097794	-9.248426	2.467139
C	5.893986	-9.308011	2.820203	C	5.976692	-9.225606	2.660010
C	4.467730	-9.130332	2.965538	C	4.549280	-9.135586	2.880529
C	4.817227	-12.740130	3.398803	C	5.181440	-12.681744	3.495977
C	4.987813	-11.379691	3.196945	C	5.245978	-11.324129	3.219049
C	3.902283	-10.462149	3.157763	C	4.091280	-10.497495	3.149375
C	2.607190	-10.966227	3.308597	C	2.838250	-11.099569	3.300354
C	3.512033	-13.216061	3.555954	C	3.917744	-13.248808	3.685242
C	2.425684	-12.339774	3.502404	C	2.764023	-12.468994	3.570446
H	2.008685	-8.341454	3.742051	H	2.035498	-8.505126	3.627933
H	1.848078	-5.652127	3.814337	H	1.894434	-5.747588	3.732323
H	1.567238	-6.219188	2.145033	H	1.288996	-6.451311	2.215958
H	4.186585	-5.183449	3.388368	H	3.713821	-4.793521	2.539529
H	3.555861	-4.818391	1.753585	H	3.106981	-5.527852	1.037561
H	5.511660	-6.807821	2.131736	H	5.321555	-6.539329	2.836768
H	4.208198	-7.015779	0.916060	H	4.963816	-7.030242	1.171806
H	10.464434	-12.470919	3.130587	H	10.783989	-12.012164	2.980430
H	11.337451	-10.179863	2.757974	H	11.458600	-9.709402	2.354517
H	9.781337	-8.232106	2.521397	H	9.744591	-7.912951	2.019368
H	8.009709	-12.901345	3.270271	H	8.381137	-12.603541	3.300276
H	1.729171	-10.312091	3.261426	H	1.911896	-10.524258	3.193832
H	1.408208	-12.732563	3.615385	H	1.780148	-12.936811	3.692569
H	5.676674	-13.418009	3.437366	H	6.090492	-13.289681	3.555325
H	3.343387	-14.286484	3.720072	H	3.834190	-14.318498	3.908620
C	5.005813	-7.245662	5.340433	C	3.110941	-8.980901	5.937629
C	3.548648	-7.638077	5.479550	C	4.469677	-8.624303	5.407353
C	4.458876	-8.631042	5.481791	C	3.543852	-7.639513	5.376730
C	4.903875	-9.907750	6.007256	C	3.332074	-6.244519	5.769541
C	6.278235	-10.191432	6.015151	C	2.152174	-5.897133	6.444567
C	6.738902	-11.424445	6.471784	C	1.903176	-4.572228	6.799058
C	5.832204	-12.384918	6.924980	C	2.822412	-3.573475	6.474401
C	4.462485	-12.107102	6.923834	C	4.000512	-3.911419	5.804544
C	3.998802	-10.877194	6.468189	C	4.256144	-5.236172	5.456407
H	2.924063	-10.659833	6.455125	H	5.185548	-5.497789	4.934498
H	6.977784	-9.432022	5.636145	H	1.431881	-6.688119	6.692942
H	7.815081	-11.637979	6.465588	H	0.979137	-4.316112	7.331704
H	6.194125	-13.356856	7.282238	H	2.623792	-2.530327	6.747939
H	3.748608	-12.861821	7.275907	H	4.731885	-3.133146	5.553692
C	2.360453	-7.132423	6.165830	C	5.821846	-9.057283	5.701701
C	2.308046	-5.790028	6.569057	C	6.011208	-10.366651	6.178438
C	1.174091	-5.290364	7.208251	C	7.294044	-10.842583	6.437851
C	0.074748	-6.118594	7.440638	C	8.402102	-10.020947	6.221360
C	0.116206	-7.453268	7.031607	C	8.221394	-8.715695	5.756906

C	1.249521	-7.957890	6.398042	C	6.941974	-8.235471	5.493644
H	1.281182	-9.006760	6.071036	H	6.802067	-7.222265	5.097701
H	-0.817553	-5.723282	7.940374	H	9.414547	-10.400264	6.406974
H	-0.745124	-8.108830	7.209789	H	9.091528	-8.072329	5.577238
H	3.177138	-5.145038	6.379404	H	5.129397	-11.004549	6.331321
H	1.147524	-4.240757	7.526107	H	7.431883	-11.867187	6.805118
C	5.740676	-6.628632	6.479238	C	2.948821	-9.166755	7.407361
H	5.380051	-6.941610	4.345396	H	2.437112	-9.616185	5.331929
C	6.775764	-5.797188	6.337395	C	2.089567	-10.023917	7.964311
H	5.384618	-6.902603	7.485869	H	3.599415	-8.544279	8.043325
H	7.148571	-5.526356	5.339282	H	1.439125	-10.655454	7.342411
H	7.292599	-5.370186	7.205293	H	2.008174	-10.131001	9.052837

6. References

[1] Neuhaus D., Williamson M. P. The nuclear Overhauser effect in structural and conformational analysis, 2nd edition / N.Y.: VCH Publishers Inc. 2000. – 619 P.

[2] Bell R. A., Saunders J .K. Correlation of the nuclear Overhauser effect with internuclear distance // Canad. J. Chem. 1970. – Vol. 48. – № 7. – P. 1114–1122.

[3] Andersen N. H., Eaton H. L., Lai X. Quantitative Small Molecule NOESY. A Practical Guide for Derivation of Cross-relaxation Rates and Internuclear Distances // Magn. Reson. Chem. 1989. – Vol. 27. – № 6. – P. 515–528.

[4] Woessner D. E. Spin Relaxation Processes in Two-Proton System Undergoing Anisotropic Reorientation // J. Chem. Phys. 1962. – Vol. 36. – № 1. – P. 1–4.

[5] Landy S. B., Rao B. D. N. Influence of Molecular Geometry on Uncertainty in Distances Determined from NOE // J. Magn. Reson. 1989. – Vol. 83. – № 1. – P. 29–43.

[6] Liu H., Thomas P. D., James T. L. Averaging of Cross-Relaxation Rates and Distances for Methyl, Methylene and Aromatic Ring Protons due to Motion or Overlap. Extraction of Accurate Distances Iteratively via Relaxation Matrix Analysis of 2D NOE Spectra // J. Magn. Reson. 1992. – Vol. 98. – № 1. – P. 163–175.

[7] Lee W., Krishna N. Influence of conformational exchange on the 2D NOESY spectra of biomolecules existing in multiple conformations // J. Magn. Reson. 1992. – Vol. 98, – № 1. – P. 36–48.

[8] Tropp J. Dipolar relaxation and nuclear Overhauser effects in nonrigid molecules: The effect of fluctuating internuclear distances // J. Chem. Phys. 1980. – Vol. 72. – № 11. – P. 6035–6043.

[9] Butts C. P., Jones C. R., Harvey J. N. High precision NOEs as a probe for low level conformers– second conformation of strychnine // Chem. Commun. 2011. – Vol. 47. – № 4. – P. 1193–1195.

[10] Butts C. P., Jones C. R., Song Z., Simpson T. J. Accurate NOE-distance determination enables the stereochemical assignment of a flexible molecule – arugosin C // Chem. Commun. 2012. – Vol. 48. – № 4. – P. 9023–9025.

[11] Peverati, R.; Truhlar, D. G. *J. Phys. Chem. Lett.* **2011**, *2*, 2810−2817.

[12] Dunning, T. H. *J. Chem. Phys.* **1989**, *90*, 1007−1023.

[13] Cossi, M.; Rega, N.; Scalmani, G.; Barone, V. *J. Comput. Chem.* **2003**, *24*, 669−681.

[14] Schlegel, H. B. *J. Comput. Chem.* **1982**, *3*, 214−218.

[15] Fukui, K. J. *J. Phys. Chem.* **1970**, *74*, 4161−4163.

[16] Frisch, M. J.; Trucks, G. W.; Schlegel, H. B.; Scuseria, G. E.; Robb, M. A.; Cheeseman, J. R.; Scalmani, G.; Barone, V.; Mennucci, B.; Petersson, G. A.; Nakatsuji, H.; Caricato, M.; Li, X.; Hratchian, H. P.; Izmaylov, A. F.; Bloino, J.; Zheng, G.; Sonnenberg, J. L.; Hada, M.; Ehara, M.; Toyota, K.; Fukuda, R.; Hasegawa, J.; Ishida, M.; Nakajima, T.; Honda, Y.; Kitao, O.; Nakai, H.; Vreven, T.; The Journal of Organic Chemistry Article DOI: 10.1021/acs.joc.9b00753 J. Org. Chem. 2019, 84, 7017−7036 7035 Montgomery, J. A.; Peralta, J. E.; Ogliaro, F.; Bearpark, M.; Heyd, J. J.; Brothers, E.; Kudin, K. N.; Staroverov, V. N.; Kobayashi, R.;

Normand, J.; Raghavachari, K.; Rendell, A.; Burant, J. C.; Iyengar, S. S.; Tomasi, J.; Cossi, M.; Rega, N.; Millam, N. J.; Klene, M.; Knox, J. E.; Cross, J. B.; Bakken, V.; Adamo, C.; Jaramillo, J.; Gomperts, R.; Stratmann, R. E.; Yazyev, O.; Austin, A. J.; Cammi, R.; Pomelli, C.; Ochterski, J. W.; Martin, R. L.; Morokuma, K.; Zakrzewski, V. G.; Voth, G. A.; Salvador, P.; Dannenberg, J. J.; Dapprich, S.; Daniels, A. D.; Farkas, Ő.; Foresman, J. B.; Ortiz, J. V.; Cioslowski, J.; Fox, D. J.. Gaussian 09, revision C.01; Gaussian: Wallingford, CT, 2013.
